# Understanding the Degradation of Methylenediammonium and Its Role in Phase-Stabilizing Formamidinium Lead Triiodide

**DOI:** 10.1021/jacs.3c01531

**Published:** 2023-04-28

**Authors:** Elisabeth A. Duijnstee, Benjamin M. Gallant, Philippe Holzhey, Dominik J. Kubicki, Silvia Collavini, Bernd K. Sturdza, Harry C. Sansom, Joel Smith, Matthias J. Gutmann, Santanu Saha, Murali Gedda, Mohamad I. Nugraha, Manuel Kober-Czerny, Chelsea Xia, Adam D. Wright, Yen-Hung Lin, Alexandra J. Ramadan, Andrew Matzen, Esther Y.-H. Hung, Seongrok Seo, Suer Zhou, Jongchul Lim, Thomas D. Anthopoulos, Marina R. Filip, Michael B. Johnston, Robin J. Nicholas, Juan Luis Delgado, Henry J. Snaith

**Affiliations:** † Clarendon Laboratory, Department of Physics, University of Oxford, Parks Road, Oxford OX1 3PU, United Kingdom; ‡ Department of Physics, University of Warwick, Coventry CV4 7AL, United Kingdom; § POLYMAT, University of the Basque Country UPV/EHU, Avenida de Tolosa 72, 20018 Donostia−San Sebastián, Spain; ⊥ ISIS Facility, STFC Rutherford Appleton Laboratory, Harwell Science and Innovation Campus, Chilton, Didcot, Oxfordshire OX11 0QX,United Kingdom; ▲ King Abdullah University of Science and Technology (KAUST), KAUST Solar Center (KSC), Thuwal 23955-6900, Saudi Arabia; ∇ Research Center for Advanced Materials, National Research and Innovation Agency (BRIN), South Tangerang 15314, Banten, Indonesia; ○ Department of Physics and Astronomy, The University of Sheffield, Hicks Building, Hounsfield Road, Sheffield S3 7RH, United Kingdom; ◆ Department of Earth Sciences, University of Oxford, 3 South Parks Road, Oxford OX1 3AN, United Kingdom; ¶ Graduate School of Energy Science and Technology (GEST), Chungnam National University, 99 Daehak-ro, Yuseong-gu, Daejeon 34134, Korea; ⋈ Ikerbasque, Basque Foundation for Science, 48013 Bilbao, Spain

## Abstract

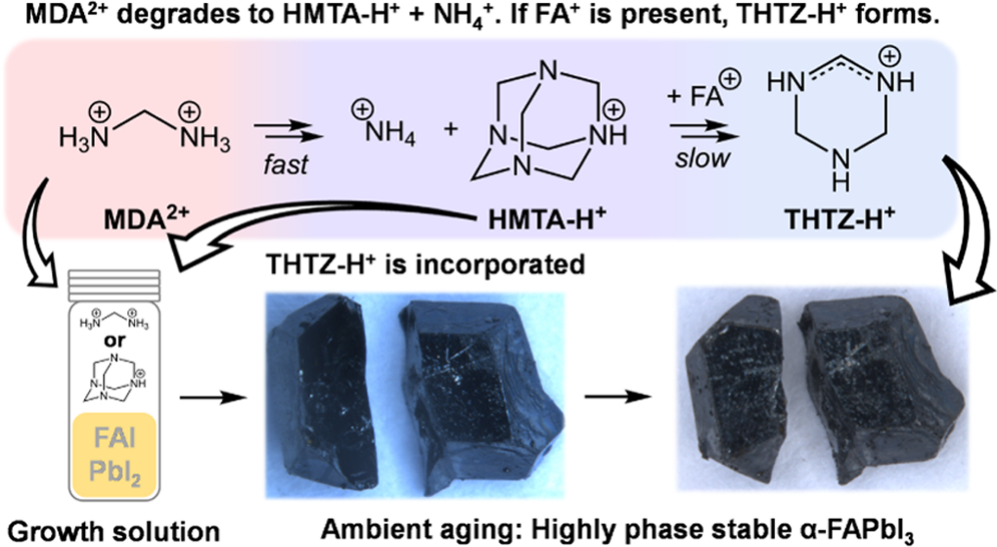

Formamidinium lead triiodide (FAPbI_3_) is the leading candidate for single-junction metal–halide perovskite photovoltaics, despite the metastability of this phase. To enhance its ambient-phase stability and produce world-record photovoltaic efficiencies, methylenediammonium dichloride (MDACl_2_) has been used as an additive in FAPbI_3_. MDA^2+^ has been reported as incorporated into the perovskite lattice alongside Cl^–^. However, the precise function and role of MDA^2+^ remain uncertain. Here, we grow FAPbI_3_ single crystals from a solution containing MDACl_2_ (FAPbI_3_-M). We demonstrate that FAPbI_3_-M crystals are stable against transformation to the photoinactive δ-phase for more than one year under ambient conditions. Critically, we reveal that MDA^2+^ is not the direct cause of the enhanced material stability. Instead, MDA^2+^ degrades rapidly to produce ammonium and methaniminium, which subsequently oligomerizes to yield hexamethylenetetramine (HMTA). FAPbI_3_ crystals grown from a solution containing HMTA (FAPbI_3_-H) replicate the enhanced α-phase stability of FAPbI_3_-M. However, we further determine that HMTA is unstable in the perovskite precursor solution, where reaction with FA^+^ is possible, leading instead to the formation of tetrahydrotriazinium (THTZ-H^+^). By a combination of liquid- and solid-state NMR techniques, we show that THTZ-H^+^ is selectively incorporated into the bulk of both FAPbI_3_-M and FAPbI_3_-H at ∼0.5 mol % and infer that this addition is responsible for the improved α-phase stability.

## Introduction

Hybrid organic–inorganic metal–halide perovskites are recognized as one of the most promising emerging semiconducting materials for optoelectronic applications due to their excellent properties, including tunable band gaps,^1^ high absorption coefficients,^2^ and long charge-carrier diffusion lengths.^3^ Among the ABX_3_ lead–halide perovskites reported to date, FAPbI_3_ (FA^+^ is formamidinium, HC(NH_2_)_2_^+^)^4^ has the narrowest achievable band gap, allowing for the highest theoretical photovoltaic power conversion efficiency (PCE) in a single-junction architecture.^5^ For this reason, the majority of recent world-record PCE perovskite photovoltaics have employed FAPbI_3_-based materials as their photoabsorbing layer.^6−9^ However, under ambient conditions, the cubic α-phase of FAPbI_3_ is thermodynamically unstable with respect to transformation to a photoinactive hexagonal δ-phase polytype. Despite this, the phase transition is kinetically inhibited under ambient conditions permitting metastable α-FAPbI_3_ to persist for hours, days, and even months, depending strongly on processing conditions and the atmospheric conditions in which the processed material is stored.^10,11^ The phase stability of FAPbI_3_ thin films and single crystals is necessary for their practical use. Significant efforts are directed at finding approaches to suppress the α-to-δ phase transition. In particular, smaller A-site cations have been used to stabilize the perovskite lattice by alloying with FA^+^. Most frequently used are MA^+^ (but which has been shown to introduce an unfavorable thermal instability^12,13^) and the alkali-metal Cs^+^,^14^^15^ often in conjunction with I^–^, Br^–^, and Cl^–^ X-site alloying. Although promising, such approaches can introduce undesirable properties. Band gap increases induced by structural changes due to the inclusion of smaller cations^16^ are undesirable for single-junction photovoltaics. Furthermore, compositional inhomogeneities in the mixed-ion perovskites introduced during growth can render materials susceptible to ion segregation^17,18^ and non-radiative recombination losses^19^ under operation. It is therefore highly valuable to develop strategies to improve the stability of FAPbI_3_ without diminishing its valuable properties.

In 2019, Min et al.^20^ reported highly efficient α-FAPbI_3_ solar cells based on polycrystalline thin films by incorporating a small amount of methylenediammonium dichloride (MDACl_2_, 3.8 mol %) into the precursor solution while maintaining the inherent band gap of FAPbI_3_. The authors attribute the high-certified PCE of 23.7% to the addition of MDACl_2_ and report that MDA^2+^ leads to structural stabilization of α-FAPbI_3_ via partial replacement of FA^+^ with MDA^2+^ alongside Cl^–^ incorporation at interstitial sites. Subsequently, Kim et al.^21^ propose that concurrent substitution of 3 mol % of Cs^+^ and MDA^2+^ on FA^+^ sites lowers the lattice strain and trap density in perovskite solar cells. In more recent works—again from Seok and co-workers—MDA^2+^ was twice used alongside FAPbI_3_ to achieve the highest yet-reported certified efficiency for perovskite solar cells, 25.5%^9^ and subsequently 25.7%.^8,22^

In order to isolate the role of MDACl_2_, we here study the effect of its addition to the precursor solution during the growth of FAPbI_3_ single crystals (denoted FAPbI_3_-M). We find that the highly acidic MDA^2+^ cation is unstable in solution, degrading rapidly into ammonium and hexamethylenetetramine (HMTA). However, this degradation is further complicated by the presence of FA^+^ in the precursor solution, a reaction which can interrupt the degradation pathway and instead lead to the formation of tetrahydro-1,3,5-triazinium (THTZ-H^+^). We find that of all the degradation products formed, it is THTZ-H^+^ that is present in FAPbI_3_-M crystals. We show that FAPbI_3_-M crystals grown possess vastly improved α-phase stability (>1 year in the air) and significantly reduced defect density.

## Results and Discussion

### Single-Crystal Growth and Optoelectronic Performance

We grow the single crystals via the inverse temperature crystallization method.^23,24^ The control FAPbI_3_ single crystals are prepared by dissolving equimolar FAI and PbI_2_ in γ-butyrolactone (GBL). Crystal growth is directed by the addition of an appropriate seed crystal to this precursor solution. Heating to 95 °C leads to growth of the seed into an α-FAPbI_3_ single crystal between 2 and 4 mm in length. For the FAPbI_3_-M single crystals, we add 3.8 mol % (with respect to the Pb content) of MDACl_2_ to the FAPbI_3_ perovskite precursor solution. We note that in contrast to Min et al.,^20^ we do not add MACl to any of our precursor solutions, as we aim to isolate and investigate the effect of MDACl_2_ on FAPbI_3_-phase stability. Further details on crystal growth are discussed in Supporting Note 1. We heat the single crystals in a vacuum oven at 180 °C for 30 min to remove residual solvent on the crystal surface. Single-crystal X-ray diffraction (SCXRD) confirms the three-dimensional (3D) perovskite phase in each case, with crystal structures solved in the *Pm*3̅*m* cubic space group, as previously reported.^25^ Crystal data and structure refinement statistics are shown in Table S1. We discuss our SCXRD measurements in detail in Supporting Note 2, including our observation of a pronounced difference in the degree of twinning detected in FAPbI_3_ crystals grown in the presence of different additives.

**Figure 1:**
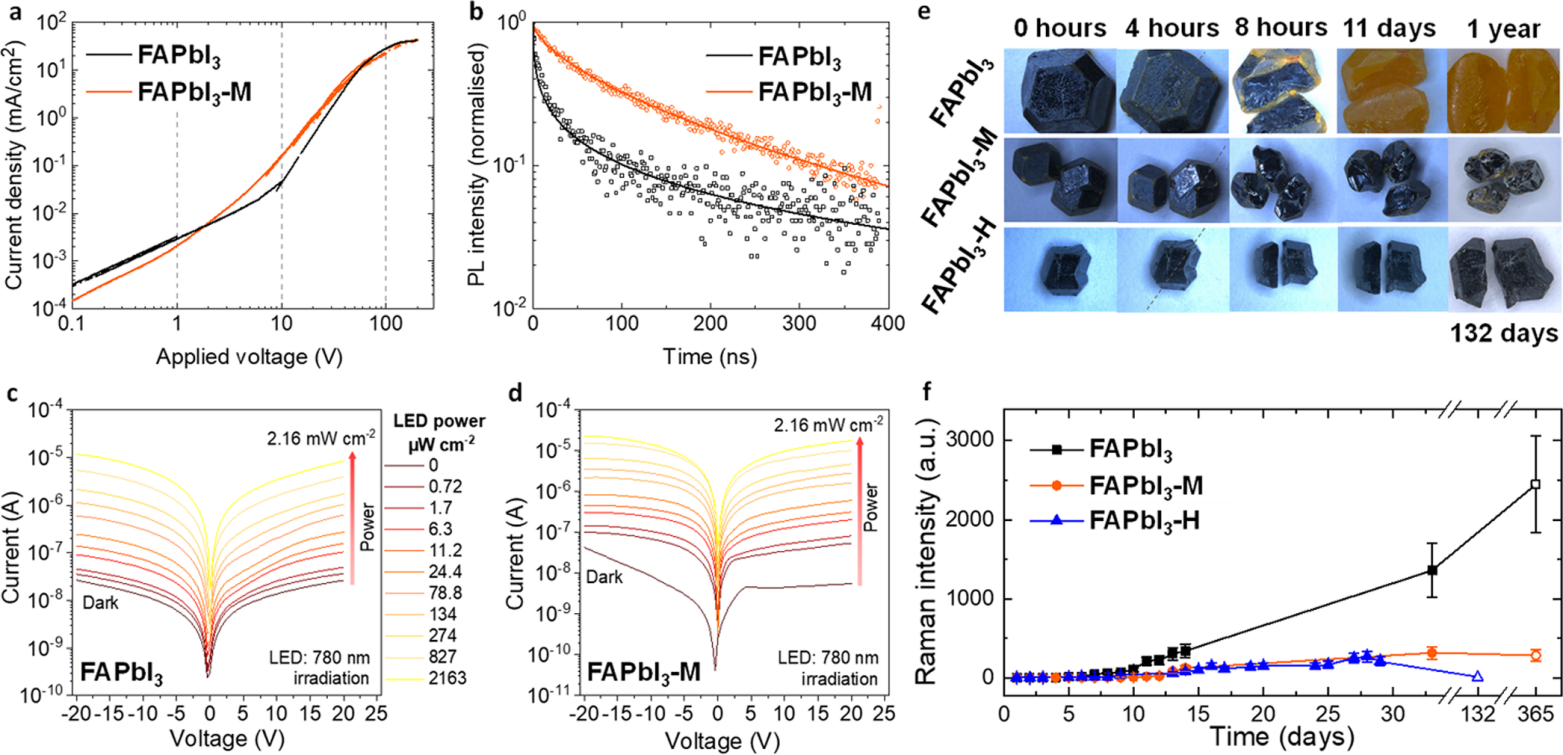
Single-crystal optoelectronics and stability. (a) PV-SCLC measurements for FAPbI_3_ and FAPbI_3_-M single crystals; (b) time-resolved photoluminescence measurements of FAPbI_3_ and FAPbI_3_-M. Current density–voltage curves in a N_2_ environment of (c) FAPbI_3_ and (d) FAPbI_3_-M single-crystal photodetectors with Ag electrodes. (e) Optical microscope images of grown FAPbI_3_, FAPbI_3_-M, FAPbI_3_-H crystals. The crystals are cleaved after 4 h. The crystals were kept in glass vials under ambient conditions (dark, relative humidity ∼30 to 80%) throughout the measurement period. (f) Temporal evolution of δ-phase Raman peak intensity for FAPbI_3_, FAPbI_3_-M, FAPbI_3_-H single crystals. The single crystals were kept in the air for the first 33 days. The long-term data at 132 and 365 days (no fill) correspond to vial-stored crystals.

We first investigate the impact of MDACl_2_ on the electronic properties of the FAPbI_3_ single crystals. To investigate the charge transport through the single crystals, we deposit gold electrodes on the crystals and perform pulsed-voltage space-charge-limited-current (PV-SCLC) measurements in which the dark current–voltage (*J*–*V*) characteristics are measured under vacuum, shown in Figure 1a.^26^ The pulsed *J*–*V* traces of both samples show an Ohmic region at low voltages, where the slope, , equals 1. From this region, we estimate a dark DC conductivity of 8.5 × 10^–6^ and 5.3 × 10^–6^ S m^–1^ for FAPbI_3_ and FAPbI_3_-M crystals, respectively. We have previously shown that if the density of traps is larger than the density of static ionic space charge, the *J*–*V* curve deviates from being linear into a regime where *m* is larger than 2.^26−28^ From the voltage point at which this occurs, (*V*_ons_), we can calculate a lower bound trap density (*n*_t_) via: , where ε_0_ is the vacuum permittivity, *L* the crystal thickness, *e* the electron charge, and ε_r_ is the low-frequency dielectric constant of the material, reported as 49.4 for FAPbI_3_.^29^ We assume the same value for ε_r_ for FAPbI_3_-M. We find that adding MDACl_2_ to the precursor solution significantly reduces the trap density from a lower bound of 1.1 × 10^12^ cm^–3^ for FAPbI_3_ to 4.3 × 10^10^ cm^–3^ for FAPbI_3_-M.

The improvement in optoelectronic quality of these FAPbI_3_-M single crystals is supported by time-resolved photoluminescence (TRPL) measurements (depicted in Figure 1b). Fitting the TRPL decay traces reveals a significant increase in a lifetime from 47 ns for the FAPbI_3_ single crystal to 121 ns for the FAPbI_3_-M single crystal at a fluence of 18 nJ cm^–2^. More details on this measurement and the fitting are given in the Supporting Note 3. To assess if FAPbI_3_-M crystals also improve optoelectronic devices, we fabricate photodetectors from the single crystals. Figure 1c,d shows the current density curve of FAPbI_3_ and FAPbI_3_-M single-crystal detectors. We observe a higher photoconductivity for FAPbI_3_-M photodetectors and an improved ON/OFF ratio (Supporting Figure S7).

Significantly, we also find that FAPbI_3_-M crystals possess substantially greater α-phase (black) stability in ambient air than neat FAPbI_3_. By visible light microscopy, we observe the growth of trace regions of a δ-phase (yellow) on the crystal surface of both materials after only a few hours of storage in the air (Figure 1e). For FAPbI_3_-M, the degradation does not propagate significantly on the surface or into the bulk, as seen when we cleave the crystals after several hours. Notably, FAPbI_3_-M crystals remain predominantly in their α-phase after one year of storage in ambient air. By contrast, FAPbI_3_ crystals display complete phase transformation to the photoinactive δ-phase within a few days (Figure 1e and Supporting Figure S8).

To quantitatively track the phase stability of the crystals, we monitor the absolute Raman intensity of the best resolved δ-phase peak (at a Raman shift of 108 cm^–1^) under ambient conditions. As Figure 1f shows (complete Raman spectra shown in Figure S9), neat FAPbI_3_ undergoes detectable δ-phase formation after 7 days. This onset occurs at 11 days for FAPbI_3_-M. In Raman spectra of the same crystals after 1 year of storage in air-filled vials, we detect (2.5 ± 0.5) × 103 and (0.25 ± 0.06) × 103 δ-phase peak counts for FAPbI_3_ and FAPbI_3_-M, respectively. Peak intensity is proportional to the fraction of the probed surface layer in the δ-phase. Notably, the thickness of the probed layer for α-phase FAPbI_3_ is estimated to be ∼100 nm via the absorption coefficient (ca. 10^5^ cm^–1^ at the Raman laser wavelength of 532 nm).^30^ Thus, the onset of the δ-phase peak in Figure 1f corresponds to the degradation of only the top surface of the crystals. We obtain complementary degradation data of the bulk crystal by carrying out the same Raman measurement on the exposed interior of freshly cleaved crystals, different from those presented in Figure 1f. Cleaving a FAPbI_3_-M crystal after 285 days of aging gives a peak intensity ratio of *I*_bulk_/*I*_surface_ = 0.10 ± 0.03 for the δ-phase peak. This confirms that the observed δ-phase formation occurs predominantly in a thin surface layer of FAPbI_3_-M crystals, while the bulk remains largely unaffected. By contrast, the neat FAPbI_3_ crystal is entirely converted to the δ-phase.

### MDA^2+^ Solution Instability

**Figure 2:**
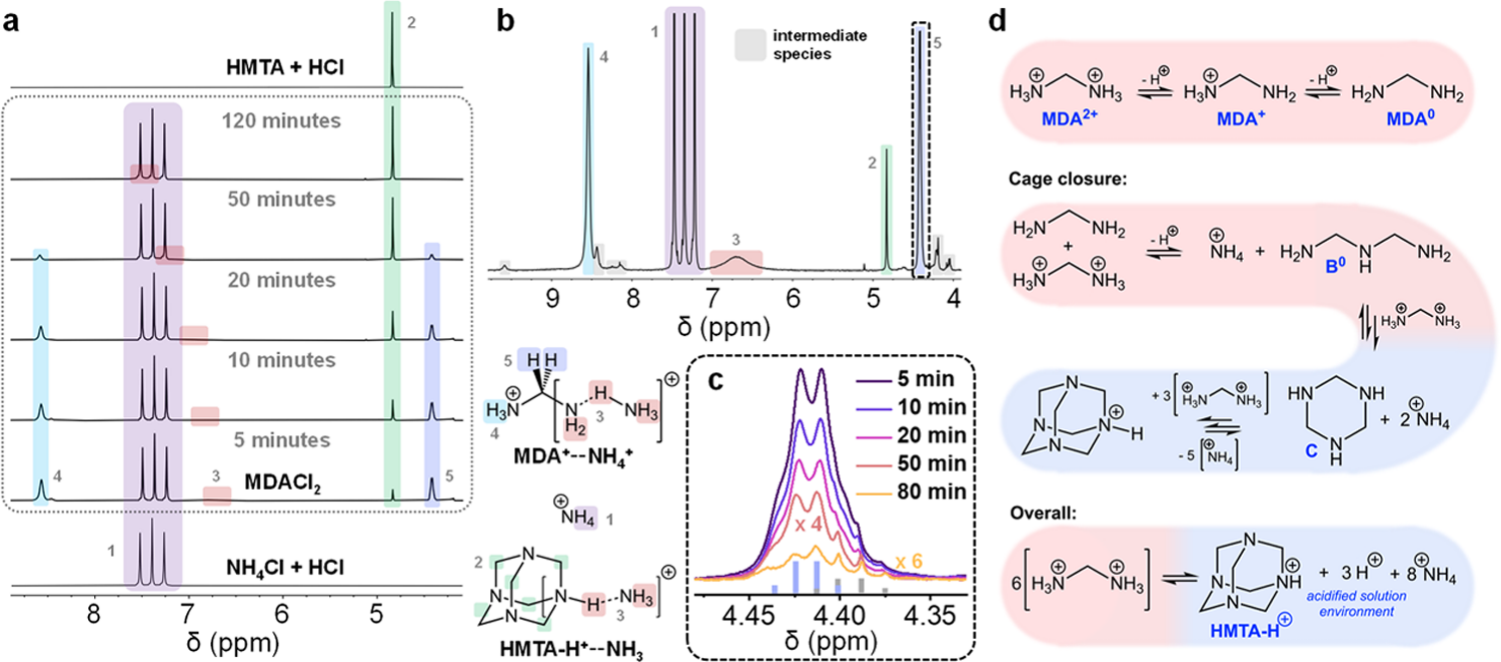
Degradation of MDA^2+^. (a) ^1^H solution nuclear magnetic resonance (NMR) spectra tracking the evolution of MDACl_2_ upon dissolution in DMSO-*d*_6_. Bottom: spectra acquired from acidified solution of ammonium chloride. Top: spectra acquired from acidified solution of (HMTA). Color scheme correlates detected signals with ^1^H chemical environments in detected species. (b) Expanded ^1^H NMR spectrum of 5 min after the initial dissolution of MDACl_2_ in DMSO-*d*_6_. (c) Evolution of ^1^H NMR quartet signal corresponding to methylene environment in dominant degradation intermediate. (d) Degradation pathway of MDA^2+^ upon dissolution in a polar solvent. Hexamethylenetetramine (HMTA) and ammonia are represented in their protonated forms in line with our observations and on account of the acidic environment generated in solution as a result of the degradation.

Having established the substantial advantages of MDACl_2_ addition for FAPbI_3_ single-crystal properties, we now investigate the activity of this additive in solution. It has been previously inferred that MDA^2+^ is incorporated within the cubic ABX_3_ perovskite lattice on the A-site in solution-processed polycrystalline thin films based on FAPbI_3_, and thus, A-site cation mixing is thought to be responsible for the enhanced α-phase stability.^20,21^ However, we find that MDA^2+^ degrades rapidly in precursor solutions. Figure 2a shows the ^1^H solution NMR spectra acquired from a solution of MDACl_2_ dissolved in DMSO-*d*_6_ between 5 and 120 min after initial dissolution (expanded spectra shown in Supporting Figure S11). In Figure 2b, we show an expanded view of the earliest of these spectra, emphasizing the presence of a number of signals that appear to correspond to intermediate species that exist only transiently. At the shortest time after dissolution, the ^1^H NMR spectrum already shows several unexpected species. We attribute the distinctive 1:1:1 triplet at 7.34 ppm to NH_4_^+^, with the splitting due to spin–spin coupling of ^1^H to the quadrupolar (*I* = 1) ^14^N nucleus (^1^*J*_^14^N–^1^H_ = 50.8 Hz). The signal, initially at 7.34 ppm, rapidly shifts and stabilizes at 7.37 ppm, as is typical for ammonium species under varying pH conditions.^31^ This assignment is confirmed by comparison with the solution ^1^H NMR spectra of NH_4_Cl in DMSO-*d*_6_ (Supporting Figure S12). ^1^H–^1^H correlation spectroscopy (COSY), depicted in Supporting Figure S13, reveals that the two other substantial signals initially present in the ^1^H solution NMR of MDACl_2_ solutions are spin–spin coupled and thus correspond to nuclei present in the same species. Figure 2c highlights the coupling of one of these signals (4.42 ppm), which we find is superposed on top of another low-intensity signal, both of which we interpret as quartets. Given C–C bond formation is unlikely under the conditions, the resolution of this spin-coupling along with the chemical shift suggests a CH*_n_* environment adjacent to a ^+^NH_3_ group. In Supporting Note 6, we present the findings from a series of further experiments investigating the evolution of intermediate degradation species and discuss our interpretation. From all these data, we infer that the most likely assignment for the dominant intermediate observed is MDA^+^ in which the amine group is undergoing rapid chemical exchange with acidic NH_4_^+^ in solution (Figure 2b). The accumulation of this species is mechanistically justified. When the charge-dense MDA^2+^ cation is dissolved in pH neutral, aprotic polar solvents such as dimethylformamide (DMF), dimethyl sulfoxide (DMSO), and GBL, it is expected to act as a Brønsted acid, rapidly releasing H^+^ to become MDA^+^ and acidifying the solution environment, as shown in Figure 2d. Release of a second acidic H^+^ is possible, although less favorable in the acidified solution environment. However, this second deprotonation event yields neutral MDA^0^, a potent nucleophile. The presence of both MDA^0^ and MDA^2+^, a strong electrophile, renders both species unstable in solution with respect to the formation of NH_4_^+^ and bis(aminomethyl)ammonium (**B^+^**). Thus, under these conditions, MDA^+^ is the most stable form of this species. MDA^+^ can also act as an electrophile; however, its single charge renders it less reactive than MDA^2+^. It may also eliminate to produce NH_3_ and methaniminium, CH_2_=NH_2_^+^. However, elimination reactions are typically slow, and the production of methaniminium—itself a highly reactive electrophile—does not alter the course of the degradation. We propose a complete mechanistic description in Supporting Figure S15.

Transient deprotonation of **B**^**+**^ produces **B**^**0**^, another nucleophile, which reacts with further MDA^2+^ before rapidly cyclizing to form 1,3,5-triazinane (**C**). Repeated reaction of **C** with MDA^2+^ in conjunction with rapid cage-closure, driven entropically by the release of additional NH_4_^+^, is expected to lead to the formation of HMTA-H^+^, as shown in Figure 2d. Experimentally, between 5 and 50 min after the initial dissolution of MDACl_2_, a singlet signal of increasing intensity is observed in the ^1^H solution NMR spectra at 4.83 ppm. By 120 min after initial dissolution, the spectrum consists only of this singlet, the 1:1:1 triplet corresponding to ammonium, and a broad singlet at 8.00 ppm. HMTA dissolved in DMSO-*d*_6_ at neutral pH shows a singlet at 4.56 ppm (Supporting Figure S16). However, incremental addition of hydrochloric acid to this solution produces a downfield shift of this singlet to 4.83 ppm in line with the reported monoprotic p*K*_aH_ value for HMTA (4.93),^32^ confirming the identity of the final product of MDA^2+^ degradation as HMTA-H^+^. Integration of the relevant ^1^H NMR signals confirms the stoichiometric 8:1 ratio of NH_4_^+^/HMTA-H^+^ expected by the proposed degradation route. As observed with MDA^+^, rapid chemical exchange between the acidic H^+^ of HMTA-H^+^ and ammonium leads to a broadened ^1^H NMR signal at a chemical shift corresponding to the weighted average of the contributing chemical environments (7.45 ppm). We emphasize that despite the bias toward the formation of HMTA-H^+^ and NH_4_^+^, as all reaction steps are mechanistically reversible, it should be expected that the system at dynamic equilibrium will include small quantities of all intermediates. Further, we note that our aim has not been to attempt to stabilize either MDA^2+^ or MDA^+^ in solution, which may be achievable, for example, by the addition of excess acid, but that this is certainly an avenue for further investigation.

### FAPbI_3_-MDACl_2_ Precursor Solution Chemistry

Having identified that HMTA and NH_4_Cl are the degradation products of MDACl_2_ in the aprotic organic solvents, we now investigate if either degradation product plays an active role in improving FAPbI_3_ crystal properties. We first perform the crystal growth as described above but with the separate addition of either HMTA or NH_4_Cl. Each additive is added in the 8:1 stoichiometry expected when 3.8 mol % MDA^2+^ degrades entirely to NH_4_^+^ (5.07 mol %) and HMTA-H^+^ (0.63 mol %). FAPbI_3_ crystals grown with NH_4_Cl additive alone undergo α-phase degradation at a rate comparable to FAPbI_3_ single crystals grown without additives. Strikingly, however, FAPbI_3_ crystals grown in the presence of HMTA (FAPbI_3_-H) show neither surface degradation nor bulk degradation to the δ-phase (Figure 1e). We quantify the stability of FAPbI_3_-H using Raman spectroscopy, as we report above for FAPbI_3_ and FAPbI_3_-M crystals, and find that, after 132 days of ambient air aging, FAPbI_3_-H crystals show no evidence (0 counts, background subtracted) of δ-phase formation (Figure 1f).

**Figure 3:**
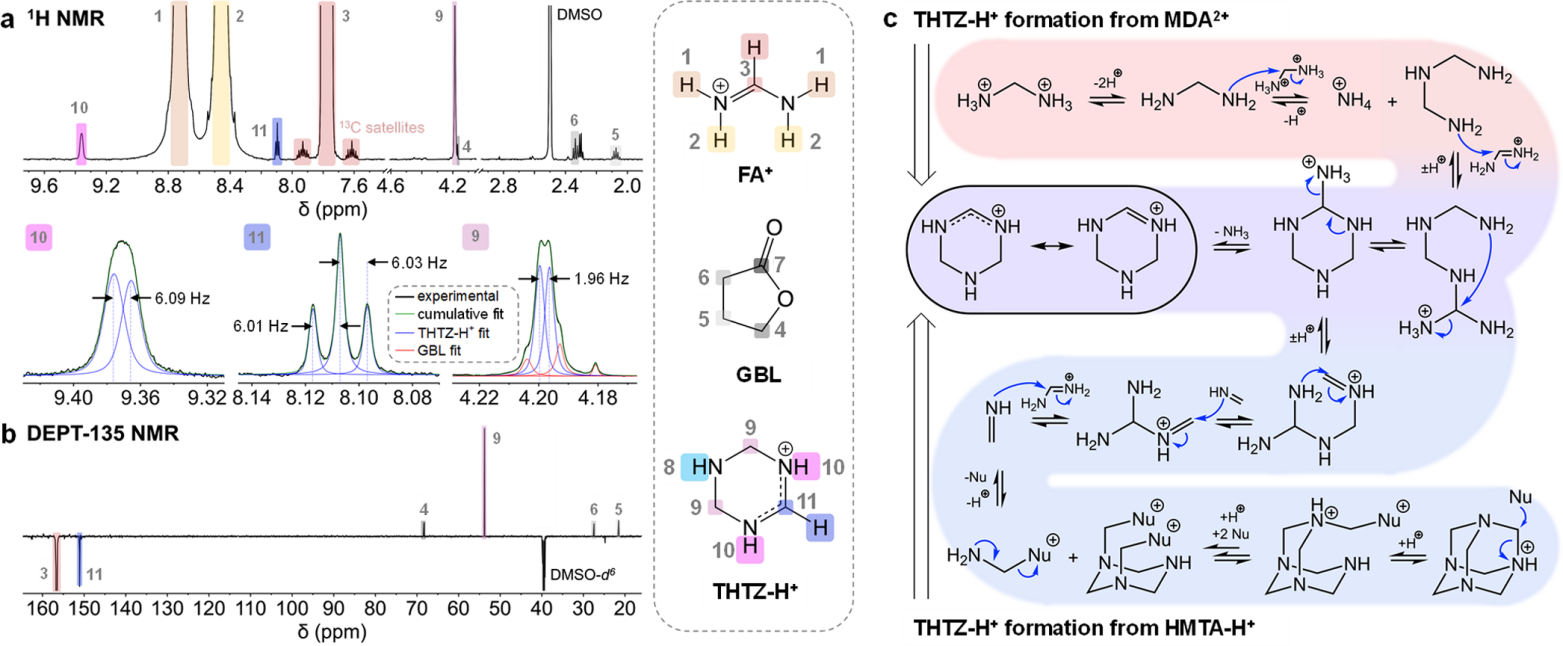
FAPbI_3_-M single-crystal composition. ^1^H solution (a) and directionless enhancement by polarization transfer (^1^H–^13^C) (DEPT-135) (b) NMR spectra (600 MHz) of FAPbI_3_-M single crystal dissolved in DMSO-*d*^6^. Spectra are referenced to the DMSO signal. Insets below ^1^H spectra show expansions of signals corresponding to THTZ-H^+^ with signal fitting and spin–spin coupling constants displayed in Hz. (c) Proposed mechanism for the formation of THTZ-H^+^ from MDA^2+^ (top left) or HMTA-H^+^ (bottom right) via generation of 2 equiv of methanimine and reaction with FA^+^. Nu corresponds to any available nucleophile in solution, most likely the HMTA additive. Further discussion is presented in Supporting Note 6.

Having established that replacing MDACl_2_ addition with a corresponding quantity of HMTA mimics the α-phase stability enhancement of FAPbI_3_-M, we conduct liquid-state ^1^H and directionless enhancement by polarization transfer (DEPT-135, ^1^H–^13^C) NMR spectroscopy (Figure 3a,b, respectively) on solutions of FAPbI_3_-M crystals dissolved in DMSO-*d*_6_. Unexpectedly, we do not detect the presence of HMTA-H^+^. Instead, in the ^1^H spectrum, we observe signals at 9.36 (d, 6.09 Hz), 8.10 (t, 6.02 Hz), and 4.19 (d, 1.96 Hz) ppm in 2:1:4 stoichiometry. Analysis of the fine structure of each of these signals suggests spin–spin coupling between nuclei giving rise to the signals at 8.10 and 9.36 ppm, with a *J*-coupling constant consistent with vicinal ^1^H–^1^H coupling trans across a sp^2^ system (^3^*J*_H9–H10_ ∼ 6 Hz). To confirm this, we carry out ^1^H–^1^H COSY (Supporting Figure S17). These data confirm the ^3^*J*_H9–H10_ coupling, while an off-diagonal cross-peak between signals at 9.36 and 4.19 ppm suggests spin–spin coupling and thus atomic proximity between these chemical environments. The DEPT-135 ^13^C spectrum shows a CH/CH_3_ signal (151.1 ppm) with a chemical shift comparable to the methine of FA^+^ (156.6 ppm) and a CH_2_ signal at 53.8 ppm, indicating an electron-depleted sp^3^ environment. These data are consistent with the presence of tetrahydro-1,3,5-triazinium (THTZ-H^+^).^33^ Integration of ^1^H methine signals in both species indicates that THTZ-H^+^ is present in solution at ∼0.5 mol % with respect to FA^+^. From these findings, it is evident that the degradation of MDA^2+^ is further complicated by the presence of other organic cations in our precursor solutions, in this instance FA^+^.

Acquisition of ^1^H NMR spectra over time upon the addition of 3.8 mol % MDACl_2_ to a solution of FAI in DMSO-*d*_6_ initially shows the formation of only HMTA-H^+^ and NH_4_^+^ (Supporting Figure S18). However, upon aging, a third product gradually evolves, THTZ-H^+^. From this, we infer that, while HMTA-H^+^ and NH_4_^+^ are the kinetic products of MDACl_2_ degradation, in the presence of FA^+^, THTZ-H^+^ is a slower-forming but thermodynamically favored product. We account mechanistically for the formation of THTZ-H^+^ from MDA^2+^ in Figure 3c (highlighted red-purple). MDA^0^-initiated cage formation (Figure 2d) is interrupted by the addition of FA^+^. Cyclization with a single FA^+^ cation leads to THTZ-H^+^.

That all steps in the reactions of Figure 3c are either substitutions or eliminations and thus mechanistically reversible, which is critical to the activity of MDA^2+^ in FAPbI_3_ crystal growth for two reasons. First, reversibility ensures that the whole reaction pathway is in dynamic equilibrium in solution. Thus, although HMTA-H^+^ formation occurs most rapidly, gradual evolution of a more favorable product by subsequent consumption of HMTA-H^+^ is possible, as emphasized in Figure 3c (highlighted blue-purple). This explains the gradual formation of THTZ-H^+^ in solution over time from a solution of MDACl_2_ and FAI (Supporting Figure S18). Second, the selective removal of any species from such a dynamic system disturbs it from equilibrium, resulting in the production of more of the species removed, in line with Chatelier’s principle.^34^ Repeated incorporation of THTZ-H^+^ into the solid state of a growing single crystal depletes its concentration remaining in solution, resulting in the production of further THTZ-H^+^. By this mechanism, ultimately, all MDA^2+^ added can be converted to THTZ-H^+^ via HMTA-H^+^ and incorporated into a growing single crystal, despite THTZ-H^+^ only ever being present in solution at very low concentration. As with all mechanistic details presented in this work, however, we emphasize that this scheme should be interpreted as a mechanistic justification only. We have not conducted extensive mechanistic studies and have only identified a relatively small number of the species displayed in the mechanistic schemes. This represents a chemically feasible route between the species we have clearly identified.

Significantly, the analysis above depends on the formation of THTZ-H^+^ by decomposition of HMTA (via protonation by weakly acidic FA^+^), as proposed in Figure 3c (highlighted blue). Thus, one test of our proposed mechanism is to confirm this is indeed the case. To do so, we obtain ^1^H NMR spectra when single crystals grown in the presence of HMTA (FAPbI_3_-H) or NH_4_Cl are dissolved in DMSO-*d*_6_ (Supporting Figures S20 and S21). In line with the mechanism presented, we find that signals corresponding to THTZ-H^+^ are observed in the spectra of all crystals grown with either HMTA or MDACl_2_ present in their precursor solution but not when NH_4_Cl alone is added. The absence of any signals in these spectra corresponding to ammonium or HMTA confirms that these additives are not included in the materials. We have therefore correlated the enhanced α-phase stability of FAPbI_3_-M and FAPbI_3_-H crystals with the presence of THTZ-H^+^ in solutions of dissolved single crystals.

### Solid-State Compositional Analysis of FAPbI_3_ Single Crystals

When FAPbI_3_-M or FAPbI_3_-H single crystals are dissolved, a solution rich in FA^+^ and with only trace quantities of THTZ-H^+^ is produced. However, as we have shown, THTZ-H^+^ formation occurs spontaneously in solutions containing FA^+^ alongside MDA^2+^, HMTA, CH_2_=NH_2_^+^, or many intermediates in the decomposition of these. Therefore, the observation of THTZ-H^+^ in solutions of dissolved crystals does not confirm that this species is present in the crystals in the solid state. Kinetic entrapment of another intermediate species during crystal growth, e.g., CH_2_=NH_2_^+^, might be expected to produce the same solution once dissolved. Nor do our liquid-state experiments provide any evidence of how a new organic species might be incorporated into the perovskite material structurally. Moreover, density functional theory (DFT) calculations assign a steric radius of 2.65 Å for THTZ-H^+^ (details of our calculations are given in Supporting Note 7). This value is only slightly larger than those for dimethylammonium (DMA^+^, 2.43 Å), ethylammonium (EA^+^, 2.42 Å), and guanidinium (GUA^+^, 2.40 Å). These three cations have all been reported as forming metastable mixed-cation 3D perovskite phases with FA^+^.^35−37^ As discussed in Supporting Note 7, these data alone do not allow us to conclude whether THTZ-H^+^ incorporates the 3D APbI_3_ perovskite A-site. Therefore, to better investigate the composition of the crystals, we conduct a range of solid-state NMR (ssNMR) measurements.

**Figure 4:**
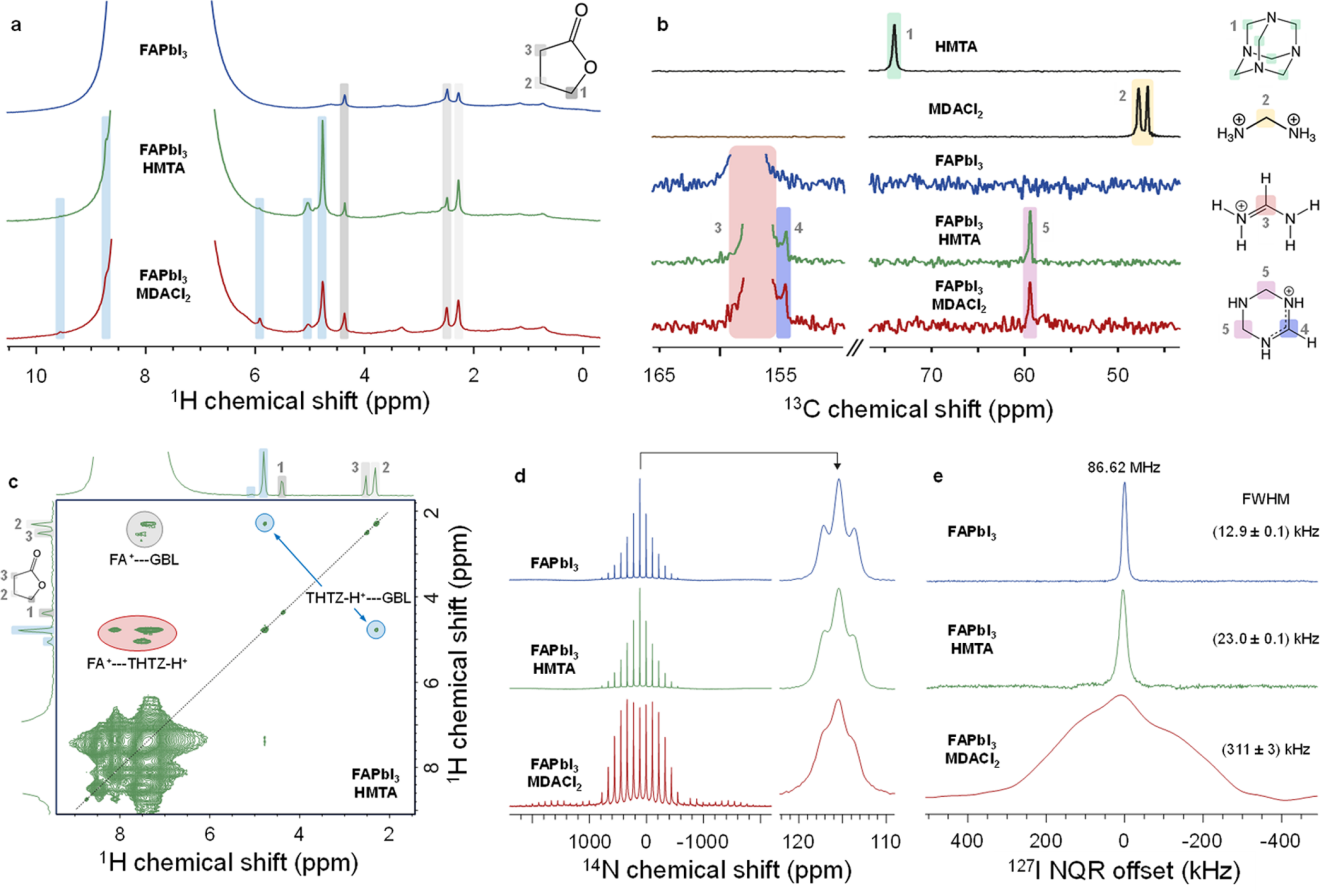
Solid-state characterization of the FAPbI_3_ single crystal. (a) ^1^H MAS (50 kHz) NMR spectra of neat FAPbI_3_, FAPbI_3_-H, and FAPbI_3_-M crystals. Signals highlighted in blue cannot be unambiguously assigned. (b) ^13^C echo-detected (12 kHz MAS) NMR spectra comparing different FAPbI_3_ crystals with additives employed in their growth. (c) ^1^H–^1^H (50 kHz MAS) spin-diffusion spectrum of FAPbI_3_-H. (d) ^14^N (4 kHz MAS) NMR spectra of FAPbI_3_, FAPbI_3_-H, and FAPbI_3_-M crystals (left). The central signal of the spectral envelope is shown in the inset to the right. (e) ^127^I NQR spectra of FAPbI_3_, FAPbI_3_-H, and FAPbI_3_-M crystals. The color schemes correlate detected signals with chemical environments of relevant nuclei in detected species.

In Figure 4a, we show ^1^H magic angle spinning (MAS) NMR spectra of FAPbI_3_, FAPbI_3_-M, and FAPbI_3_-H crystals. While the spectra are dominated by intense FA^+^ signals (6–9 ppm), several additional, well-resolved signals (highlighted in blue) are present in both FAPbI_3_-M and FAPbI_3_-H spectra that are not present in neat FAPbI_3_ crystals. These signals correspond to ^1^H environments in organic species other than FA^+^. Although it is not possible to assign these signals based on the ^1^H spectra alone, we note their close correspondence to those observed in liquid ^1^H NMR of the same crystals (Figure 3a). Further, we observe clear evidence of GBL in all three crystals, suggesting that small quantities of the processing solvent are entrapped within the crystals, despite prolonged vacuum drying (110 °C, overnight).^11,38^ By recording quantitative ^1^H solid-state NMR spectra, and assuming the same number of hydrogen nuclei in FA^+^ and the additive, we estimate that the new species is present at ∼0.5 mol % in both FAPbI_3_-M and FAPbI_3_-H crystals (see Supporting Figure S24 for the integrated regions).

To confirm that the additive detected in the solid state is indeed THTZ-H^+^, we perform ^13^C MAS NMR (Figure 4b). As in the case of ^1^H, we observe new signals (154.5, 59.4 ppm) in FAPbI_3_-M and FAPbI_3_-H, which are identical in both materials, but are absent in reference FAPbI_3_. This result corroborates that the same species is present in FAPbI_3_-M and FAPbI_3_-H despite their differing growth environments. The new signals do not correspond to MDACl_2_ (46.8, 47.7 ppm), HMTA (74.0 ppm), or δ-FAPbI_3_ (157.3 ppm, Supporting Figure S25). Instead, they closely match those expected for THTZ-H^+^ as observed via DEPT-135 in solution (151.1, 53.8 ppm) (Figure 3b).

Having detected the presence of THTZ-H^+^ in the single crystals, we next seek to elucidate its mode of incorporation within the perovskite material. This question is important since THTZ-H^+^ may be too large to replace FA^+^ on the A-site, and therefore, how it might interact with the ABX_3_ material is unclear. We first perform a ^1^H–^1^H spin-diffusion (SD) experiment, which relies on the exchange of magnetization between dipolar-coupled protons, which necessarily are in atomic-level contact on the order of tens of Å.^39^ SD therefore indicates whether the different local ^1^H environments are present within the same phase, which is the prerequisite for their being dipolar coupled.^39^Figure 4c shows the ^1^H–^1^H SD spectrum of FAPbI_3_-H. We observe intense cross-peaks between FA^+^ and THTZ-H^+^, as well as between GBL and both cations. Thus, we infer that a mixed phase containing THTZ-H^+^ and FA^+^ exists in FAPbI_3_-H and FAPbI_3_-M rather than an isolated THTZ-H^+^ secondary phase. Further discussion of the SD experiments regarding structural models of THTZ-H^+^ incorporation is given in Supporting Note 8.

We next use ^14^N MAS NMR to establish if the stabilization protocol leads to any detectable change to the local structure of the FA^+^ cations. It has previously been shown that ^14^N MAS NMR can be employed as a sensitive technique to probe perovskite lattice distortions from the perspective of A-site cation dynamics.^37,40,41^^14^N is a quadrupolar (*I* = 1) nucleus, and its NMR lineshape is determined by the symmetry of the local environment. In cubic α-FAPbI_3_, FA^+^ rapidly reorients on a ps timescale, giving a near-isotropic electric field around the ^14^N nuclei. The interaction between the small residual electric field gradient (EFG) at the ^14^N nucleus and the electric quadrupole moment (eQ) of the ^14^N nuclear spin leads to a relatively narrow FA^+^ signal linewidth and a narrow envelope of spinning sidebands.^42^ Incorporation of additive ions in the ABX_3_ lattice or distortions in the cuboctahedral haloplumbate structure de-symmetrize the A-site, leading to increased anisotropy in FA^+^ dynamics, an increased EFG at the FA^+ 14^N nuclei and a broadening of the spectral envelope.^37,40,41^^14^N MAS NMR reveals a pronounced broadening of the spectral envelope of FAPbI_3_-M, which is not observed in the spectra of either FAPbI_3_-H or neat FAPbI_3_ (Figure 4d). This implies the incorporation of a new species into FAPbI_3_-M crystals that is not present under the growth conditions of FAPbI_3_-H or neat FAPbI_3_. Considering the consistency between the ^13^C and ^1^H ssNMR spectra and the comparable quantities of THTZ-H^+^ detected in FAPbI_3_-M and FAPbI_3_-H crystals, we attribute this broadening to the presence of chloride in FAPbI_3_-M crystals. To confirm and quantify the presence of chloride, we perform electron probe microanalysis (EPMA) on FAPbI_3_-M and FAPbI_3_ crystals. These measurements show that chloride makes up ∼0.8 atom % of total halide content inside the bulk of FAPbI_3_-M (Supporting Note 9).

Incorporation of THTZ-H^+^ into the α-FAPbI_3_ phase might be expected to also result in changes in ^14^N MAS NMR. However, we detect no significant difference between FAPbI_3_-H and reference FAPbI_3_, where spectral broadening due to chloride incorporation is absent. The absence in the spectra of signals corresponding to ^14^N in incorporated THTZ-H^+^ suggests that these signals are substantially broadened with intensity spread across a large number of spinning sidebands resulting in their not being detectable. This result suggests that the EFGs at the ^14^N nuclei of THTZ-H^+^ are relatively large, most likely due to incorporated THTZ-H^+^ being static, consistent with its strong coordination to the ABX_3_ structure.

To better isolate the independent effects of THTZ-H^+^ and chloride incorporation, we conduct ^127^I nuclear quadrupole resonance (NQR) spectroscopy on the three materials (Figure 4e). NQR measurements are carried out without an external magnetic field and are a direct measure of the strength of the local EFG. Because the electric quadrupole moment of ^127^I is remarkably large (−71 Q fm^–2^, compared to the value for ^14^N: 2 Q fm^–2^), small structural changes lead to large changes to the NQR frequency. Interrogation of the full-width half-maxima (FWHM) of the NQR transition at 86.62 MHz shows resolvable differences between all three FAPbI_3_ materials. We attribute the doubling of the FWHM in FAPbI_3_-H relative to FAPbI_3_ to the incorporation of THTZ-H^+^ into the perovskite structure, which leads to the emergence of a distribution of ^127^I local environments (static disorder) throughout the bulk. This would not be the case if THTZ-H^+^ were merely adsorbed on the surface of the crystal or kinetically trapped in a pocket of entrapped precursor solution during crystallization (as is the case during agglomeration and formation of crystal inclusions).^43^ The formation of a solid solution, whereby THTZ-H^+^ is distributed homogeneously throughout the bulk, is therefore the only scenario that agrees with the experimental ^1^H–^1^H spin-diffusion and ^127^I NQR data (discussed further in Supporting Note 8). As our steric radius calculations (Supporting Note 7) indicate a substantially larger radius for THTZ-H^+^ (2.65 Å) than even FA^+^ (2.24 Å), we preclude interstitial THTZ-H^+^ as the mode of incorporation, although neither our NMR nor NQR measurements are capable of confirming this explicitly. We therefore propose a substitutional solid solution whereby one or more ions in the α-FAPbI_3_ structure are replaced by THTZ-H^+^, consistent with THTZ-H^+^ being static and strongly coordinated within the ABX_3_ structure, as inferred from our ^14^N NMR. The same ^127^I NQR transition (86.62 MHz) in FAPbI_3_-M crystals is broadened by approximately an order of magnitude more than FAPbI_3_-H, in line with additional disordering induced by the incorporation of chloride in addition to THTZ-H^+^. Previous work assessing the impact of bromide substitution in α-FAPbI_3_ on ^127^I NQR broadening suggests approximately a 1% halide substitution, consistent with our EPMA results.^44^

## Conclusions

Precisely determining the composition and structure of complex materials is often crucial, yet highly involved. Here, we have demonstrated that growth of α-FAPbI_3_ single crystals in the presence of MDACl_2_, a high-performance additive, leads to significantly reduced trap density and improved ambient α-phase stability. However, by systematically studying the solution growth conditions, we find that MDA^2+^ degrades rapidly in solution and thus cannot be incorporated into the α-FAPbI_3_ material, in contrast to previous assumptions. Instead, we show that HMTA-H^+^ and NH_4_^+^ are generated in solution as the majority degradation products. From this informed position, we propose and demonstrate an evolved form of this highly phase-stable material, FAPbI_3_-H. However, utilizing a multidisciplinary suite of characterization techniques, we find that neither FAPbI_3_-M nor FAPbI_3_-H crystals show evidence of incorporation of MDA^2+^, HMTA-H^+^, NH_4_^+^, or any other intermediates in the degradation of MDA^2+^. Instead, THTZ-H^+^ is detected in these crystals. We also discover that chloride is present in FAPbI_3_-M crystals. However, the comparable α-phase stability of our FAPbI_3_-H material allows us to isolate that it is THTZ-H^+^, not chloride, that leads to the improved stability. We rationalize the formation of THTZ-H^+^ mechanistically and perform experiments confirming that this new cation is distributed homogeneously throughout the single crystals. Using a combination of ^1^H–^1^H spin-diffusion solid-state NMR and ^127^I nuclear quadrupole resonance spectroscopy, we determine that the THTZ-H^+^ cations are incorporated into the perovskite structure in atomic-level contact with FA^+^ and leading to an appreciable distortion of the cuboctahedral symmetry. Our work will have direct consequences for the future development of high-efficiency and high-stability perovskite photovoltaic devices.

## Data Availability

All relevant data are provided in the figures, table, and Supplementary Information. The raw NMR, SCXRD, PV-SCLC, and Raman data as well as input files of the DFT calculations and optimized geometries are available on Oxford University Research Archive.

## Supporting Information Available

Experimental section: materials and synthesis, X-ray diffraction, electrical characterization, photodetectors, Raman spectroscopy, NMR spectroscopy, first principles calculations, electron probe microanalysis (PDF)
