# Supplementary material for: Understanding the Degradation of Methylenediammonium and Its Role in Phase-Stabilizing Formamidinium Lead Triiodide
Source: J Am Chem Soc. 2023 Apr 28;145(18):10275–84. doi: 10.1021/jacs.3c01531 (PMC10176466; doi:10.1021/jacs.3c01531)
Supplement: Supplementary file 1 — ja3c01531_si_001.pdf [file ja3c01531_si_001.pdf]

Supplementary Information

Understanding the degradation of methylenediammonium and its role in phase-stabilising formamidinium lead triiodide

Elisabeth A. Duijnstee[1,‡], Benjamin M. Gallant[1,‡], Philippe Holzhey[1,‡], Dominik J. Kubicki[2], Silvia Collavini[3,4], Bernd K. Sturdza[1], Harry C. Sansom[1], Joel Smith[1], Matthias J. Gutmann[5], Santanu Saha[1], Murali Gedda[6], Mohamad I. Nugraha[6,7], Manuel Kober-Czerny[1], Chelsea Xia[1], Adam D. Wright[1], Yen-Hung Lin[1], Alexandra J. Ramadan[8,1], Andrew Matzen[9], Esther Y.-H. Hung[1], Seongrok Seo[1], Suer Zhou[1], Jongchul Lim[1,10], Thomas D. Anthopoulos[6], Marina R. Filip[1], Michael B. Johnston[1], Robin J. Nicholas[1], Juan Luis Delgado[3,11], Henry J. Snaith[1*]*

[1] Clarendon Laboratory, Department of Physics, University of Oxford, Parks Road, Oxford, OX1 3PU, United Kingdom

[2] Department of Physics, University of Warwick, Coventry, CV4 7AL, United Kingdom

[3] POLYMAT, University of the Basque Country UPV/EHU, Avenida de Tolosa 72 & Faculty of Chemistry, P. Manuel Lardizabal 3, 20018 Donostia-San Sebastián, Spain

[4] Faculty of Chemistry, P. Manuel Lardizabal 3, 20018 Donostia-San Sebastián, Spain

[5] ISIS Facility, STFC Rutherford Appleton Laboratory, Harwell Science and Innovation Campus, Chilton, Didcot, Oxfordshire OX11 0QX, United Kingdom

[6] King Abdullah University of Science and Technology (KAUST), KAUST Solar Center, Thuwal 23955, Saudi Arabia

[7] Research Center for Advanced Materials, National Research and Innovation Agency (BRIN), South Tangerang, Banten 15314, Indonesia

[8] Department of Physics and Astronomy, The University of Sheffield, Hicks Building, Hounsfield Road, Sheffield S3 7RH, UK.

[9] Department of Earth Sciences, University of Oxford, 3 South Parks Road, Oxford, OX1 3AN, United Kingdom

[10] Graduate School of Energy Science and Technology (GEST), Chungnam National University, 99 Daehak-ro, Yuseong-gu, Daejeon, 34134, Korea

[11] Ikerbasque, Basque Foundation for Science, 48013 Bilbao, Spain

*Henry J. Snaith: henry.snaith@physics.ox.ac.uk

*Juan Luis Delago: juanluis.delgado@polymat.eu

Table of contents

Section S1. Materials and Synthesis

Section S2: Single crystal X-ray diffraction

Section S3. Electrical characterisation

Section S4. Photodetectors

Section S5. Raman spectroscopy

Section S6. Liquid-state Nuclear Magnetic Resonance spectroscopy

Section S7. First Principles Calculations of Cation Steric Radii.

Section S8. Solid-state Nuclear Magnetic Resonance spectroscopy

Section S9: Electron Probe Microanalysis

Section S10. References

Section S1: Materials and Synthesis

Materials

y-butyrolactone (GBL, Alfa Aesar), formamidinium iodide ($HC(NH_2)_2I$, Greatcell Solar), methylenediammonium dichloride ($H_2C(NH_3)_2Cl_2$, Sigma Aldrich), hexamethylenetetramine (HMTA, Sigma Aldrich), ammonium chloride (NH_4Cl, Sigma Aldrich), dimethyl sulfoxide-d^6 (DMSO-d^6, Sigma Aldrich), lead iodide (PbI_2, TCI). All chemicals were used as received and without any further purification.

Synthesis of Single Crystals

All single crystals were fabricated in N_2 from a seed crystal by following a previously published experimental protocol[1,2]. The control $FAPbI_3$ single crystals are prepared by dissolving equimolar FAI and PbI_2 in γ-butyrolactone (GBL). The solution is then stirred at 60 °C for four hours. Then the solution is filtered with a 25 mm diameter 0.45 µm GMF filter. 4 ml of the filtrate is then placed in a vial that contains a seed $FAPbI_3$ crystal, and the vial is kept in an oil bath undisturbed at 95 °C for 12 hours. The crystals were then dried in a vacuum oven at 180 °C for 45 minutes. For the $FAPbI_3$-M and $FAPbI_3$-H single crystals, alongside equimolar FAI and PbI_2 either 3.8 mol% $MDACl_2$ or 0.63 mol% HMTA is added. Then the same procedure as for the reference $FAPbI_3$ crystals was followed.

For SCLC measurements: To control the thickness, a small chamber was constructed using two thin glass plates of different thicknesses placed on the bottom of the glass vial with a gap in between and a cover glass on top. Free-standing, millimetre-sized crystals were removed from the vial once formed. A schematic of the single crystal growth and device fabrication is depicted in **Supplementary Figure S1**.

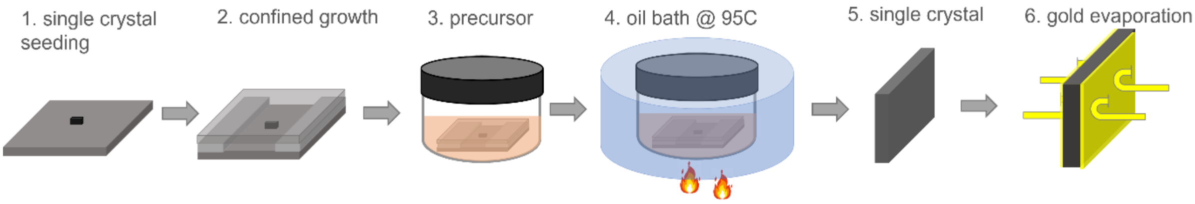

Figure S1: Schematic illustration of the single crystal synthesis and device fabrication process: 1. Single crystal seeding; 2. Seed crystal in confined growth template; 3. Addition of precursor solution; 4. Crystal growth in oil bath at 95 °C; 5. Free-standing single crystal; 6. Gold evaporation and gold wire attachment for electrical characterization measurements.

Section S2: Single crystal X-ray diffraction (SCXRD)

Single crystal X-ray diffraction (SCXRD) was collected on a Rigaku Oxford Diffraction XTaLAB Synergy diffractometer with Mo Kα (λ = 0.71073) wavelength, located in the laboratories at the ISIS Neutron and Muon Source, Oxfordshire, U.K. Crystal data was indexed and reduced using CrysalisPro and the structures solved using ShelXL implemented through Olex2. Before measuring the crystals, they were heated to 453 K (180 °C) in an N_2 stream for 2.5 minutes to convert any δ-$FAPbI_3$ decomposition products that may have formed during storage back in to the perovskite α-phase. They were then cooled back down to 290 K for data collection.

SCXRD confirms the perovskite α-phase in each of the $FAPbI_3$, $FAPbI_3$-M and $FAPbI_3$-H crystals. The crystal structures were solved in the $Pm\bar{3}m$ cubic space group as previously reported[3], with crystal data and structure refinement statistics shown in **Table S1**. The solved crystal structure of the $FAPbI_3$ crystal is shown in **Figure S2**, which is representative of all three crystals, with a near-spherical distribution for the organic cation on the A-site, as expected from its rotational disorder. $FAPbI_3$-M and $FAPbI_3$-H show slightly longer average Pb-I bond lengths (and larger unit cells) and slightly lower atomic displacement parameters for Pb compared to $FAPbI_3$, shown in **Tables S2 and S3**. It is difficult to assess whether these differences are significant, with the average bulk structures attained from the SCXRD less sensitive to small compositional substitutions or local distortions than, for example, our solid-state nuclear magnetic resonance (ssNMR) and electron probe microanalysis (EPMA) measurements, highlighting the need for advanced characterisation techniques to gain more complete understanding of halide perovskites.

What is more apparent from our SCXRD measurements, however, is the difference in the amount of twinning in the crystals (**Figure S3**). Without background subtraction, we can see the presence of weakly diffracting powder rings in the diffraction pattern for $FAPbI_3$ which show the presence of many randomly orientated crystallographic domains. The polycrystalline-like diffraction rings are readily removed by the background removal, making the resulting diffraction pattern of $FAPbI_3$ crystals falsely appear to be a perfectly single crystal domain. We observe a greatly reduced presence of Debye-Scherrer rings in $FAPbI_3$-M and $FAPbI_3$-H crystals, particularly for the $FAPbI_3$-M crystal.

After background subtraction, 100% of the diffraction spots obtained for the $FAPbI_3$ crystal could be indexed to a single cubic unit cell. For $FAPbI_3$-M, 77% of diffraction spots could be fitted to a single

cubic unit cell, increasing to 87% and 96% by the addition of two more twin components. The main component was detwinned and used to solve the structure. In the case of $FAPbI_3$-H, only 47% of diffraction spots could be fitted with a single cubic unit cell. This could increase to 57% and then 66% by adding two more twin components. Fitting a fourth twin component only indexed 2% more of the diffraction spots, and so we stopped at three twin components. The remaining diffraction spots are likely to be due to the presence of some randomly orientated grains. The main component attributed to 47% of the diffraction spots was detwinned and used to solve the crystal structure. The twin laws and overlap statistics for $FAPbI_3$-M and $FAPbI_3$-H are shown below. In all cases. there is little overlap between twins. The twinning in FAPI-M and FAPI-H may be similar; both crystals have a twin component rotated ~174° from the main component, and in both FAPI-M and FAPI-H the second and third twin components are related by a ~120° rotation.

In general, many of the crystals grown and measured in this work appear to be single crystals. However, for metal halide perovskites there have been several reports showing that both thin film grains and "single" crystals can contain several distinct crystallographic domains[4–6]. It is unclear if there is a link between the density of twinning in our crystals and the enhanced α-phase stability we observe. It has been suggested that point-defects can be the origin of domain boundaries due to memory effects seen when heating and cooling domain boundaries through phase transitions[5,6]. Comparing the diffraction spots observed for each crystal shows that our more stable crystals ($FAPbI_3$-M and $FAPbI_3$-H) also show a significantly greater density of well-defined twins, rather than the randomly orientated crystallites seen in $FAPbI_3$. It is possible that the inclusion mode of THTZ-H^+ is such to have caused this effect, perhaps by inducing point defects that in turn induce distinct twin domains that are less likely to lead to propagation of the photoinactive δ-phase. Equally, however, this may be a confusion of cause and effect; the observation of many more randomly ordered domains in the $FAPbI_3$ crystals may instead be early evidence of the onset of phase degradation, especially as it is not straightforward to assess if the majority of these disordered domains correspond to crystallites in the α- or δ-phase. We emphasise that all our SCXRD measurements were performed on crystals that were freshly grown (<1 day old, in the case of $FAPbI_3$) and had been stored in a static N_2 environment from time of fabrication to time of measurement. As above, the measurements themselves were performed under a flow of temperature-controlled N_2. All these precautions were taken in order to minimise degradation of the as-fabricated crystals to the δ-phase prior to measurement, nonetheless we cannot eliminate this possibility.

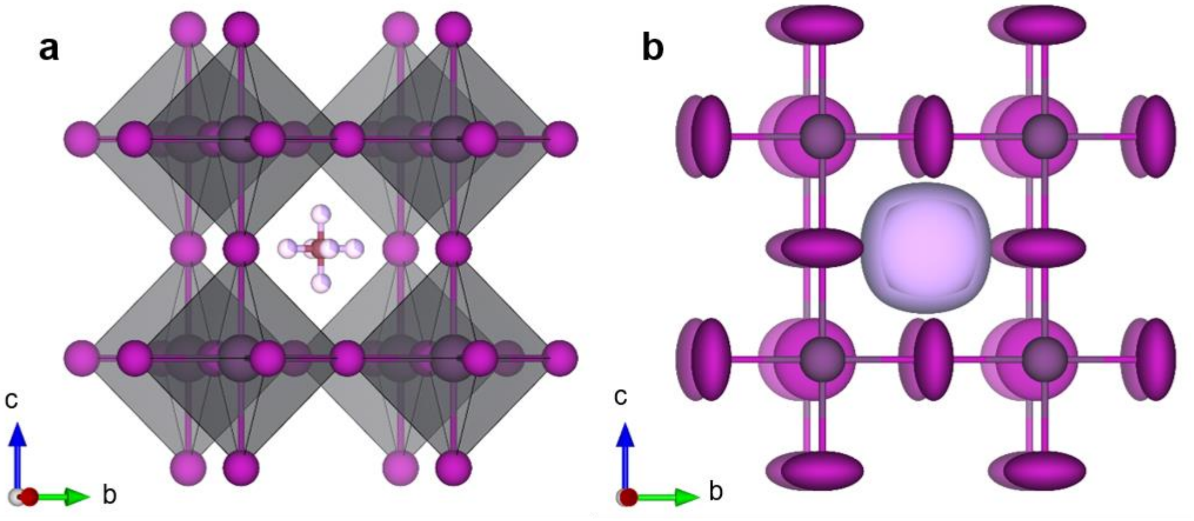

Figure S2. (a) The crystal structure solved for $FAPbI_3$ crystals in the cubic Pm$\bar{3}$m space group, as reported. (b) The crystal structure of $FAPbI_3$ crystals, shown with 100% displacement parameters showing the near-spherical electron distribution of the organic cation. The frontmost plane of I^- ions have been removed to show the displacement parameters of the Pb^{2+}. These images are also representative of the $FAPbI_3$-M and $FAPbI_3$-H crystals.

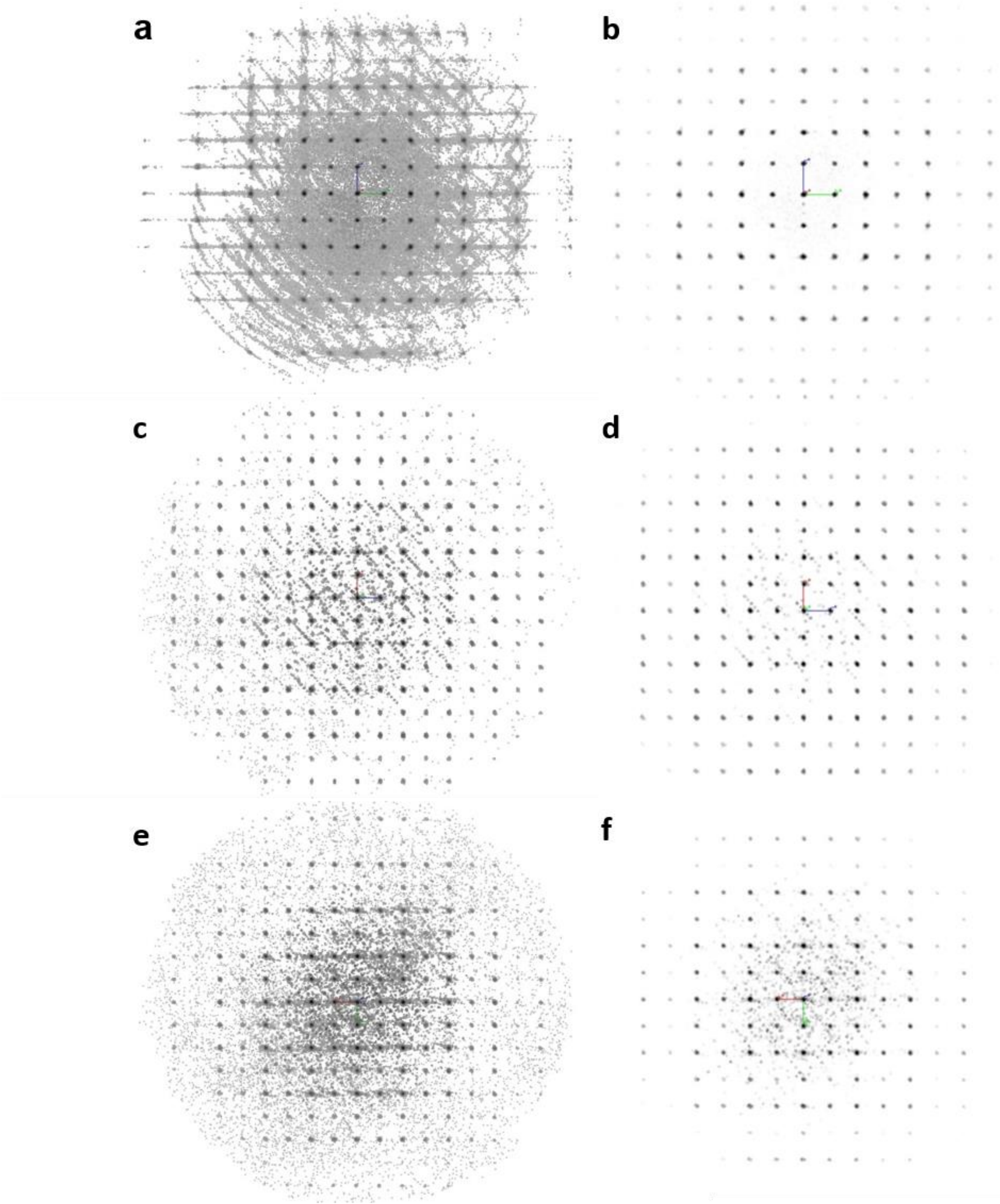

Figure S3. The sphere of diffraction spots collected for $FAPbI_3$ (a), $FAPbI_3$-M (c) and $FAPbI_3$-H (e) crystals without background subtraction. The sphere of diffraction spots collected for $FAPbI_3$ (b), $FAPbI_3$-M (d) and $FAPbI_3$-H (f) crystals with background subtraction. Views are projected in the (0kl) plane.

Table S1. Crystal data and structure refinement for the $FAPbI_3$, $FAPbI_3$-M and $FAPbI_3$-H crystals.

**Identification code**	$FAPbI_3$	$FAPbI_3$-M	$FAPbI_3$-H
**Empirical formula**	$(C_2H_5N_2)PbI_3$	$(C_2H_5N_2)PbI_3$	$(C_2H_5N_2)PbI_3$
**Formula weight**	627.92	627.92	627.92
**Temperature/K**	289.99(10)	289.99(10)	290.0(4)
**Crystal system**	cubic	cubic	cubic
**Space group**	$Pm\bar{3}m$	$Pm\bar{3}m$	$Pm\bar{3}m$
**a/Å**	6.35253(12)	6.35469(4)	6.35530(19)
**b/Å**	6.35253(12)	6.35469(4)	6.35530(19)
**c/Å**	6.35253(12)	6.35469(4)	6.35530(19)
**α/°**	90	90	90
**β/°**	90	90	90
**γ/°**	90	90	90
**Volume/Å^3**	256.354(15)	256.616(5)	256.690(12)
**Z**	1	1	1
**ρ_{calc}g/cm^3**	4.067	4.063	4.062
**μ/mm^{-1}**	25.41	25.384	25.377
**F(000)**	261	261	261
**Crystal size/mm^3**	0.023 × 0.039 × 0.061	0.035 × 0.065 × 0.107	0.025 × 0.066 × 0.079
**Radiation**	Mo Kα (λ = 0.71073)	Mo Kα (λ = 0.71073)	Mo Kα (λ = 0.71073)
**2Θ range for data collection/°**	6.414 to 64.504	6.412 to 64.478	6.41 to 64.4728
**Index ranges**	$-9 \le h \le 9$, $-9 \le k \le 9$, $-9 \le l \le 9$	$-9 \le h \le 9$, $-9 \le k \le 9$, $-9 \le l \le 9$	$-9 \le h \le 9$, $-9 \le k \le 9$, $-9 \le l \le 9$
**Reflections collected**	6054	6500	6568
**Independent reflections**	128 [R_{int} = 0.0653, R_{sigma} = 0.0131]	127 [R_{int} = 0.0345, R_{sigma} = 0.0060]	128 [R_{int} = 0.0587, R_{sigma} = 0.0131]
**Data/restraints/parameters**	128/0/9	127/0/9	128/0/9
**Goodness-of-fit on F^2**	1.099	1.113	1.291
**Final R indexes [I>=2σ (I)]**	R_1 = 0.0146, wR_2 = 0.0333	R_1 = 0.0100, wR_2 = 0.0285	R_1 = 0.0207, wR_2 = 0.0503
**Final R indexes [all data]**	R_1 = 0.0177, wR_2 = 0.0335	R_1 = 0.0100, wR_2 = 0.0285	R_1 = 0.0207, wR_2 = 0.0503
**Largest diff. peak/hole / e Å^{-3}**	0.58/-0.41	0.50/-0.40	1.17/-0.36

Table S2. Anisotropic displacement parameters ($Å^2 \times 10^3$) of the solved crystal structures for the $FAPbI_3$, $FAPbI_3$-M and $FAPbI_3$-H crystals. The anisotropic displacement factor exponent takes the form: $-2\pi^2[h^2a^{*2}U_{11}+2hka^*b^*U_{12}+\ldots]$.

Atom	U_{11}	U_{22}	U_{33}	U_{23}	U_{13}	U_{12}
			**$FAPbI_3$**			
Pb1	38.36(18)	38.36(18)	38.36(18)	0	0	0
I1	28.8(3)	123.5(5)	123.5(5)	0	0	0
C1	151(16)	151(16)	151(16)	0	0	0
N1	220(20)	68(19)	220(20)	0	0	0
			**$FAPbI_3$-M**			
Pb1	35.73(14)	35.73(14)	35.73(14)	0	0	0
I1	121.7(4)(2)	27.3(2)	121.7(4)	0	0	0
C1	141(14)	141(14)	141(14)	0	0	0
N1	210(20)	74(17)	210(20)	0	0	0
			**$FAPbI_3$-H**			
Pb1	36.5(2)	36.5(2)	36.5(2)	0	0	0
I1	122.2(7)	122.2(7)	28.7(4)	0	0	0
C1	140(20)	140(20)	140(20)	0	0	0
N1	190(30)	70(20)	190(30)	0	0	0

Table S3. Bond lengths of the solved crystal structures for the FAPI, FAPI-M and FAPI-H crystals.

Atom	Atom	Length/Å
	**$FAPbI_3$**	
Pb1	I1	3.17627(6)
C1	N1	1.00(3)
N1	N1	1.41(4)
	**$FAPbI_3$-M**	
Pb1	I1	3.17735(2)
C1	N1	1.01(3)
N1	N1	1.42(4)
	**$FAPbI_3$-H**	
Pb1	I1	3.17765(5)
C1	N1	0.99(4)
N1	N1	1.40(6)

Twin laws and overlap statistics for detwinning in $FAPbI_3$-M crystals.

Twin law for component 1 with component 2, where UB[n] is the orientation matrix for component n:

$$UB[1]\text{-}1*UB[2] = \begin{pmatrix} 0.1315 & 0.9802 & 0.1479 \\ 0.1503 & 0.1278 & -0.9791 \\ -0.9795 & 0.1506 & -0.1297 \end{pmatrix},$$

a 115.8348° rotation around reciprocal space vector (h,k,l): (0.6288, 0.6263, -0.4608)

Twin law for component 1 with component 3:

$$UB[1]\text{-}1*UB[3] = \begin{pmatrix} 0.6011 & -0.3840 & -0.6977 \\ -0.3043 & -0.9208 & 0.2410 \\ -0.7395 & 0.2429 & -0.6724 \end{pmatrix},$$

a -174.4062 ° rotation around reciprocal space vector (h,k,l): (0.8944, -0.1925, -0.4037)

Twin law for component 2 with component 3:

$$UB[2]\text{-}1*UB[3] = \begin{pmatrix} 0.7582 & -0.2556 & 0.6038 \\ 0.4385 & -0.4839 & -0.7547 \\ 0.4844 & 0.8381 & -0.2520 \end{pmatrix},$$

a -119.2953 ° rotation around reciprocal space vector (h,k,l): (0.9149, 0.0669, 0.3981)

Overlap statistics of components 1 and 2:

Overlap factor	Number in range	Percentage of all observations
0.0-0.2	81	0.4
0.2-0.4	50	0.3
0.4-0.6	17	0.1
0.6-0.8	2	0.0
0.8-1.0	16	0.1
**Total:**	**166**	**0.9**

Overlap statistics of components 1 and 3:

Overlap factor	Number in range	Percentage of all observations
0.0-0.2	67	0.4
0.2-0.4	23	0.1
0.4-0.6	7	0.0
0.6-0.8	3	0.0
0.8-1.0	0	0.0

**Total:**	**100**	**0.5**

Overlap statistics of components 2 and 3:

Overlap factor	Number in range	Percentage of all observations
0.0-0.2	71	0.4
0.2-0.4	17	0.1
0.4-0.6	13	0.1
0.6-0.8	10	0.1
0.8-1.0	22	0.1
**Total:**	**133**	**0.7**

Twin laws and overlap statistics for detwinning in $FAPbI_3$-H crystals.

Twin law for component 1 with component 2, where UB[n] is the orientation matrix for component n:

$$UB[1]\text{-}1{*}UB[2] = \begin{pmatrix} -0.9865 & -0.1590 & -0.0172 \\ -0.0210 & 0.0306 & 1.0002 \\ -0.1580 & 0.9870 & -0.0333 \end{pmatrix},$$

a 174.3107˚ rotation around reciprocal space vector (h,k,l): (-0.0633, 0.7169, 0.6943)

Twin law for component 1 with component 3:

$$UB[1]\text{-}1{*}UB[3] = \begin{pmatrix} 0.6093 & -0.6799 & -0.4098 \\ -0.7870 & -0.5863 & -0.1968 \\ -0.1066 & 0.4427 & -0.8914 \end{pmatrix},$$

a 159.0332˚ rotation around reciprocal space vector (h,k,l): (0.8931, -0.4243, -0.1496)

Twin law for component 2 with component 3:

$$UB[2]\text{-}1{*}UB[3] = \begin{pmatrix} 0.5675 & 0.6145 & 0.5504 \\ -0.2256 & 0.5271 & -0.8204 \\ -0.7919 & 0.5894 & -0.1601 \end{pmatrix},$$

a -126.9241 ˚ rotation around reciprocal space vector (h,k,l): (0.1451, 0.8393, -0.5239)

Overlap statistics of components 1 and 2:

**Overlap factor**	**Number in range**	**Percentage of all observations**
0.0-0.2	239	1.2
0.2-0.4	52	0.3
0.4-0.6	14	0.1
0.6-0.8	5	0.0
0.8-1.0	5	0.0
**Total:**	**315**	**1.6**

Overlap statistics of components 1 and 3:

**Overlap factor**	**Number in range**	**Percentage of all observations**
0.0-0.2	27	0.1
0.2-0.4	8	0.0
0.4-0.6	6	0.0
0.6-0.8	3	0.0
0.8-1.0	1	0.0

**Total:**	**45**	**0.2**

Overlap statistics of components 2 and 3:

Overlap factor	Number in range	Percentage of all observations
0.0-0.2	46	0.2
0.2-0.4	10	0.1
0.4-0.6	12	0.1
0.6-0.8	7	0.0
0.8-1.0	7	0.0
**Total:**	**82**	**0.4**

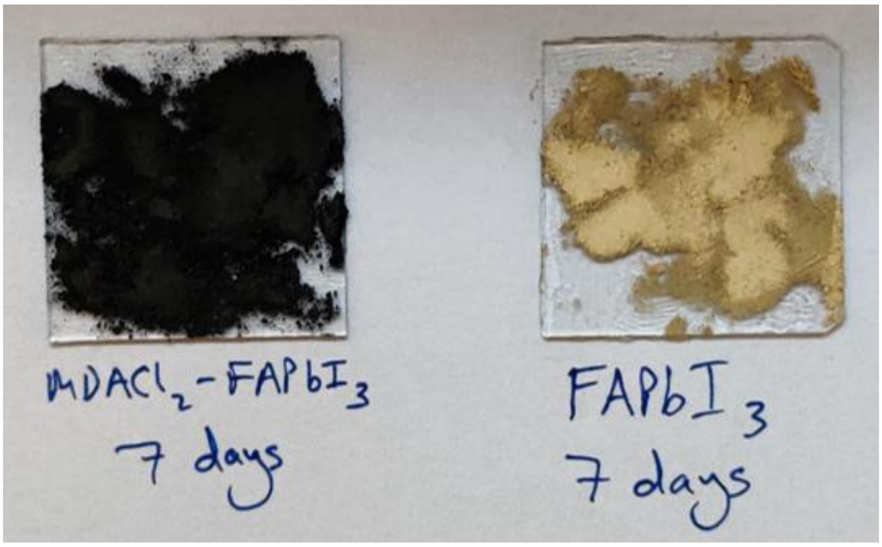

Figure S4: Powders produced when $FAPbI_3$-M (left) and $FAPbI_3$ (right) single crystals are stored in ambient conditions for seven days then ground with a pestle and mortar.

Section S3: Electrical characterisation

Pulsed-voltage space-charge limited current measurement (PV-SCLC): For electrical characterization, 120 nm gold electrodes were evaporated on both of the larger faces of the single crystal by an evaporator (Kurt J. Lesker, Nano36) at a 0.5 A s^{-1} deposition rate. The J-V traces were measured using a computer-controlled 2400 Series Keithley source meter in the dark under vacuum at room temperature. The vacuum pump (Leybold vacuum, PT 70 F-Compact) pumped the system down to 10^{-4} mbar.

Time-resolved photoluminescence (TRPL): The samples were mounted in air and photoexcited by a 398 nm picosecond pulsed diode laser (PicoHarp, LDH-D-C-405M). The resultant PL was collected and coupled into a grating spectrometer (Princeton Instruments, SP-2558), which directed the spectrally dispersed PL onto a photon-counting detector (PDM series from MPD), whose timing was controlled with a PicoHarp300 TCSPC event timer. Laser fluences of between 2 and 1500 nJ cm^{-2} were used, with a laser repetition rate of 2.5 MHz. The PL transients were collected at the PL peak wavelengths of the single crystals: 816 nm for the $FAPbI_3$ and 826 nm for the $FAPbI_3$-M.

The PL decay traces were each fitted by a stretched exponential function $I = I_0 \exp(-(t/\tau)^{\beta})$, where β is the distribution coefficient and τ is the time taken for the PL intensity to drop to I_0/e. Such stretched exponential functions have been used to phenomenologically account for the presence of a local distribution of monoexponential decay rates, whose average lifetime is given by $\tau_{av} = (\tau/\beta)\,\Gamma(1/\beta)$, where Γ is the gamma function[7,8].

Terahertz (THz) mobility: The electrical mobility of the $FAPbI_3$ and $FAPbI_3$-M single crystals were measured using optical-pump-THz-probe spectroscopy (OPTPS) in reflection geometry. The single crystals were photoexcited by a 35 fs pulsed laser with central wavelength at 400 nm, which generates free charge carriers and results in an increase of the reflected THz signal. The electrical mobility was extracted using the method detailed in Xia, et al.[9]. According to the OPTPS measurements, $FAPbI_3$ and $FAPbI_3$-M single crystals exhibit mobility values of (26.9 ± 1.5) and (23.3 ± 0.8) $cm^2V^{-1}s^{-1}$ respectively. Since the OPTPS measurement is sensitive to the surface condition of the single crystal such as surface defects and trap states, both single crystals were then cleaved and measured again to extract the bulk mobility. Consequently, the mobilities of $FAPbI_3$ and $FAPbI_3$-M single crystals drop slightly to (22.4 ± 0.5) and (20.7 ± 1.0) $cm^2V^{-1}s^{-1}$ respectively. In both cases, the $FAPbI_3$ single crystal shows a slightly higher mobility than the $FAPbI_3$-M single crystal.

Transient photo-conductivity (TPC): An Nd:YAG laser (Ekspla NT342A, 10 Hz with 3.74 ns FWHM) is coupled into an optical parametric oscillator to obtain various wavelengths in a home-built setup. The laser is attenuated by an optical density filter to reach several fluences for each wavelength (typically between 1.5 and 0.01 μJ cm^{-2}). The spot size of the pulsed light is 0.5 cm^2, which is 10x larger than the single crystals used, ensuring that they are uniformly excited. A small DC bias (< 5 mV/μm) is applied across two in-plane (lateral) gold electrodes that have been evaporated directly onto the single crystal. We monitor the voltage drop across the variable series resistor through a parallel oscilloscope (1MΩ input impedance) to determine the potential dropped across the two in-plane Au electrodes on the sample. We then extract the photoconductivity directly from the data and estimate the sum-mobility (Σμ) taking recombination and the free carrier fraction into account. The estimation error is greatly influenced by the sample thickness as diffusion in the z-direction is not taken into account in our analysis, which explains the error bars seen in **Figure S4**. For more detail see our recent work[10,11].

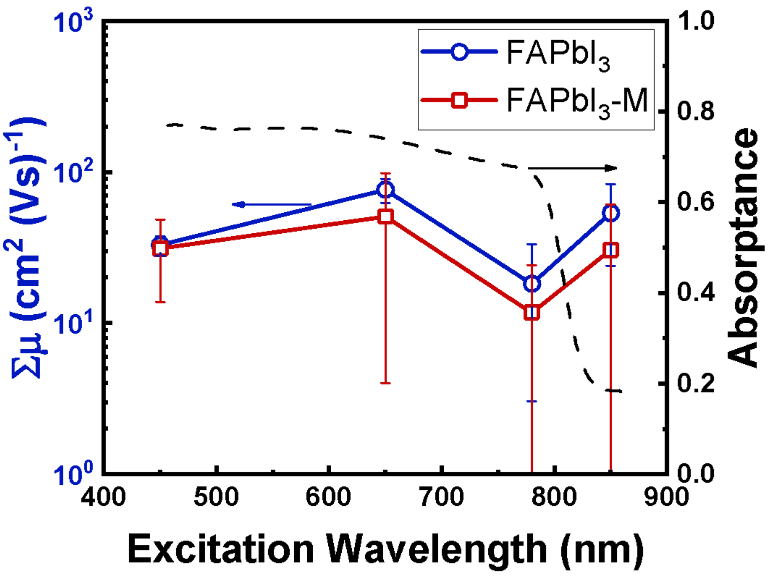

Figure S5: Evaluation of long-range mobility of perovskite single crystals by transient photoconductivity measurement. The sum of mobilities ($\sum \mu$) is shown as a function of wavelength, after taking recombination and the free carrier fraction into account. The absorption spectrum of a typical $FAPbI_3$ thin film is presented alongside the data to indicate, where the optical bandgap is located.

Section S4: Photodetectors

Single crystal planar photodetectors were fabricated by applying two Ag electrodes onto the single crystal, as shown in the schematic below. The Ag electrodes were patterned on top of each single crystal using Ag-ink. A needle of ~100 µm diameter has been used to mask the crystal surface while applying Ag-ink to produce a channel of ~100 µm. The electrical characteristics of the photodetectors were monitored by a probe station that is connected to an Agilent B1500A semiconductor parameter analyser and a continuous light source with wavelength of 780 nm.

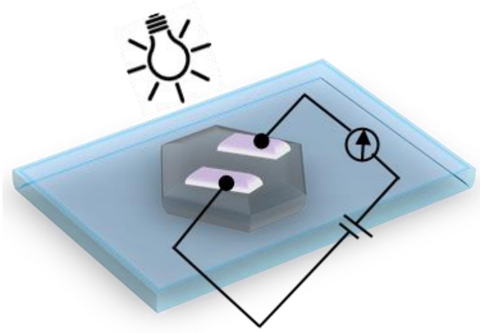

Figure S6: Schematic representation of single-crystal based planar photodiode with Ag electrodes.

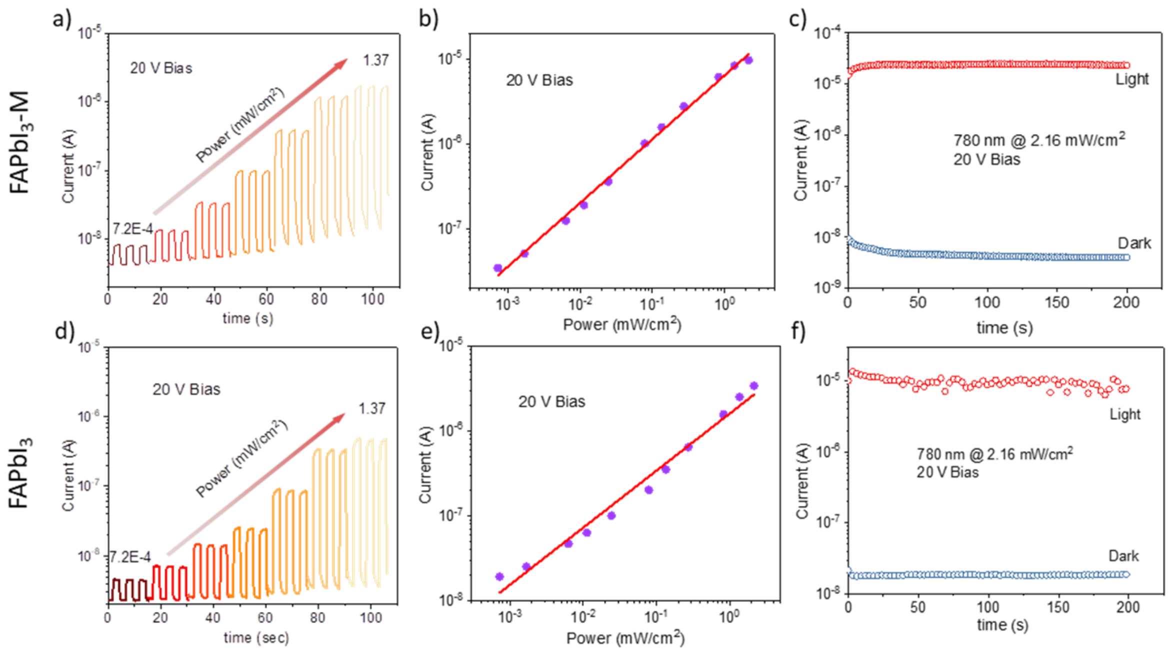

Figure S7: Photodetector performance of $FAPbI_3$-M (**a**, **b** and **c**) and $FAPbI_3$ (**d**, **e** and **f**) single crystals. **a)** and **d)** represents the variation of photocurrent switching with power density. **b)** and **e)** shows the linearity of power density-dependent photocurrent. **c)** and **f)** shows the stability of the photodetector operated at constant power and voltage bias. The time-dependent photoresponse of the planar photodiode was measured under a 780 nm monochromatic LED illumination. The measurement results (20 V voltage bias) indicated that the switching was stable, multiple, and reproducible. The photocurrent showed a logarithmic dependence on the power density and it is more linear for the FAPbI3-M single-crystal device, indicating efficient photocharge generation.

Section S5: Raman spectroscopy

Raman spectra were acquired with a Jobin Yvon T64000 triple spectrometer and an Andor DU420A-OE CCD. The spectrometer was calibrated with the 520.7 cm^{-1} line of a Silicon wafer before each measurement. Samples were excited with a Ventus solo Nd:YAG laser (λ = 532 nm) and the incident laser irradiation of the samples was strictly kept at intensities below 5 W/cm^2 to prevent laser induced degradation. This has been a major source of confusion for $FAPbI_3$ Raman mode assignment in the literature and we thus opted for longer exposure times at extremely low laser intensities.

Raman spectroscopy has been used extensively to distinguish the δ- and α-phases in $FAPbI_3$, as the different symmetries of the two phases allow identification via their Raman signatures. Han et al. were the first to report Raman signatures for both phases, assigning peaks at 111 cm^{-1} and 135 cm^{-1} to the δ- and α-phase, respectively[12]. This assignment has been used in numerous studies[13–15] since, even though the cubic Pm-3m symmetry of the α-phase is predicted to be Raman inactive by group theory. Two recent reports by Driscoll *et al.*[16] and Ibaceta-Jana *et al.*[17] dissent previous assignments and provide experimental evidence that the α-phase is Raman inactive. These reports match our observations.

Comment on selection of peak at 108 cm^{-1}:

There are four modes of the inorganic framework in the δ-phase: 35, 55, 83 and 108 cm^{-1}. Raman modes become harder to detect the closer they appear to the laser line (0 cm^{-1}) due to its linewidth. This has two consequences:

1. Most Raman spectrometers can only detect the line at 108 cm^{-1}, as they use optical filters to cut out the laser line.
2. For modes closer to the laser line, there will be more of a background issue from the tail of the laser line. This could be subtracted, but for a quantitative analysis it's more straightforward to take a line slightly further away.

For compounds containing organic entities in an inorganic framework, like $FAPbI_3$, we can separate the Raman modes of the framework from the modes of the organic part. Modes of FA are all substantially above 200 cm^{-1}; modes of the haloplumbate framework in the range <200 cm^{-1}. By looking at the 108 cm^{-1} peak, we are probing the PbI bond. However, we prefer to think about this in terms of the symmetry

of the PbI cage instead of individual bonds, as the symmetry defines whether something is Raman active or not.

We note that the single crystals analysed after aging for 1 year ($FAPbI_3$ and $FAPbI_3$-M) and 132 days ($FAPbI_3$-H) and presented in **Figure 1f** originate from the same single crystal fabrication batches as the crystals used for the 33-day-experiment (the first 33 days). However, these crystals were stored in glass vials under ambient conditions, while the crystals in the 33-day-experiment were constantly directly exposed to air. The Raman spectra of freshly annealed (α-phase) and fully degraded (δ-phase) $FAPbI_3$ single crystals are shown in **Figure S9a**. Both spectra were acquired with identical experimental settings, but the δ-phase was divided by 400. This shows that the Raman scattering cross section of α-phase $FAPbI_3$ is at least two orders of magnitude weaker than for the δ-phase[13]. The overall Raman signal of any given $FAPbI_3$ sample will consequently be dominated by δ-phase peaks as soon as a small fraction of the probed layer is degraded.

This allows us to track the degraded fraction of the probed surface layer by monitoring the δ-phase peak intensities, even if the crystal is predominantly in the α-phase. We note that peak area is always proportional to the amount of δ-phase present in the crystal and, as is the case here, if peak width is constant then peak intensity correlates directly with peak area. The Raman spectra used for the single crystal stability data in main text **Figure 1f** are shown in **Figure S9b-d**.

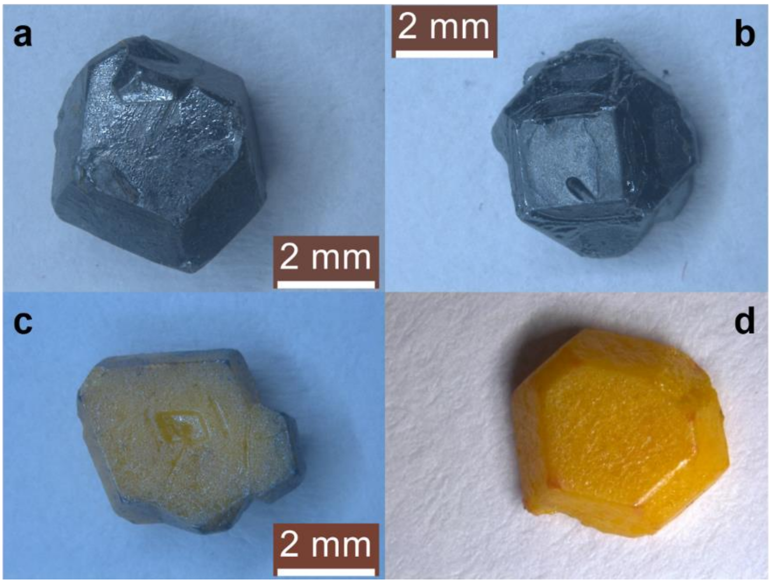

Figure S8: Aged single crystals. **(a)** $FAPbI_3$-H, **(b)** $FAPbI_3$-M, and **(c)** $FAPbI_3$ single crystals stored in sealed boxes in ambient air for 15 months. **(d)** $FAPbI_3$ single crystal fully degraded to the δ-phase in an N_2-filled glovebox.

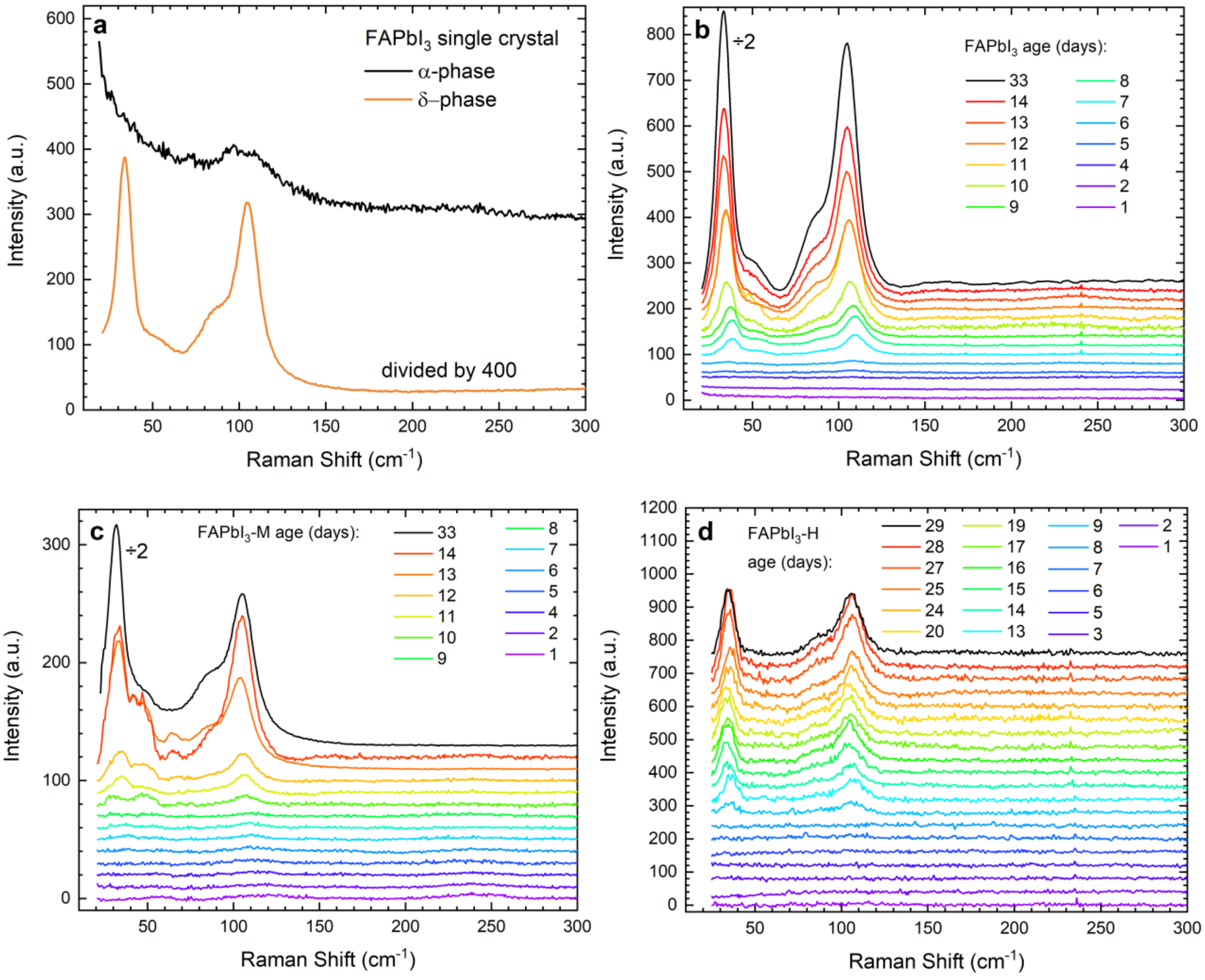

Figure S9: Investigating α-FAPbI₃ single crystal with Raman spectroscopy. Raman spectra of different $FAPbI_3$ single crystals. **(a)** Comparison of α-phase and δ-phase $FAPbI_3$. **(b) – (d)** Aging of $FAPbI_3$, $FAPbI_3$-M and $FAPbI_3$-H single crystals in ambient conditions. All crystals were kept at ambient conditions in the Raman setup for the duration of the entire stability experiment and multiple spots on the surface were compared, which resulted in a relative deviation of about 25% between peak intensities measured at different positions on the same day.

The peak splitting in the broad delta phase peaks at 55 cm^{-1} and 83 cm^{-1} that occurs in some of the $FAPbI_3$-M spectra in **Figure S9c** indicates a structural change within the δ-phase[12]. The peak splitting has been observed in all crystal types independent of additives and does not affect the peak at 108 cm^{-1} used in our analysis.

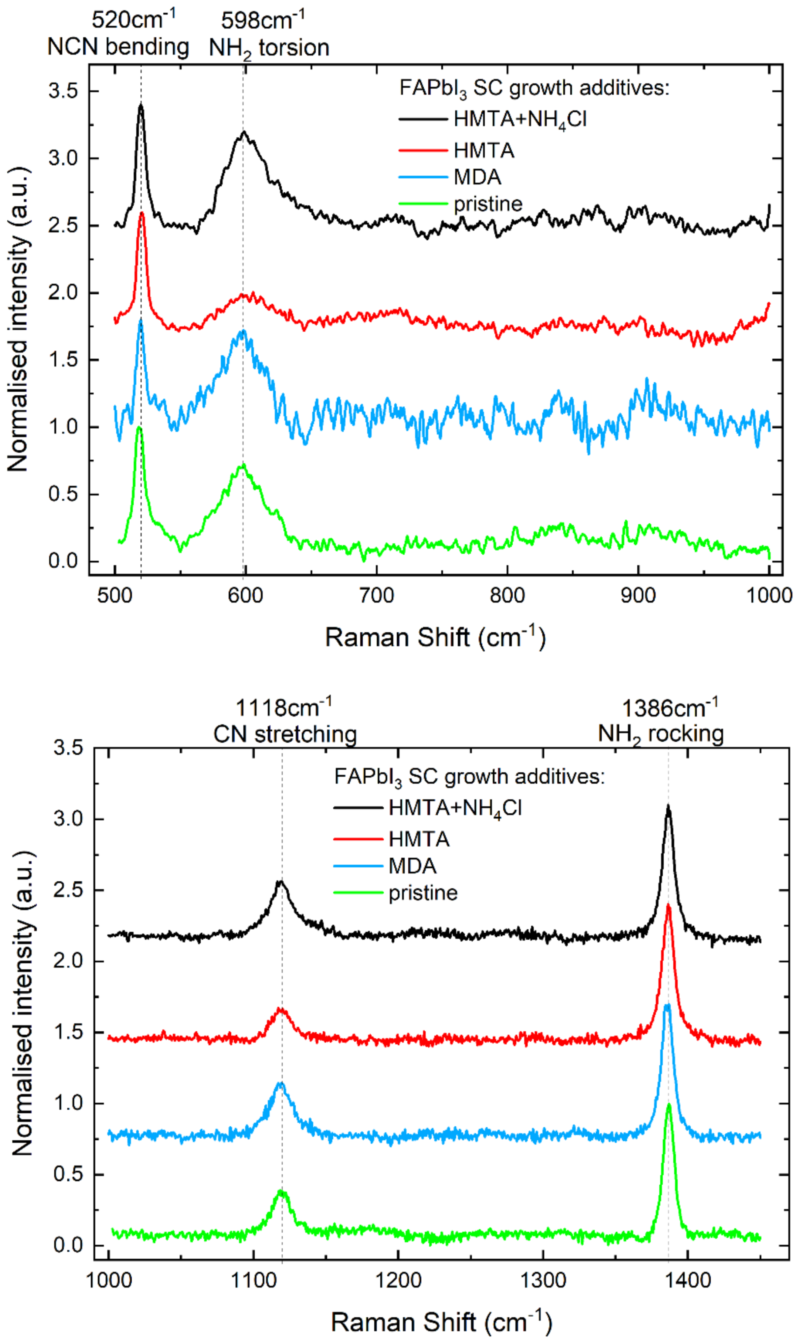

Figure S10: Non-FA$^+$ organics in $FAPbI_3$ single crystals by Raman spectroscopy. Raman spectra of $FAPbI_3$ (pristine), $FAPbI_3$-M ($MDACl_2$), $FAPbI_3$-H (HMTA) and $FAPbI_3$-H-NH_4Cl (HMTA+NH_4Cl) in the region in which vibrational modes corresponding to the organic components in the perovskite material are observed. There is no evidence of the inclusion of species besides FA$^+$ visible on the resolution of this measurement. We note that the frequency of C=N stretching vibrational modes corresponding to mim$^+$ are expected to be very similar to those of FA$^+$.

Section S6: Liquid-state Nuclear Magnetic Resonance Spectroscopy.

For experiments represented in **Figures S12, S13, S16**:

^{1}H-NMR spectra (in DMSO-d^{6}) were taken on a Bruker NMR spectrometer (300 MHz) at room temperature. Chemical shifts (δ) are reported in ppm. The signal from residual non-deuterated DMSO solvent is used for reference.

For experiments represented in **Figures 2a-c, S11, S14, S17, S18, S20-22**:

A two-channel Bruker Avance III HD Nanobay 400 MHz instrument running TOPSPIN 3 equipped with a 5 mm z-gradient broadband/fluorine observation probe is used. The signal from residual non-deuterated DMSO solvent is used for reference.

For experiments represented in **Figures 3a, 3b**:

Spectra were acquired with a Bruker AVIIIHD 600 equipped with a BBO prodigy probe. Directionless enhancement of polarisation transfer (DEPT) experiments were carried out as described by Doddrell, et al.[18].

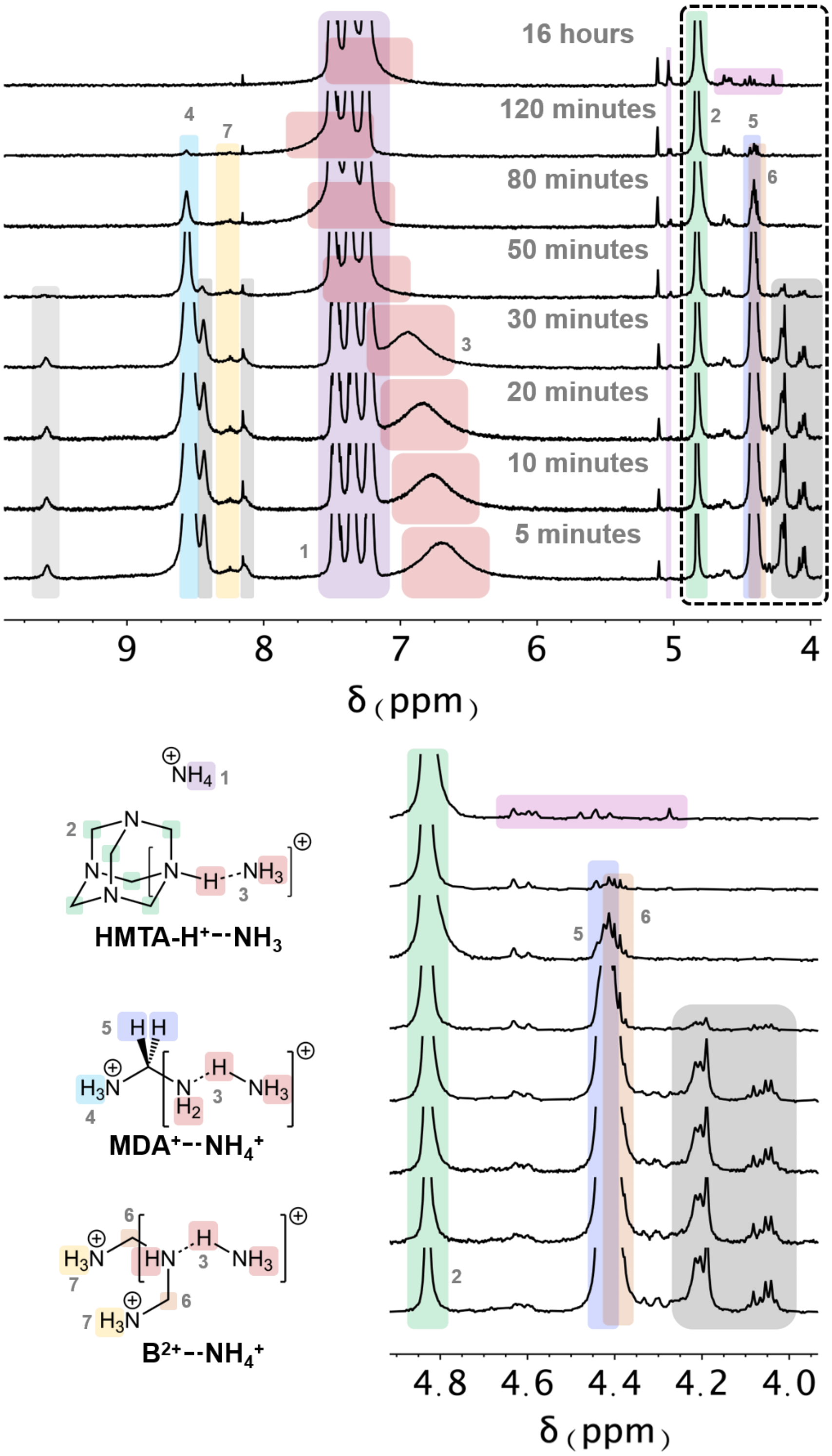

Figure S11: MDACl$_2$ degradation: Expanded spectra. ^{1}H liquid NMR spectra of MDACl$_2$ degrading in a solution of DMSO-*d*^{6}. Inset shows region in which two quartets are resolved, evolving over approximately the same timescale. Trace signals highlighted in grey are present in solution initially but disappear over time. Those highlighted in pink appear during the degradation. These correspond to unidentified intermediate species that are unstable and stable in the final equilibrium mixture (which is

dominated by HMTA-H^+ and NH_4^+), respectively. Further discussion on the identification of intermediate species indicated is presented below.

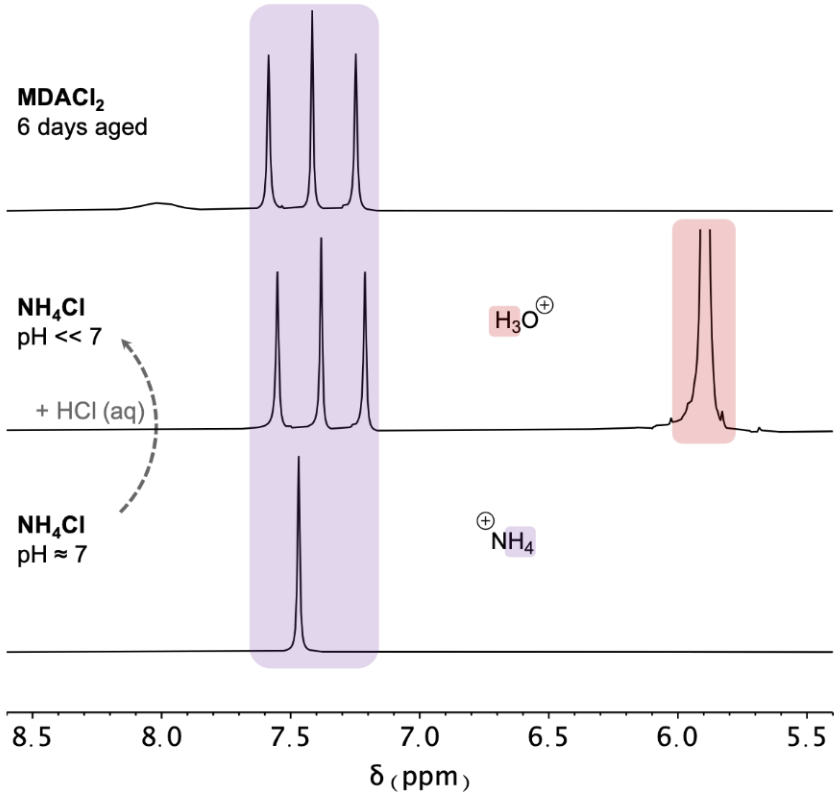

Figure S12: Demonstrating NH_4^+ as a degradation product. ^1H solution nuclear magnetic resonance spectra of NH_4Cl dissolved in d^6-DMSO (bottom) and with a droplet of HCl (aq) added (middle) to mimic the acidic environment of the solution upon in-situ $MDACl_2$ degradation. A solution of fully degraded (6 days) $MDACl_2$ is shown for comparison (top).

We note that the coupling observed as a result of ^1H interaction with the quadrupolar I = 1 ^{14}N nuclei is typically only observed in the case of a near-zero electric field gradient at the quadrupolar nucleus, otherwise quadrupolar relaxation becomes too rapid to permit coupling[19]. This suggests a species with a high degree of molecular symmetry. Moreover, the substantial coupling constant observed precludes anything but a ^1J interaction, implicating direct ^{14}N-^1H bonding and leaving NH_4^+ as the only likely candidate species.

We note that the $^1J_{14N-1H}$ coupling characteristic of ammonium is not initially observed in the spectrum of NH_4Cl (pH unadjusted). This could be due to a breakdown in the tetrahedral symmetry of the cation caused by either the rapid exchange of acidic H^+ in solution, or the close association of the chloride ion with the cation. Addition of hydrochloric acid to the NH_4Cl solution simulates the pH environment of

ammonium formed during the degradation of $MDACl_2$ and reveals the expected 1:1:1 triplet, while also shifting the ^1H signal upfield and aligning it with that observed in the degradation of MDA^{2+}.

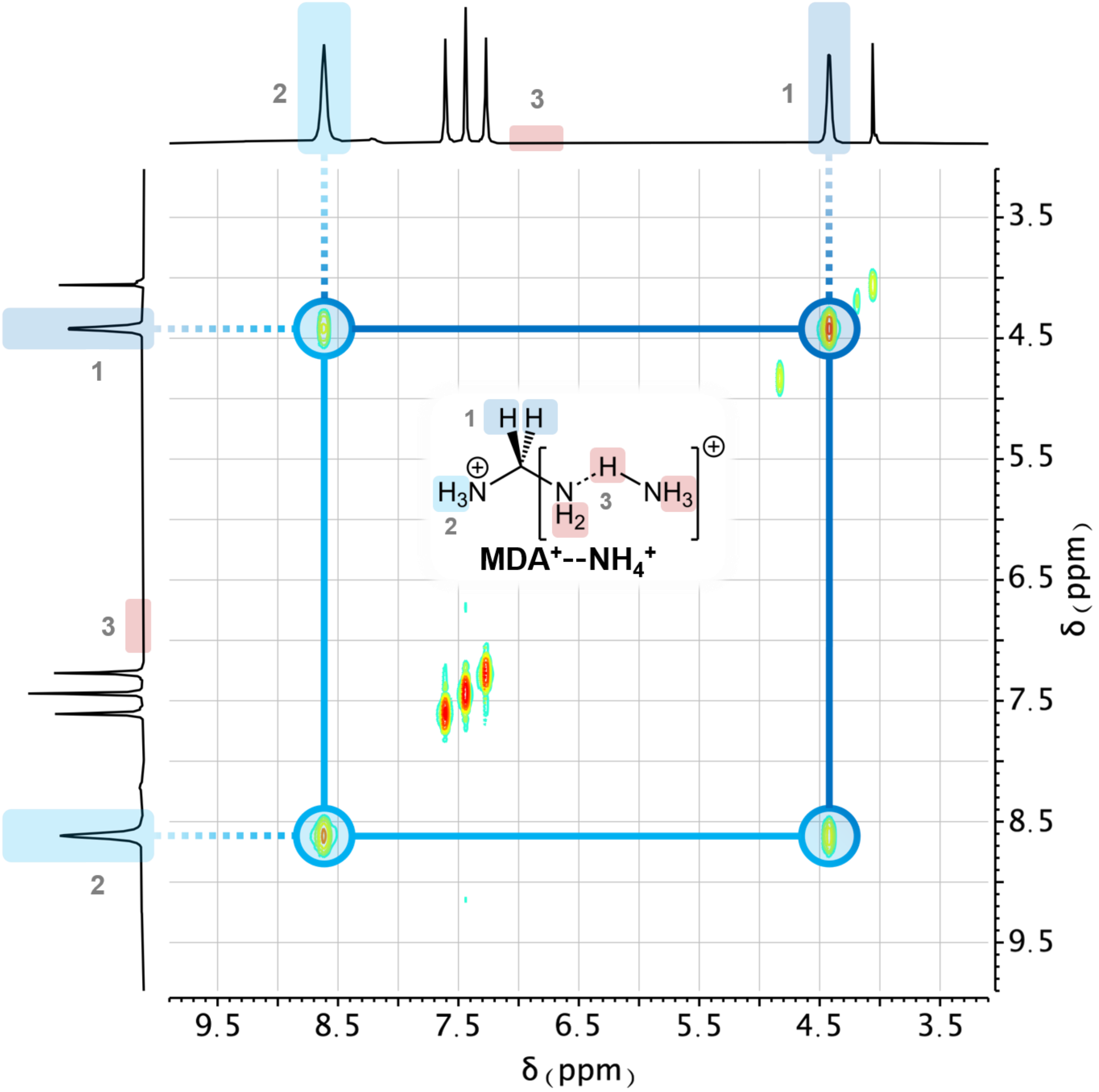

Figure S13: Intermediate species in $MDACl_2$ degradation. ^1H-^1H solution correlation spectrum of $MDACl_2$ degradation in-situ five minutes after initial dissolution in DMSO-d_6. ^1H-^1H correlation spectroscopy (COSY) reveals that, besides signals corresponding to NH_4^+ and HMTA-H^+, the two substantial signals in the ^1H solution NMR of $MDACl_2$ solutions after 5 minute correspond to nuclei present in the same species. Integration of these signals shows a near 2:3 correspondence of the signals (4.42 ppm : 8.62 ppm).

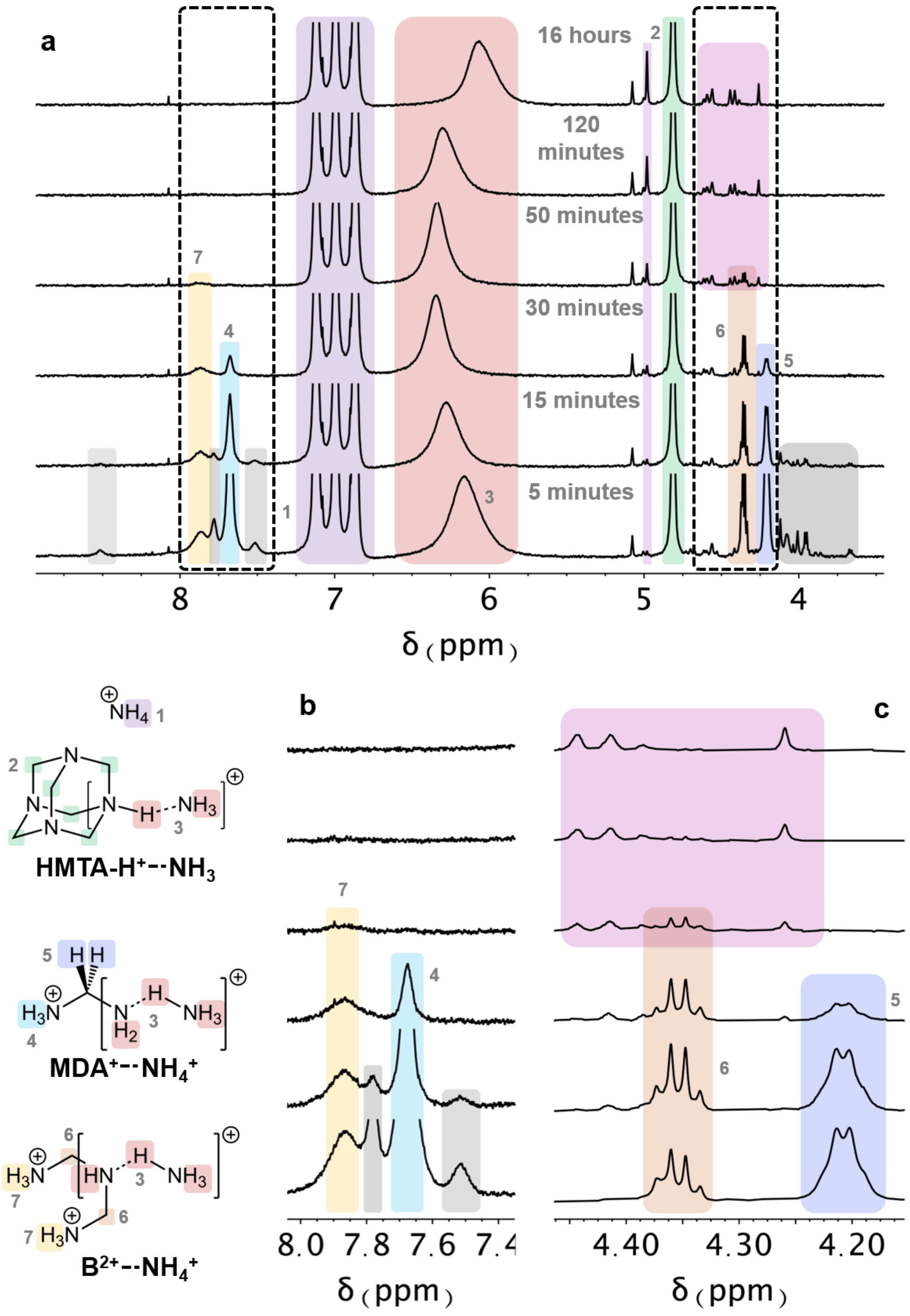

Figure S14: $MDACl_2$ (3.8 mol%) degrading in presence of PbI_2. ^{1}H liquid NMR spectra of 3.8 mol% $MDACl_2$ degrading in a solution of 1 M PbI_2 dissolved in DMSO-d^6. Inset shows regions show two quartet signals no longer overlapping (as in **Supplementary Figure S11**). We attribute this to the presence of Pb^{2+} and especially halide (I^-) ions in solution, which coordinate the organic cations in solution affecting their electron density distributions and thus their chemical shifts. We note that the addition of PbI_2 causes almost all signals to migrate substantially upfield (to lower ppm), but does not substantially change the rate of degradation. We include these spectra as they incidentally allow us to

clearly isolate signals **4-7**, which is not wholly possible in **Supplementary Figure S11** where PbI_2 is not added. Trace signals highlighted in grey are present in solution initially but disappear over time. Those highlighted in pink appear during the degradation. These correspond to unidentified intermediate species that are unstable and stable in the final equilibrium mixture (which is dominated by HMTA-H^+ and NH_4^+), respectively. Further discussion on the identification of intermediate species indicated is presented below.

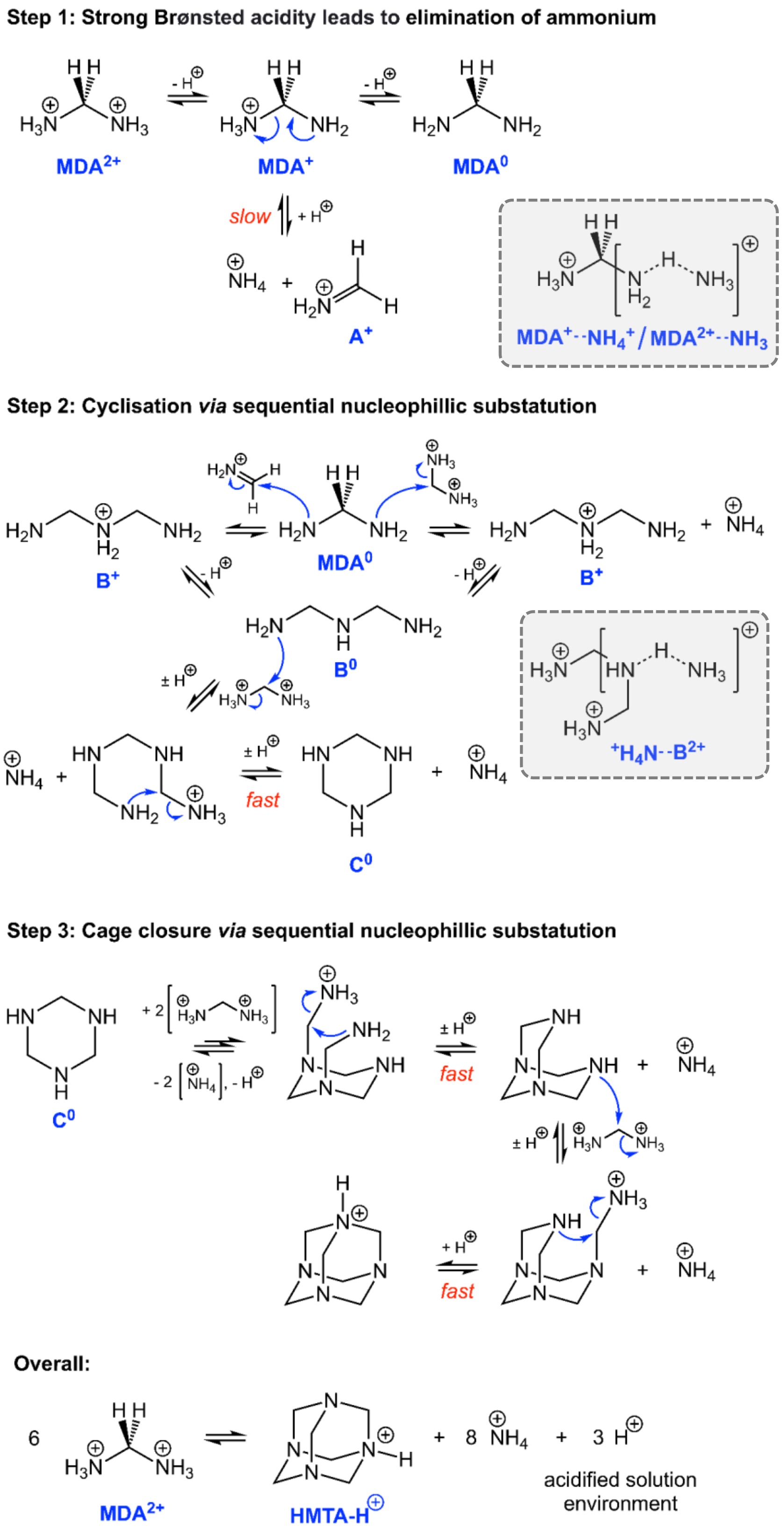

Figure S15: MDA^{2+} degradation mechanism. Full mechanistic description of MDA^{2+} into ammonium and hexamethylenetetrammonium (HMTA-H^{+}), resulting in an acidified growth environment containing these products for our $FAPbI_3$-M single crystals.

Comments on the assignment of signals to intermediates in $MDACl_2$ degradation

From the concurrent evolution and disappearance of the signals at 7.87 and 4.35 ppm (**7** and **6**) in **Supplementary Figure S14** we infer that these signals correspond to ^1H nuclei in the same intermediate molecule. Similarly, the signals at 7.68 and 4.21 ppm (**4** and **5**) evolve together. From the lineshape of the signal at 4.21 ppm, we identify that – in the absence of PbI_2 – this signal appears at 4.42 ppm (**5**) and evolves with that at 8.56 ppm (**4**) (**Supplementary Figure S11**). These two signals are also shown to be spin-spin coupled by COSY (**Supplementary Figure S13**). Signals **6** and **7** are also present when $MDACl_2$ degrades in DMSO-d^6 in the absence of PbI_2 (observed at 4.39 and 8.24 ppm, respectively), however their integration values are very low. By comparison, signals **6** and **7** integrate to comparable values to that of the primary intermediate identified (signals **4** and **5**, **Supplementary Figure S11**) when PbI_2 is added to the solution, suggesting that the Pb^{2+} and I^- ions act to stabilise the former species, most likely by formation of stable coordination interactions. This is consistent with the substantial changes in chemical shift observed for most ^1H signals upon addition of PbI_2.

In both the case of signals **4** and **5**, and of **6** and **7**, as no other signals evolve at the same rate we deduce that these correspond to the only two ^1H environments in these intermediate that are expected to give independently resolvable ^1H NMR signals. We further note that, given this, the fact that in both sets of signals the spin-spin line-splitting (quartet splitting, caused by spin-spin coupling to three adjacent inequivalent ^1H nuclei – typically 1-4 bonds distant – which are themselves all identical) is only observed in one of the signals suggests that the ^1H nuclei present in the coupled (downfield) environment (signals **4** and **7**, respectively) are exchanging slowly with available acidic H^+ in solution, leading to a degree of exchange broadening in the corresponding ^1H NMR signal that masks the line splitting caused by spin-spin coupling. Candidate ^1H environments that might fit these data are limited to isolated ammonium ($-NH_3^+$) groups. Although iminium ($R_2C{=}N\mathbf{H}_2^+$) protons might also be expected to exchange in solution, these are typically observed at higher chemical shift[20], and do not result in the spin-spin quartet coupling observed in adjacent ^1H environments.

The presence of a substantially broadened signal (**3**) that migrates over time between 6.7-7.5 ppm as $MDACl_2$ degrades (**Supplementary Figure S11**) or 6.1-6.4 ppm when PbI_2 is added to $MDACl_2$ (**Supplementary Figure S13**) suggests that other chemical exchanging ^1H nuclei are also present in

the degradation solution. Given the large integration of this value (which suggests a large population of exchanging H^+, as expected in the acidic environment generated by MDA^{2+} degradation), and the varying chemical shift of this signal, the most likely explanation for this is that the signal represents ^1H nuclei involved in coordinated amine-ammonium hydrogen bonding in solution and thus undergoing very rapid proton exchange faster than the timescale of the NMR experiment. As a result, all ^1H nuclei in this population are expected to appear as a single broad signal at a chemical shift corresponding to the weighted average of the two (or more) contributing signals, and integrating to a value representing the total population of exchanging ^1H. That ^1H nuclei in such environments result in a mixed-signal, rather than a broadened but isolated signal (as in the case of signals ammonium signals **4** and **7**), is attributed to the rate of proton exchange in coordinated amine-ammonium species. If such coordination species are long-lived in comparison to proton exchange within them then such exchange is a monomolecular process and thus expected to be rapid. A weighted-average signal is expected if the timescale for proton exchange is also comparable to the timescale of the NMR experiments. By contrast, in the isolated case proton exchange is a bimolecular process requiring interaction between an ammonium group and a suitable base free in solution, and thus expected to be slower and lead only to the observed broadening effect of the ammonium signal.

This hypothesis is further supported by the absence of any signals in the 0-4 ppm region corresponding to isolated amine signals. MDA^{2+} is weakly acidic in polar solvents. Thus, in order to release amine groups ($R\text{-}NH_2$) into solution, at least a stoichiometric quantity of acidic H^+ must be released concurrently (i.e. for every amine group free in solution at a given time, there is at least one additional H^+ present in the solution). Given that amines are also potent nucleophiles, and the majority of intermediates in the proposed mechanism (**Supplementary Figure S15**) possess electrophilic groups, the build up of free amines in solution is unlikely. This is in part why the degradation proceeds so rapidly. In fact, the only plausible way in which a species containing an amine moiety might be stabilised in solution for any length of time is by coordination of that amine group by an ammonium moiety, most like NH_4^+ itself given the presence of large quantities of this species in solution even at the earliest stage of degradation. We thus identify the broad signal, **3**, as a weighted-average of all ^1H nuclei involved in any $R_3N\text{-}N\mathbf{H}_4^+$ (or, equivalently, $R_3N\mathbf{H}^+\text{-}N\mathbf{H}_3$) coordination bonds (where R may also be ^1H). Migration of this signal over time is therefore attributed to the increasing acidity of the solution environment as

degradation proceeds and the evolving contribution of a wide range of transient intermediate species forming and being consumed during degradation.

Not only does the rapid proton exchange evident in coordinated amine-ammonium groups lead to observation of a single mixed signal, but it also effectively suppresses spin-spin coupling between ^1H involved in coordination and adjacent ^1H. If this were not the case, resolution of 1:2:1 triplet splitting of signals corresponding to ^1H neighbouring amine moieties could be expected, which we do not observe.

In light of the above, and based on our proposed mechanism, we suggest the most likely identity of the principal intermediate observed (signals **4** and **5**) is NH_4^+-coordinated MDA^+. As set out in the main text, the singly protonated diaminomethane is expected to be stabilised with respect to either the diammonium or diamine equivalents. Moreover, spin-spin coupling between the ammonium and the methylene ^1H nuclei results in the expected quartet splitting observed in the latter signal (**5**). In a 2015 mechanistic study on the formation of HMTA from ammonia and formaldehyde under basic conditions, Zeffiro et al. found that reaction of bis(aminomethyl)amine (**B**) towards HMTA is unexpectedly disfavoured energetically[21], suggesting that this species may accumulate in solution as a transiently stable intermediate. However, this analogy is weak on account of the wholly different pH conditions expected during HMTA formation from MDA^{2+}. The acidic environment not only precludes accumulation of a neutral amine, such as **B**, but also means the most likely electrophiles available for nucleophilic substitution prior to cage closure are ammonium compounds rather than methanimine (CH_2=NH) as in the case of Zeffiro et al. However, under acidic conditions formation of doubly protonated $\mathbf{B^{2+}}$ is possible, with the resulting dication being less charge-dense than MDA^{2+}, and thus likely more stable. $\mathbf{B^{2+}}$ is mechanistically relatively stable to both backward reaction (*via* attack by ammonia, which is strongly basic and thus almost entirely protonated in solution or involved in coordination with ammonium groups, or *via* kinetically inhibited elimination) and forward reaction, at least while substantial quantities of MDA^{2+} remain in solution as the latter is more charge-dense, and thus more electrophilic than $\mathbf{B^{2+}}$. Coordination of the amine moiety of $\mathbf{B^{2+}}$ by NH_4^+ is expected, as discussed above, and thus spin-spin coupling of the methylene ^1H nuclei (**6**) to an adjacent ammonium group (**7**) produces the expected quartet signal. The presence of quartet splitting in both signals **5** and **6** assists greatly in reducing the number of candidate species. However, given the nearly identical nature of the two sets of intermediate signals we have highlighted, it is not possible to conclusively distinguish which of these corresponds to MDA^+-NH_4^+ and which to $\mathbf{B^{2+}}$- NH_4^+. Our assignment is based on the observation that signals **4** and **5**

disappear earlier in the degradation process suggesting the species giving rise to them occurs earlier in the degradation process.

We note that the apparent quartets observed for signals **5** and **6** might be 'AB quartets', i.e. heavily roofed doublet of doublets. These are typically associated with locked ring systems in which axial and equatorial ^{1}H nuclei are inequivalent, but spin-spin coupled with a large ^{2}J coupling constant. If this were the case then one of the two pairs of signals discussed could correspond to 1,3,5-trazinanium (**C^{+}**). However, AB quartets typically show non-uniform spacing between their constituent peaks unless ^{2}J splitting and the chemical shift difference between the two isolated signals is perfectly matched. As we find that peak spacing is highly uniform in both signals (**Figure 2c**) and the exception described is highly unusual, we conclude this interpretation is very unlikely.

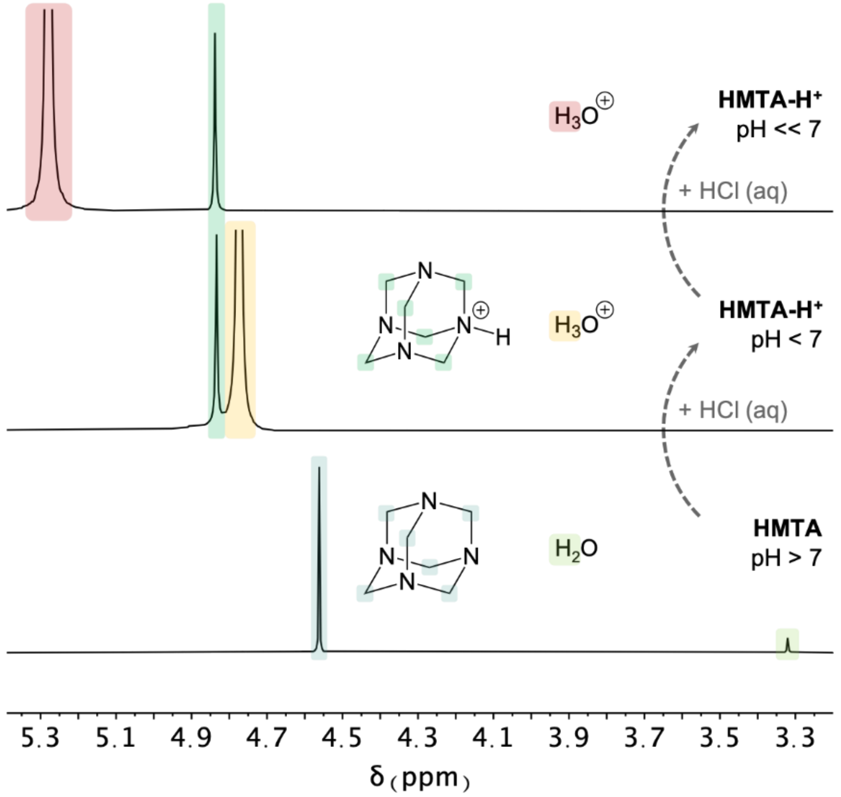

Figure S16: Demonstrating HMTA-H$^+$ as a degradation product. ^{1}H solution nuclear magnetic resonance spectra of HMTA dissolved in d^6-DMSO (bottom) before stepwise addition of droplets of HCl (aq) (middle, top) to mimic the acidic environment of the solution upon in-situ $MDACl_2$ degradation.

Although the stepwise reduction in pH leads to a gradual downfield shift in signals corresponding to ^{1}H nuclei in water molecules, as expected, a single discrete downfield shift is observed in the signal corresponding methylene ^{1}H in HMTA due to the monoprotonation of HMTA to HMTA-H$^+$. Further reduction in pH has negligible effect on this signal. We note that in the monoprotic form, the acidic H$^+$ need not be localized on a single amine functional group, but instead may be captured inside the cage *via* equivalent interactions with all amine groups.

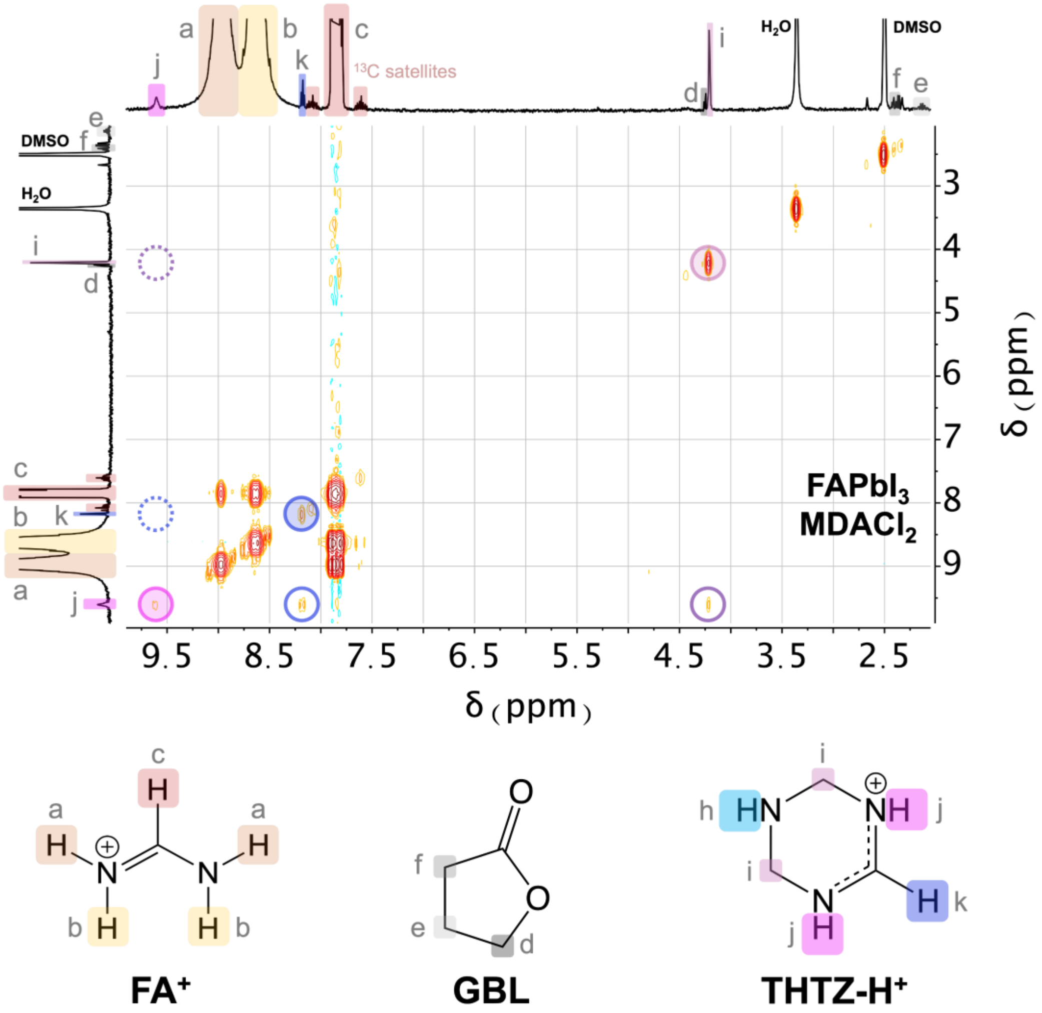

Figure S17: THTZ-H$^+$ formation from MDACl$_2$ degradation in presence of FA$^+$. ^{1}H-^{1}H 2D correlation spectrum showing off-diagonal intensity denoting correlation between ^{1}H nuclei in the chemical environments giving rise to signals at 8.18 and 9.60 ppm, and between those at 4.21 and 9.60 ppm. This not only suggests that these chemical environments are present in the same molecule, but further confirms the signal assignment of THTZ-H$^+$ in which correlation between signals at 4.21 and 8.18 ppm is not expected as these ^{1}H nuclei are too greatly separated in the molecule. The absence of corresponding off-diagonal intensity in the top left of the spectrum, as would be expected, is concerning but not altogether unexpected given the trace quantities of THTZ-H$^+$ in solution. Note: this 2D COSY spectrum was obtained from a sample of a FAPbI$_3$-M single crystal dissolved in d^6-DMSO.

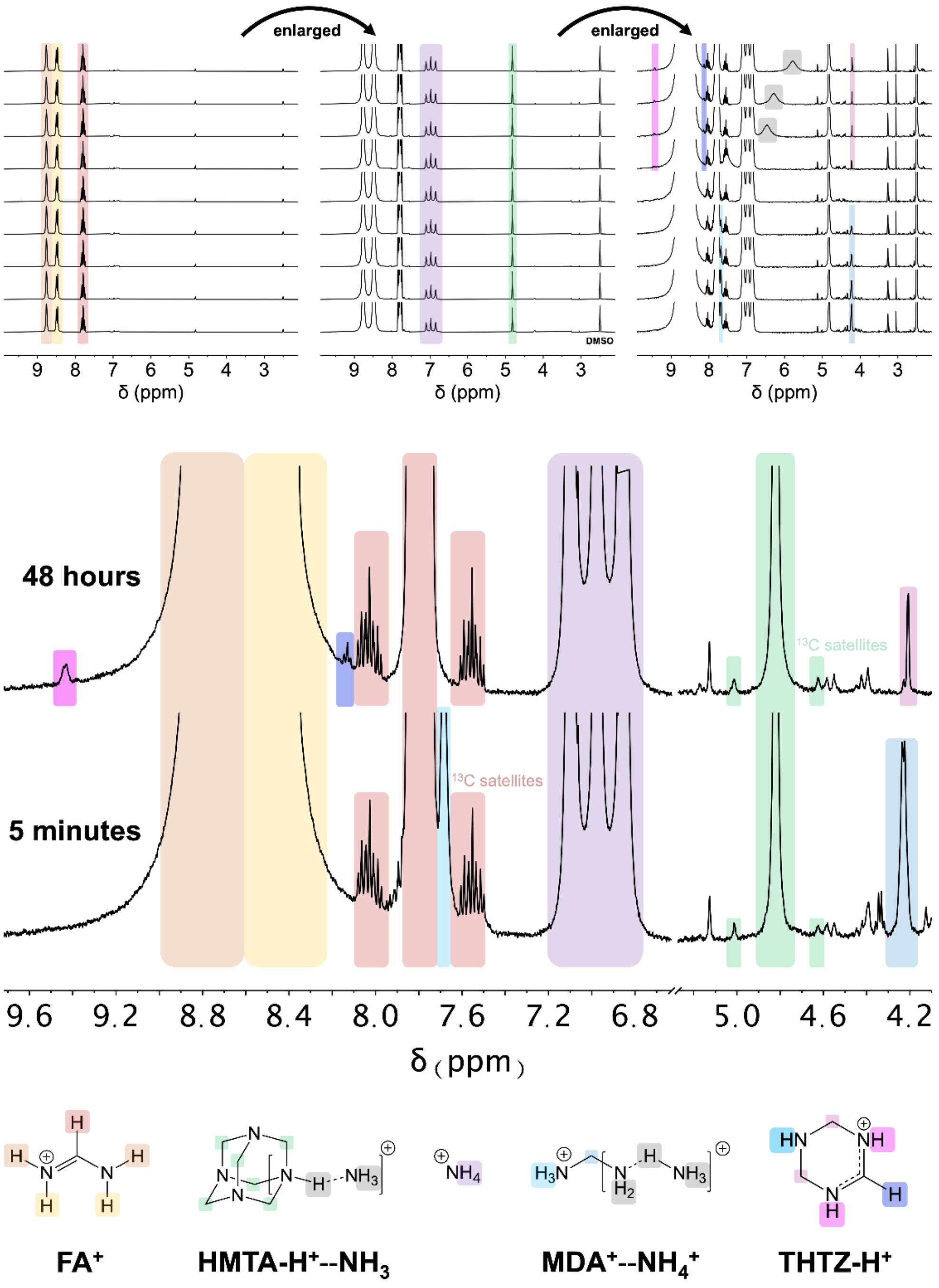

Figure S18: THTZ-H$^+$ formation from MDACl$_2$ degradation in presence of FA$^+$. ^{1}H solution nuclear magnetic resonance (NMR) spectra of MDACl$_2$ (3.8 mol%) aged in the presence of formamidinium iodide (FAI) and lead (II) iodide (PbI$_2$). Top row: gradual enlargements of stacks of full spectra taken at (bottom to top): 5 minutes, 10 minutes, 15 minutes, 20 minutes, 1 hour, 2 hours, 12 hours, 24 hours, 36 hours, 48 hours. Signals corresponding to THTZ-H$^+$ appear as minority degradation products (beside NH_4^+ and HMTA-H$^+$) from around 36 hours after dissolution of the precursors. The NMR tubes in which the experiment was conducted were sealed in a N_2 glovebox and stored at room temperature during

aging. The absence of a fourth ^{1}H signal corresponding to the THTZ-H^+ secondary amine environment is likely due to rapid acidic H^+ exchange in solution (exchange broadening), as commonly observed in amines.

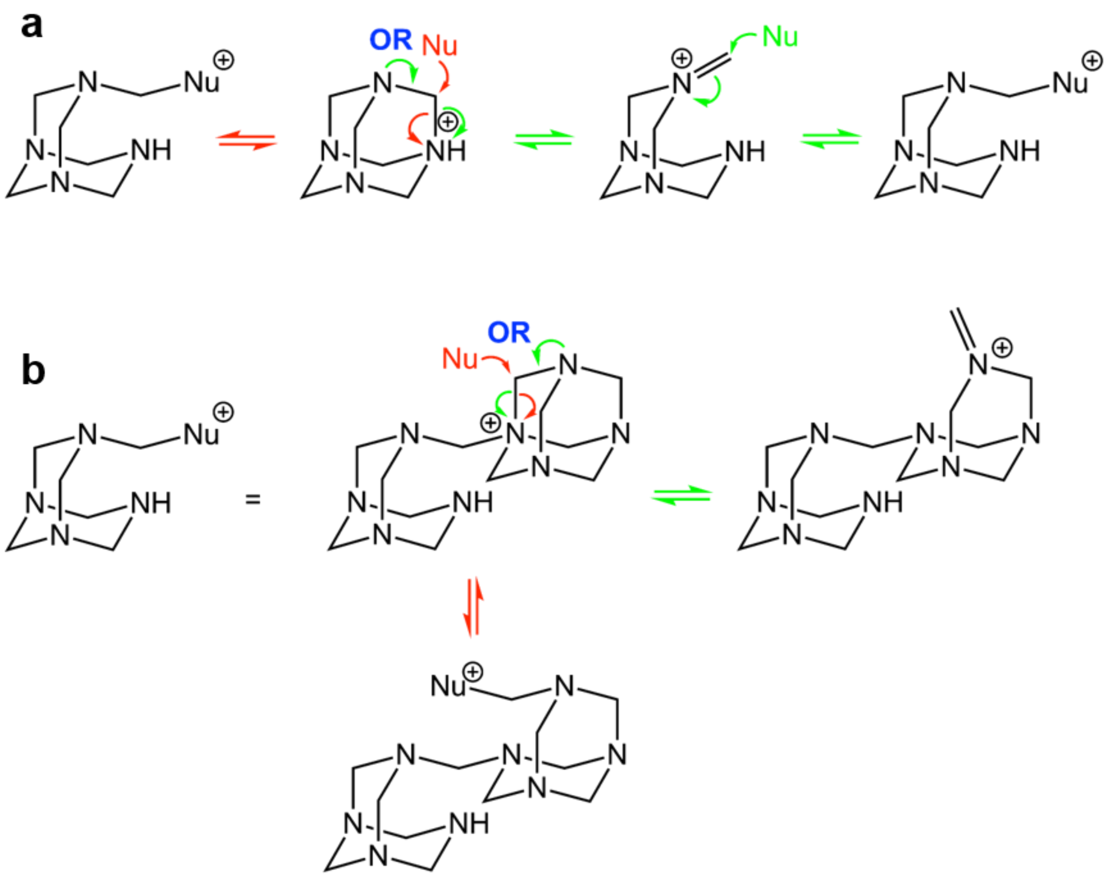

Figure S19: Mechanistic justification for THTZ-H$^+$ formation from HMTA. a) Equivalence of nucleophilic substitution or elimination in breakdown of HMTA. **b)** Activity of HMTA as a nucleophile catalysing breakdown of other HMTA molecules towards methanimine.

In **Supplementary Figure S19a** we demonstrate that, although mechanistically distinguishable, intramolecular elimination within HMTA-H$^+$ with subsequent attack by an appropriate nucleophile available in solution (for example HMTA) leads to the same outcome as direct attack by that nucleophile.

In **Supplementary Figure S19b** we show that when HMTA acts as a nucleophile in attacking HMTA-H$^+$ the resulting species can undergo further reaction on either of the two HMTA moieties. In principle, formation of polyamines of any size are possible by this mechanism. However, in all cases, ultimately for THTZ-H$^+$ formation to occur isolation of a primary amine group, with subsequent elimination of methanimine (CH_2=NH), must take place (as set out in **Figure 3c**). Although elimination reactions are typically very slow at room temperature, the larger the polyamine the greater the entropic gain of elimination. Thus, a negative feedback loop is establish by which formation of larger polyamines by attack of further HMTA becomes increasingly less favourable with increasing size.

For simplicity, the mechanism shown in the main text only produces a single methanimine (CH_2=NH) from each HMTA consumed. This is sufficient to demonstrate mechanistically that THTZ-H^+ can form in-situ in solution containing multiple HMTA molecules and FA^+. However, in a protic environment (such as that provided by mixture with excess FA^+), the remains of the HMTA cage show in **Figure 3c** can itself degrade further by addition – and subsequent consumption of – HMTA acting as a nucleophile. Overall, and disentangling the requirement for multiple HMTA molecules playing a role, each single HMTA molecule is expected to give rise to four methanimine molecules.

By contrast, each MDA^{2+} cation added produces only a single methanimine. Thus, addition of 3.8 mol% $MDACl_2$ produces a maximum 3.8 mol% mim^+, while 0.63 mol % HMTA produces a maximum of 2.53 mol% mim^+ in solution.

We emphasise that, as with all mechanistic details presented in this work, the schemes set out should be interpreted as mechanistic justifications only, demonstrating that – by established chemical mechanisms – it is possible for the observed species to have been produced from one another. We have not conducted extensive mechanistic studies, and have only identified a relatively small number of the species displayed in the mechanistic schemes.

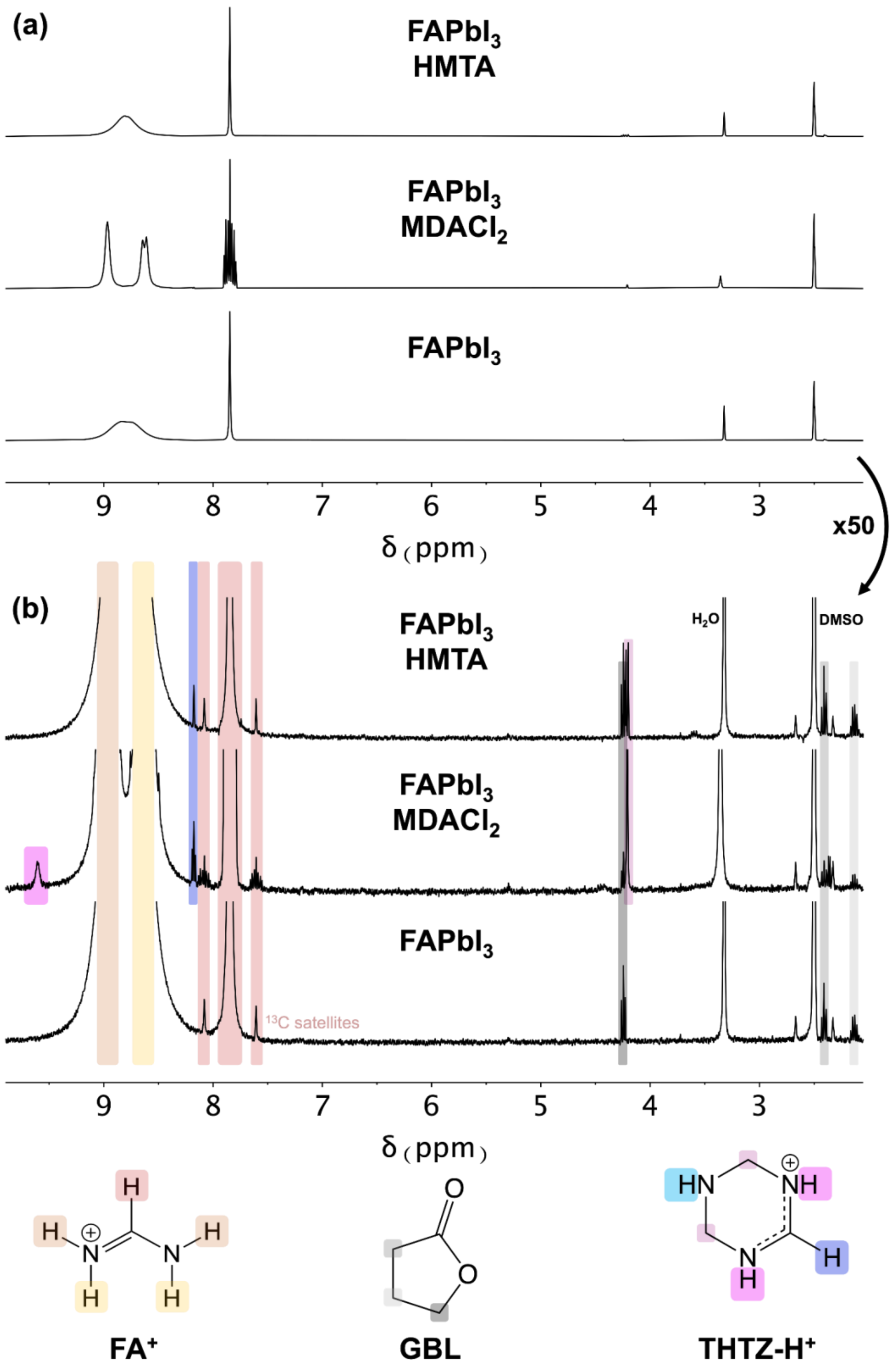

Figure S20: THTZ-H^{+} detection from single crystal samples. Full and expanded versions of spectra shown in main text Figure 3b. ^{1}H solution nuclear magnetic resonance spectra of $FAPbI_3$, $FAPbI_3$-M and $FAPbI_3$-H single crystals. Small quantities of remnant GBL (processing solvent) are detected.

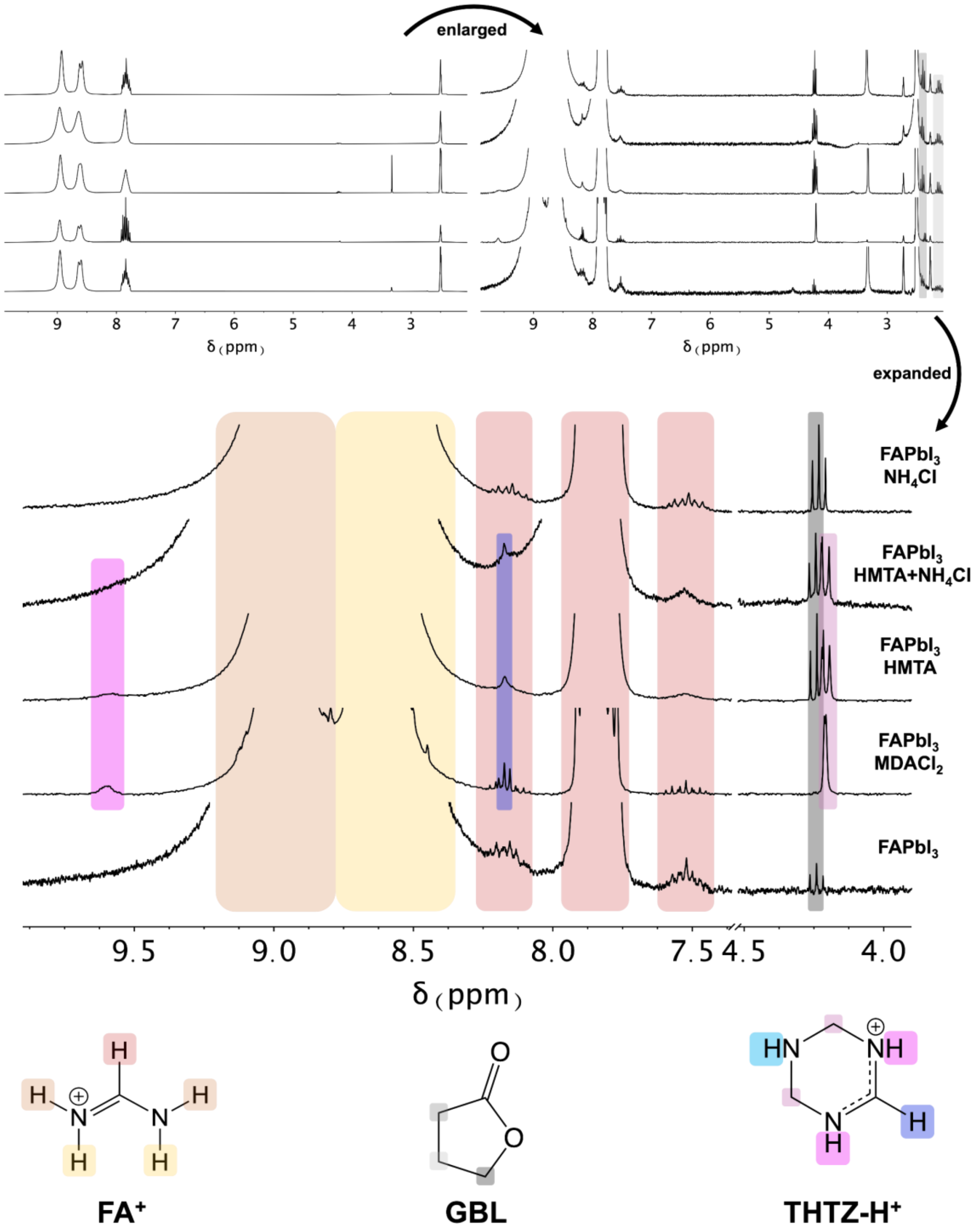

Figure S21: THTZ-H$^+$ detection from single crystal samples. ^{1}H solution nuclear magnetic resonance spectra of $FAPbI_3$ single crystals that have been grown in the presence of a range of additives (3.8 mol% $MDACl_2$, 0.63 mol% HMTA, 5.07 mol% NH_4Cl, and both 0.63 mol% HMTA and 5.07 mol% NH_4Cl) showing that THTZ-H$^+$ is observed in any crystal in which HMTA or $MDACl_2$ are employed.

Comments on the appearance of signals corresponding to FA^+

Figures 3a and S19-20 show the ^1H solution NMR spectra of $FAPbI_3$ single crystals grown in the presence of a range of additives. Crystals in **Supplementary Figure S19** were all made in the same crystal growth batch. All these spectra were taken on a 400 MHz Bruker spectrometer. Crystals analysed in **Figure S20** were all made in the same crystal growth batch, but a different batch to those in **S19**, and their spectra were taken on a 300 MHz Bruker spectrometer. There is a marked difference in the appearance of signals corresponding to FA^+ in the spectra, both in whether two amidinium ^1H chemical environments are resolved, and in the degree of resolution of vicinal ^1H-^1H coupling between amidinium and methine ^1H nuclei. In **Figure S20**, the two amidinium environments (which differ in whether they are *E* or *Z* with respect to the methine ^1H environment, and are resolvable because of the reduced rate of rotation of the conjugated N-C-N system) are distinguishable in all samples. In **Figure S19**, only $FAPbI_3$-M shows the expected two singlets. It has previously been shown[22] that the linewidth of ^1H NMR spectra (of perovskite solutions) is sensitive to the acidity and/or halide content of the solution in which the perovskite materials are dissolved. In general, line-broadening can also occur due to unsuccessful or incomplete shimming of the sample before spectrum acquisition. Considering we have observed the THTZ-H^+ in the spectra of single crystals in which the amidinium environments were not resolved, and in those in which the amidinium environments are resolved, we do not believe this difference is related to the chemistry giving rise to enhanced α-phase stability. Equally, in **Figure S20**, $FAPbI_3$, $FAPbI_3$-M and $FAPbI_3$-NH_4Cl single crystals all give rise to spectra in which a 1:2:1 triplet of 1:2:1 triplets is observed at 7.86 ppm, corresponding to the methine ^1H nucleus of FA^+, while the analogous signal in $FAPbI_3$-H and $FAPbI_3$-H-NH_4Cl samples appear as singlets. Close inspection of this methine signal in $FAPbI_3$-H, however, shows a degree of shouldering consistent with the presence of partially resolved $^3J_{H-H}$ coupling. Given the apparently absence of correlation, and the irreproducibility of these effects (we have, for example, also observed $FAPbI_3$ single crystals to give rise to spectra in which vicinal coupling is not resolved) we again infer that the cause of these coupling regimes is independent of the stability enhancement observed in $FAPbI_3$-M and $FAPbI_3$-H single crystals.

Comments on the appearance of low intensity signals in ^{1}H NMR spectra throughout

In main text **Figures 2a-b and 3a**, and in **Supplementary Figures S16-17, S19-21** satellite signals caused by $^1J_{13C\text{-}1H}$ coupling in FA molecules containing ^{13}C (approximately 1 mol %) are often visible for both methine and amidinium ^{1}H nuclei environments.

Also apparent in the spectra of many of our single crystals are signals corresponding to trace quantities of GBL, the processing solvent; 4.24 ppm (2H, t), 2.40 ppm (2H, m) and 2.12 ppm (2H, m). Although we have observed the presence of excess residual GBL on the surface of as-grown crystals to introduce variability in their α-phase stability, all these crystals have been heated at 180 °C for 30 minutes under vacuum and other grown in the same batch (and so presumably also containing trace quantities of GBL) show comparable phase stability to their peers where trace GBL is not detected.

Also apparent is a signal corresponding to H_2O at 3.33 ppm. The presence of water in spectra corresponding to dissolved single crystals in particularly crucial. As the amount (indicated by integration of the signal) of water present seems to be variable, we do not attribute this only to H_2O introduced with the d^6-DMSO solvent, although this is common. Some water may come from incomplete drying of NMR tubes used to prepare samples. However, there is also likely a degree of moisture adsorption on the surface of the crystals that is highly dependent on factors such as surface to volume ratio, storage conditions and crystal age. Across our study, single crystals were typically stored in vials sealed under nitrogen. However, there is substantial variability in crystal size, and when multiple measurements are made on a crystal it may be exposed to ambient air for different lengths of time. The role that differing quantities of adsorped water may play in accelerating α-$FAPbI_3$ phase transformation is unclear, but certainly worthy of further study. We note that the amount of water observed via ^{1}H NMR does not correlate with improved phase stability.

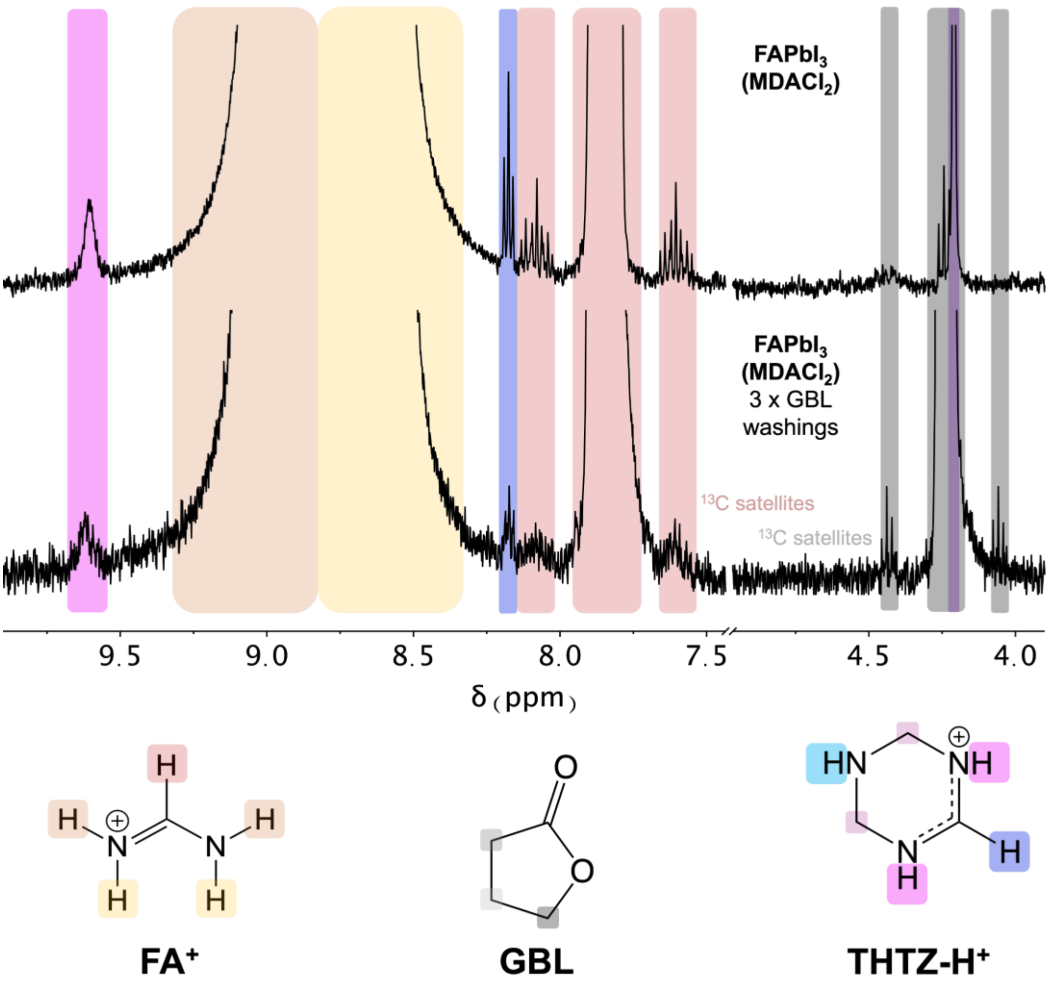

Figure S22: GBL washing to investigate THTZ-H$^+$ in bulk or on surface of $FAPbI_3$-M/$FAPbI_3$-H crystals. ^{1}H solution nuclear magnetic resonance spectra of an untreated $FAPbI_3$-M crystal dissolved in d^6-DMSO (bottom), and of a $FAPbI_3$-M crystal grown in the same batch but after vacuum drying is washed three times with 1 mL of GBL to remove the outer layers of the crystal, before addition of DMSO-*d*^{6}.

Comments on GBL washing experiments to investigate THTZ-H^+ surface layer hypothesis

There appears to be a smaller quantity of THTZ-H^+ in the spectrum of the GBL-washed $FAPbI_3$-M crystal than that of the unwashed crystal. Although integration of such small signals is challenging, this appears to be born out by relative peak integrations of the FA^+ and THTZ-H^+ signals. However, the nature of this experiment (in which a crystal is irreversibly dissolved in DMSO-d^6, and so the two spectra shown are from the dissolution of two different crystals) complicates the analysis possible. Given that surface area to volume ratio is essential in determining the expected THTZ-H^+:FA^+ stoichiometry under the hypothesis that the former is only found on the crystal surface, crystal-to-crystal variation renders quantitative analysis of this nature impossible unless there is no THTZ-H^+ present whatsoever. As we do not observe this, we cannot conclusively say that THTZ-H^+ is only present on the surface of $FAPbI_3$-M and $FAPbI_3$-H crystals.

Section S7. First Principles Calculations of Cation Steric Radii.

Table S4 shows computed cation sizes for a range of candidate A-site cations reported for lead-halide perovskites, as well as mim^+, MDA^{2+}, THTZ-H^+ and HMTA-H^+ studied in this work. We highlight that a range of different methods for estimating organic cation sizes for application in halide perovskites have been reported[23–25]. In this work, we use only the approach reported in Filip et al.[23] throughout, for consistency, and we compare where possible with cation radii as defined by Kieslich et al.[24]. We estimate the size of the MDA^{2+}, mim^+, HMTA-H^+, THTZ-H^+ and other cations as the radius of a sphere centred at the centroid of the cation charge density, which incorporates 95% of the total integrated charge.

We calculate the charge density within density functional theory (DFT)[26] as implemented in the Quantum Espresso code[27], using the Perdew-Burke-Erzerhof parametrization of the generalized gradient approximation (DFT-PBE)[28]. We used the Optimized Norm Conserving Vanderbilt (ONCV) pseudopotentials[29] as found on the Pseudo Dojo repository[30] constructed for the PBE functional to describe the atoms. The structure of all isolated cations reported in **Table S4** are optimised within DFT-PBE, by simulating vacuum using a large unit cell of 25 Å, compensating the cation of +1 with a background charge and using a plane wave cut-off of 75 Ry.

We note that, even our use of the steric – rather than ionic – radii fails to account for the substantial anisotropy of many of the organic cations discussed. The assumption that these organic cations may be considered spherical, and so assigned a radius of any kind, is in general increasingly invalid with increasing anisotropy/non-sphericity, and with the size of the organic cation. Moreover, such calculations fail to account for directed bonding interactions that exist between the A-site cation and components in the Pb-X inorganic scaffold, which may (depending on the geometry of the organic cation in question, and the distribution of its electron density) act to either stabilise or destabilise the 3D perovskite structure. All discussion presented here must be assessed with consideration of these limitations.

Notwithstanding this, however, we calculate that THTZ-H^+ is 18% larger than FA^+ and 9-10% larger than dimethylammonium (DMA^+)[31–33], ethylammonium (EA^+)[25] and guanidinium (GUA^+)[34]. These latter three cations have all been reported previously as only partially incorporated into 3D perovskites (defined as a material possessing a continuous 3D network of corner sharing BX_6 octahedra), alloyed

alongside other A-site cations (FA^+ with DMA^+ [31] and GUA^+ [34]; MA^+ with EA^+ [25]). In all these cases, the reported amount of incorporation is substantially higher than the ~0.5 mol% THTZ-H^+ found herein. Given the size of THTZ-H+ comparative to DMA+, GUA+ and EA+, it is unlikely that THTZ-H+ can be completely incorporated into the A-site of the perovskite; however, we cannot rule out the possibility of partial incorporation in small concentrations.

Table S4. List of candidate A-site cations with calculated steric[23] and effective radii[24]. Final column summarises which of these cations have been previously reported in $(A_1A_2)BX_3$ materials possessing a continuous ("3D") perovskite network of corner-sharing BX_6 octahedra.

**Cation**	**Steric Radius (Å) Filip et al.[23]**	**Effective Radius (Å) Kieslich et al.[24]**	**Experimentally reported mixed A-site alloy**
NH_4^+	1.55	1.46	
Cs^+	1.76		✔ [35]
mim^+ [$CH_2NH_2^+$]	1.93		
MA^+ [$CH_3NH_3^+$]	2.03	2.17	✔ [36]
FA^+ [$(NH_2)_2CH^+$]	2.24	2.53	✔ [37]
MDA^{2+} [$(NH_3)_2CH_2^{2+}$]	2.30		
$Imid^+$ [$C_3H_5N_2$]	2.47	2.58	✔ [38]
DMA^+ [$(CH_3)_2NH_2^+$]	2.43	2.72	✔ [31–33]
EA^+ [$CH_3CH_2NH_3^+$]	2.41	2.74	✔ [25]
GUA^+ [$C(NH_2)_3^+$]	2.40	2.78	✔ [34]
THTZ-H^+ [$C_3H_8N_3$]	2.65		
HMTA-H^+ [$C_6H_{13}N_4^+$]	2.89		
BnA^+ [$PhCH_2NH_3^+$]	3.45		
BA^+ [$C_4H_9NH_3^+$]	3.48		

We use the values in **Table S4** for steric radius as an approximation for the ionic radius, r_A, of the A-site cation to calculate the Goldschmidt tolerance factor[39], $t = \frac{(r_A + r_X)}{[\sqrt{2}(r_B + r_X)]}$, where r_B and r_X are ionic radii of the B- and X-site ions, respectively, such that a 3D perovskite structure would for if $0.8 < t \leq 1.0$, with $t = 1.0$ being geometrically 'ideal'. Based on our calculated steric radii (**Table S4**) and Shannon radii of Pb^{2+} (1.19 A) and I (2.20 A), we calculate the tolerance factor *t* for 3D ABX_3 structures based on a selection of the cations listed in **Table S4**. These are marked by solid circles in **Figure S22**. We also compute values of *t* for these structures based on values for the ionic radii of Pb^{2+} (1.42 A) and I^- (2.49 A) estimated using the same protocol as discussed above. These are marked by open circles in **Figure S22**.

We emphasise, however, both that the Goldschmidt tolerance factor is calculated on the basis of a single A-site cation and also takes no account of the globularity of organic cations[25] or any modes of bonding that may exist between the A-site cation and the haloplumbate structure in which it resides. Although it is possible to linearly combine the A-site cation radii constituting A-site alloyed perovskites and calculate a tolerance factor for the 'average structure', such an approach is limited as it further fails to take account of the possibility of – for example – co-alignment or co-misalignment of adjacent non-spherical cations and/or regular octahedral tilting in the haloplumbate network, by which mechanisms an A-site alloyed perovskite structure might be stabilised. Further, as the exact mode of THTZ-H^+ incorporation remains unclear, it is important to note that the Goldschmidt tolerance factor offers no mechanism by which to account for ion substitutions besides direct (stoichiometric) replacement of the A- B- or X-site ions in the perovskite structure. This may not be sufficient to full account for THTZ-H^+ incorporation. Improvements on Goldschmidt's tolerance factor have been reported[40–42], notably that of Bartel, et al., however most such efforts have focused on the limitations on ABX_3 structural stability of adjacent halide separation and to our knowledge none have satisfactorily addressed the limit of non-spherical A-site cations or a provided a mechanism whereby non-stoichiometric substitutions can be accounted for.

Within these limitations, however, we note that even for a hypothetical "(THTZ-H)PbI_3" perovskite the Goldschmidt tolerance factor calculated using our steric radii is less than the 'ideal' value of 1, but significantly larger than all reported $APbI_3$ crystals with 100% A-site occupancy.

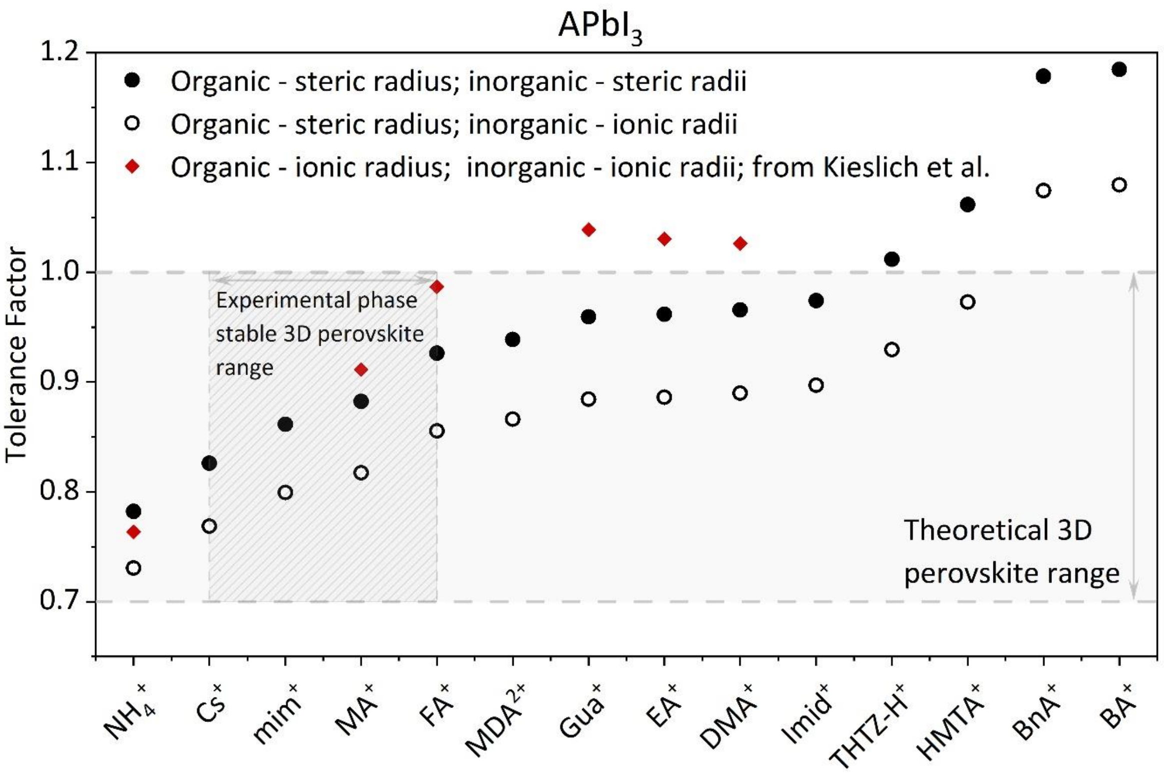

Figure S23. Goldschmidt Tolerance factor calculated using our steric radii for organic cations with Shannon radii (closed circles) and DFT radii (open circles) of Pb^{2+} and I^-, and using the effective ionic radii reported by Kieslich et al.[24] alongside Shannon radii of Pb^{2+} and I^-. The steric radii of different A-site cations are obtained from DFT calculations, as explained above. The grey shaded area represents the zone of stability of $APbI_3$ perovskites with lower limit of 0.74, obtained from TL1 and TL2 (see Table S1 in the Supplementary Material of Filip et al.[23]). The experimentally-reported phase stable region for 3D ABI_3 perovskites (with only one A-site cation) is between Cs^+ (lower) and FA^+ (upper), as highlighted. Both $CsPbI_3$ and $FAPbI_3$, have a metastable 3D cubic perovskite phase under ambient conditions.

Section S8: Solid-state Nuclear Magnetic Resonance Spectroscopy

The single crystal samples were packed into 1.3 mm (1 GHz, ^{1}H measurements), 3.2 mm (500 MHz, ^{14}N measurements) or 4 mm (600 MHz, ^{13}C measurements and ^{127}I NQR) zirconia rotors. The samples were compacted to enable stable spinning which necessarily led to the crystals being crushed. We note that the α-phase stability of all crystals is reduced by the crushing process. It is unclear if this is due to the application of pressure itself, or due to increased exposure to airborne moisture as a result of the increased surface area of the crushed material. We find that instantaneous phase degradation is suppressed by crushing in a N_2 glovebox, suggesting that moisture and/or oxygen play at least some role.

Solid-state MAS NMR spectra of ^{1}H (1000.4 MHz) were recorded on a Bruker Avance Neo 23.5 T spectrometer equipped with a 1.3 mm MAS probe using 100 kHz RF field amplitude. ^{1}H chemical shifts were referenced to TMS using the residual GBL signal as an internal reference (4.35 ppm for $-CH_2-O-$). Quantitative ^{1}H spectra were recorded using a recycle delay of 300 s (The longest T_1 time of 40-50 s corresponded to the FA moieties). ^{1}H-^{1}H spin-diffusion spectra were recorded with a recycle delay of 2-3 s, mixing time of 2-3 s, and 600 slices in the indirect dimension. ^{13}C MAS spectra were recorded on a Bruker Avance III 11.7 T (150.7 MHz) spectrometer equipped with a 4 mm CPMAS probe using 71 kHz RF field amplitude, a recycle delay of 10 s, and referenced to solid adamantane (38.48 ppm for $-CH_2-$). 74 kHz ^{1}H decoupling was used. ^{14}N MAS spectra were recorded on a Bruker Avance III 11.7 T (43.3 MHz) spectrometer equipped with a 3.2 mm CPMAS probe using 25 kHz RF field amplitude, a recycle delay of 0.1 s, and referenced to solid adamantane using the ratio of gyromagnetic ratios in accordance with the IUPAC recommendation. The rotors were spun using dry nitrogen. ^{127}I NQR spectra were recorded on a Bruker Avance III spectrometer equipped with a 4 mm CPMAS probe using 42 kHz RF field amplitude, a recycle delay of 0.05 s with the probe placed outside the stray field of the magnet. For NQR measurements, the sample was static.

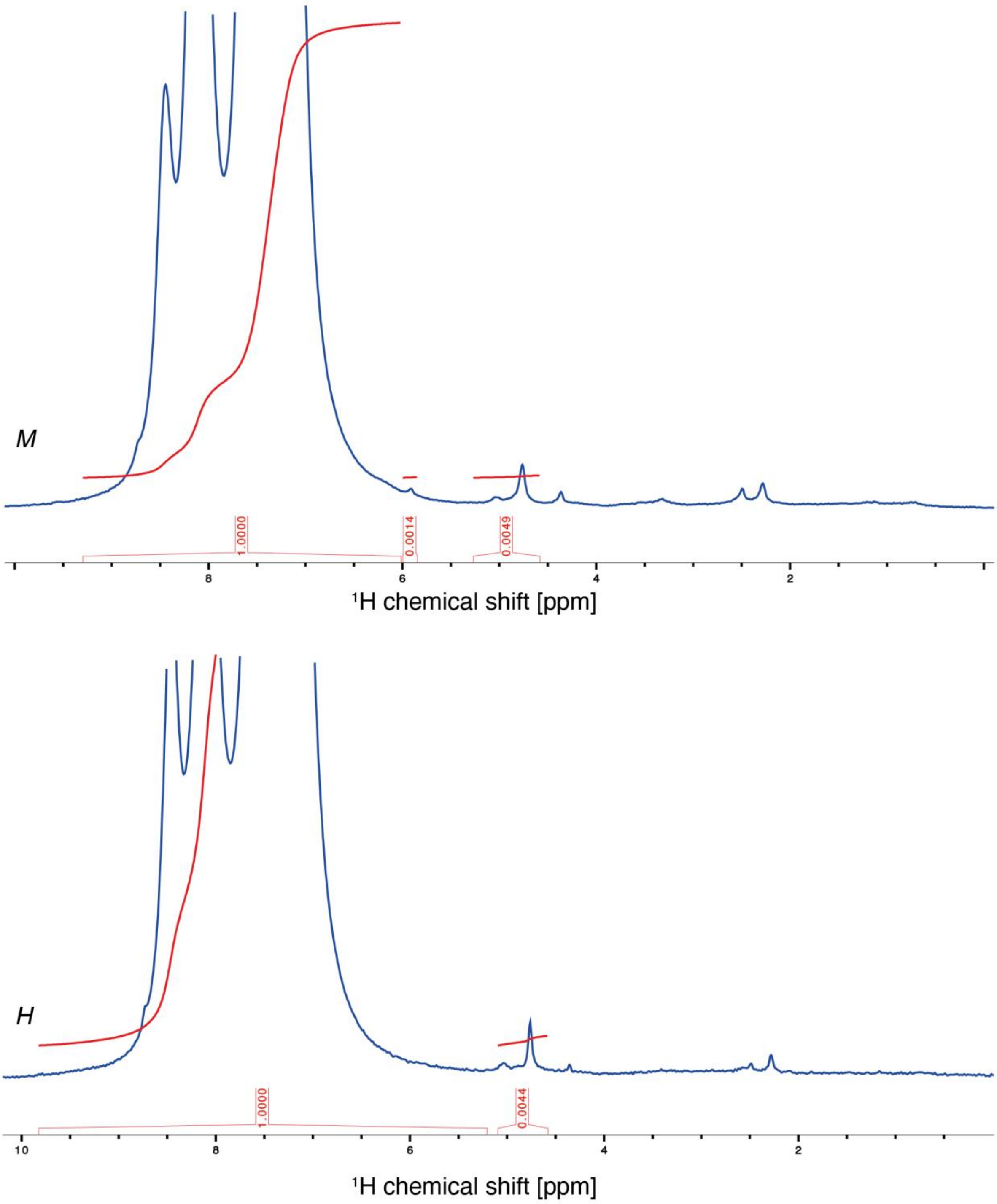

Figure S24: Quantitative ^{1}H MAS (50 kHz) NMR spectra of FAPbI$_3$-H and FAPbI$_3$-M crystals. Integration of the selected regions indicates that the new species is present at ~0.5 mol% relative to FA in both materials, assuming that the number of protons in FA and in the new species is the same.

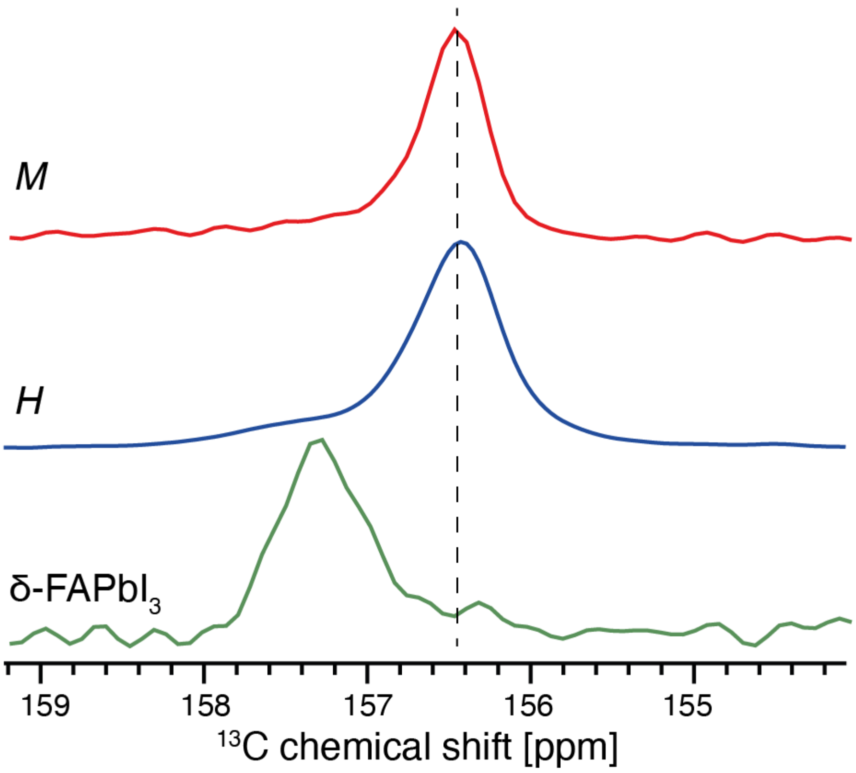

Figure S25: Comparison of the ^{13}C signatures of FA in the δ (yellow) and black phases. ^{13}C echo-detected NMR spectra (12 kHz MAS, 14 T) of yellow δ-$FAPbI_3$ (obtained from reference α-$FAPbI_3$ crystals that transformed in air) and the black $FAPbI_3$-H and $FAPbI_3$-M crystals.

Structural model of $FAPbI_3$-H based on the ^1H-^1H spin-diffusion spectrum

^1H-^1H spin diffusion spectra enable establishing atomic-level contact between species in solid materials. This is because for the cross-peaks to appear, there needs to be a pathway for the ^1H magnetization to travel between the two species. This can only be the case when the two species are present within the same phase, within a network of dipolar coupled ^1H spins. In our experiment, we observe cross-peaks between GBL and THTZ-H^+, THTZ-H^+ and FA, and GBL and FA. This result indicates that all three components are present within the same phase, and not as separate phases.

Conceptually, there are a number of structural scenarios in which impurities can be incorporated into crystalline materials: surface absorption, solid solution, co-crystal (for molecular solids), agglomeration or inclusion[43]. In each of these cases, the impurities are in atomic-level contact with the perovskite lattice and would lead to the appearance of cross-peaks in spin-diffusion spectra. We identify the incorporation mechanism based on the other NMR results, as follows. The ^{127}I NQR spectrum of $FAPbI_3$-H is qualitatively different (broader) compared to that of reference $FAPbI_3$, which indicates that all iodide sites within the crystal are affected by the presence of THTZ -H^+. This would not be the case if THTZ-H^+ was merely adsorbed on the surface of the crystal or kinetically trapped in a pocket of mother liquor during crystallization (as is the case during agglomeration and formation of crystal inclusions). The formation of a solid solution is therefore the only mechanism that agrees with the experimental ^1H-^1H spin-diffusion and ^{127}I NQR data. On the other hand, the presence of GBL within all three types of crystals (reference $FAPbI_3$, $FAPbI_3$-H and $FAPbI_3$-M) indicates that the solvent is kinetically trapped during crystallization as inclusions which exist independently of the THTZ -H^+ (note that those inclusions are present also in the reference $FAPbI_3$ which has no THTZ -H^+).

For completeness, we also consider the hypothesis that THTZ-H^+ could template the growth of $FAPbI_3$ such that the resulting perovskite lattice is more disordered relative to undoped $FAPbI_3$ (octahedral tilting induced by surface templating, as reported by Doherty et al.[44]). In this scenario, the dopant would only be present on the crystal surface but still lead to a qualitative change in the local iodide environments, as probed by ^{127}I NQR, throughout the crystal volume. While our results could be consistent with this hypothesis, we present a series of arguments – with supporting experimentation – from which we eliminate this possibility.

First, we note the relatively large THTZ-H^+ content in the crystals (ca. 0.5 mol% relative to FA). Considering the low surface area of the crystals (on the order of 0.001 m^2/g), the amount of incorporated THTZ-H^+ is several orders of magnitude larger relative to what would be expected for a surface monolayer. For example, considering a cubic $FAPbI_3$ crystal with an edge length of 1 mm and a density of 4.16 g cm^{-3}, a uniform layer of THTZ-H^+ (assuming density 1.5 g cm^{-3} and a FA:THTZ-H^+ molar ratio of 1:0.005) on the cube surface would have a thickness of about 100 μm. Such a thick surface layer of THTZ-H^+ (or THTZ iodoplumbate) would be expected to diffract X-rays, but we did not detect any such additional phases our XRD experiments. Further, it might be expected that such a capping layer could be removed by an appropriate solvent. We washed away the exterior layers of $FAPbI_3$-M and $FAPbI_3$-H crystals by repeated submersion in GBL (three washes) and conducted ^{1}H NMR solution NMR measurements on the resultant crystals. As shown in **Supplementary Figure S21** the washing process did not remove signals corresponding to THTZ-H^+. Quantitative analysis of a reduction in THTZ-H^+ content by this treatment is impossible due to crystal-to-crystal variation, as discussed above. It is possible that our washing process failed to remove all surface features or that THTZ-H^+ remaining in the washing solvent is retained on the surface during drying. However, we further note from our Raman spectroscopy that we do not observe that the use of either HMTA or $MDACl_2$ additives has any effect on the position and width of the Raman modes of $FAPbI_3$, as might be expected in the presence of substantial non-FA^+ organic components at the surface[45] (**Supplementary Figure S10**). Finally, we observe that crystals cleaved to reveal the bulk α-$FAPbI_3$-M and α-$FAPbI_3$-H material do not degrade at an enhanced rate (**Figure 1e**) as would be expected if these surfaces did not benefit from the hypothesised capping layer.

Section S9: Electron Probe Microanalysis (EPMA)

Electron probe micro-analyses were performed on a Cameca SX-5 FEG in the Department of Earth Sciences at the University of Oxford. Samples were mounted in epoxy resin, ground flat and polished under oil, using a combination of silicon carbide and diamond laps. Samples were coated with ~20 nm of carbon to minimise any charge build-up during the analysis. A 15 kV accelerating voltage was employed, with beam currents between 20 and 24 nA and a nominal spot size of 10 μm. Cl, I, and Pb were quantified using NaCl, Tl(Br,I), and galena (PbS) standards respectively. On-peak counting times for Cl (Kα) and Pb (Mα) were 60 s and 45 s for I (Lα), with half of on-peak counting times at each of the high and low background positions. Using these conditions the observed intensity of I X-rays is time-dependent, decreasing somewhat throughout the analysis. Thus, we applied a time dependent intensity (TDI) correction (e.g., Nielsen et al.[46]) to the I X-ray intensity whereby the on-peak intensity was sub-divided into 22 equal time segments and the count-rate extrapolated back to the intensity at the start of the analysis (T=0). An overlap correction of Pb (Mα) on Cl (Kα) was corrected using nominally Cl-free galena. The concentration of hydrogen, carbon, and nitrogen were added as knowns (based on an approximately pure FA^+ organic composition) to the f(rz) matrix-correction routine employed.

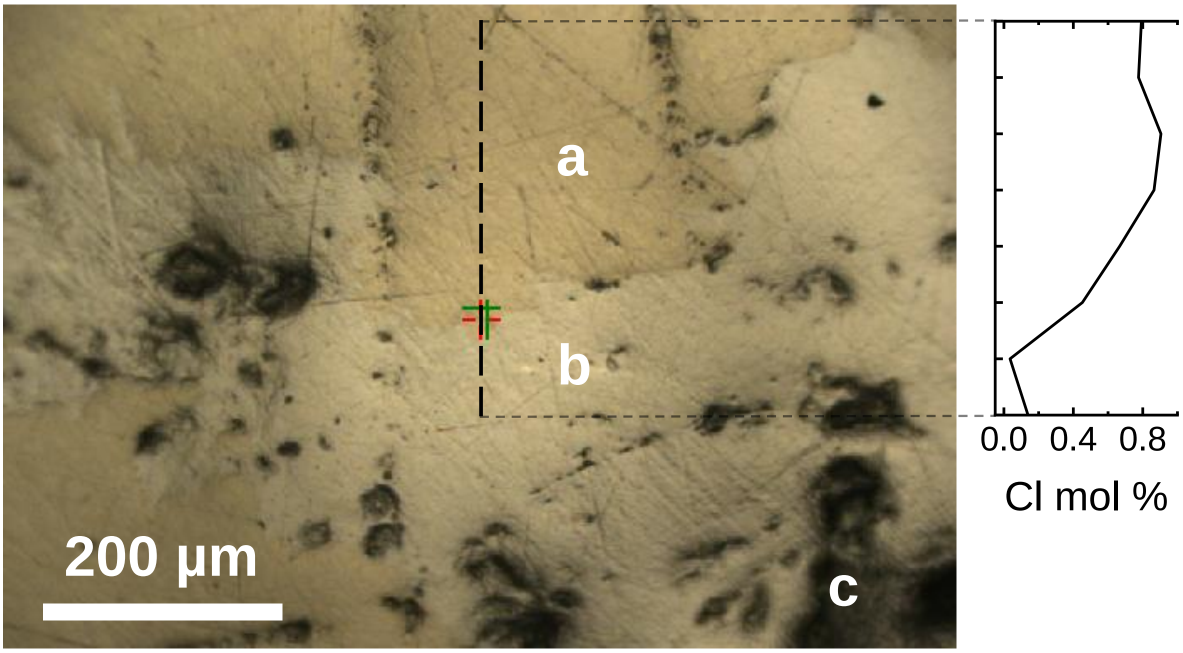

Figure S26: Reflected light microscope image of cleaved, polished $FAPbI_3$-M crystal showing regions of Cl-rich (a, light brown) and Cl-depleted (b, golden) perovskite material. Lineplot tracks Cl mol% (as proportion of total halide content) as the EPMA measurement is carried out along a series of points traversing from a region Cl-depleted to Cl-rich material. Also highlighted is a region where material has been incidentally removed during the polishing process (c). We note that this damage occurs far more readily in the chloride-depleted region, suggesting that the material may be softer here. Also visible in the image are scratch marks, introduced during polishing.

The concentration of Cl in the $FAPbI_3$-M crystals is ~1300 ppm (average of 62 point measurements), compared to a detection limit of approximately 130 ppm. The neat crystals have little to no detectable Cl; an average of 42 point measurements gives a concentration of only 50 ppm, below the sensitivity of this technique.

S10: References

(1) Nayak, P. K.; Moore, D. T.; Wenger, B.; Nayak, S.; Haghighirad, A. A.; Fineberg, A.; Noel, N. K.; Reid, O. G.; Rumbles, G.; Kukura, P.; Vincent, K. A.; Snaith, H. J. Mechanism for Rapid Growth of Organic-Inorganic Halide Perovskite Crystals. *Nat. Commun.* **2016**, *7*, 1–8.

(2) Wenger, B.; Nayak, P. K.; Wen, X.; Kesava, S. V; Noel, N. K.; Snaith, H. J. Consolidation of the Optoelectronic Properties of $CH_3NH_3PbBr_3$ Perovskite Single Crystals. *Nat. Commun.* **2017**, *8* (1), 590.

(3) Weller, M. T.; Weber, O. J.; Frost, J. M.; Walsh, A. Cubic Perovskite Structure of Black Formamidinium Lead Iodide, α-$[HC(NH_2)_2]PbI_3$, at 298 K. *J. Phys. Chem. Lett.* **2015**, *6* (16), 3209–3212.

(4) Uller Rothmann, M.; Kim, J. S.; Borchert, J.; Lohmann, K. B.; O'Leary, C. M.; Sheader, A. A.; Clark, L.; Snaith, H. J.; Johnston, M. B.; Nellist, P. D.; Herz, L. M. Atomic-Scale Microstructure of Metal Halide Perovskite. *Science.* **2020**, *370* (6516).

(5) Zhang, X.; Wang, F.; Zhang, B. Bin; Zha, G.; Jie, W. Ferroelastic Domains in a CsPbBr3Single Crystal and Their Phase Transition Characteristics: An in Situ TEM Study. *Cryst. Growth Des.* **2020**, *20* (7), 4585–4592.

(6) Rothmann, M. U.; Li, W.; Zhu, Y.; Bach, U.; Spiccia, L.; Etheridge, J.; Cheng, Y. B. Direct Observation of Intrinsic Twin Domains in Tetragonal $CH_3NH_3PbI_3$. *Nat. Commun.* **2017**, *8*, 6–13.

(7) Wright, A. D.; Buizza, L. R. V.; Savill, K. J.; Longo, G.; Snaith, H. J.; Johnston, M. B.; Herz, L. M. Ultrafast Excited-State Localization in $Cs_2AgBiBr_6$ Double Perovskite. *J. Phys. Chem. Lett.* **2021**, *12* (13), 3352–3360.

(8) Lindsey, C. P. .; Patterson, G. D. Detailed Comparison of the Williams–Watts and Cole–Davidson Functions. *J. Chem. Phys.* **1980**, *73* (7), 3348–3357.

(9) Xia, C. Q.; Peng, J.; Poncé, S.; Patel, J. B.; Wright, A. D.; Crothers, T. W.; Uller Rothmann, M.; Borchert, J.; Milot, R. L.; Kraus, H.; Lin, Q.; Giustino, F.; Herz, L. M.; Johnston, M. B. Limits to Electrical Mobility in Lead-Halide Perovskite Semiconductors. *J. Phys. Chem. Lett.* **2021**, *12* (14), 3607–3617.

(10) Lim, J.; Kober-Czerny, M.; Lin, Y. H.; Ball, J. M.; Sakai, N.; Duijnstee, E. A.; Hong, M. J.; Labram, J. G.; Wenger, B.; Snaith, H. J. Long-Range Charge Carrier Mobility in Metal Halide Perovskite Thin-Films and Single Crystals via Transient Photo-Conductivity. *Nat. Commun.* **2022**, *13* (1), 1–9.

(11) Lim, J.; Hörantner, M. T.; Sakai, N.; Ball, J. M.; Mahesh, S.; Noel, N. K.; Lin, Y. H.; Patel, J. B.; McMeekin, D. P.; Johnston, M. B.; Wenger, B.; Snaith, H. J. Elucidating the Long-Range Charge Carrier Mobility in Metal Halide Perovskite Thin Films. *Energy Environ. Sci.* **2019**, *12* (1), 169–176.

(12) Han, Q.; Bae, S. H.; Sun, P.; Hsieh, Y. T.; Yang, Y.; Rim, Y. S.; Zhao, H.; Chen, Q.; Shi, W.; Li, G.; Yeng, Y. Single Crystal Formamidinium Lead Iodide (FAPbI3): Insight into the Structural, Optical, and Electrical Properties. *Adv. Mater.* **2016**, *28* (11), 2253–2258.

(13) Steele, J. A.; Yuan, H.; Tan, C. Y. X.; Keshavarz, M.; Steuwe, C.; Roeffaers, M. B. J.; Hofkens, J. Direct Laser Writing of δ- To α-Phase Transformation in Formamidinium Lead Iodide. *ACS Nano* **2017**, *11* (8), 8072–8083.

(14) Kontos, A. G.; Manolis, G. K.; Kaltzoglou, A.; Palles, D.; Kamitsos, E. I.; Kanatzidis, M. G.; Falaras, P. Halogen-NH_2^+ Interaction, Temperature-Induced Phase Transition, and Ordering in $(NH_2CHNH_2)PbX_3$ (X = Cl, Br, I) Hybrid Perovskites. *J. Phys. Chem. C* **2020**, *124* (16), 8479–8487.

(15) Erkılıç, U.; Ji, H. G.; Nishibori, E.; Ago, H. One-Step Vapour Phase Growth of Two-Dimensional Formamidinium-Based Perovskite and Its Hot Carrier Dynamics. *Phys. Chem. Chem. Phys.* **2020**, *22* (37), 21512–21519.

(16) Driscoll, E. H.; Orera, A.; Anderson, P. A.; Sanjuán, M. L.; Slater, P. R. Raman Spectroscopy Insights into the α- And δ-Phases of Formamidinium Lead Iodide ($FAPbI_3$). *Dalt. Trans.* **2021**, *50* (9), 3315–3323.

(17) Ibaceta-Jaña, J.; Muydinov, R.; Rosado, P.; Vinoth Kumar, S. H. B.; Gunder, R.; Hoffmann, A.; Szyszka, B.; Wagner, M. R. Hidden Polymorphism of $FAPbI_3$ discovered by Raman Spectroscopy. *Phys. Chem. Chem. Phys.* **2021**, *23* (15), 9476–9482.

(18) Doddrell, D. M.; Pegg, D. T.; Bendall, M. R. Distortionless Enhancement of NMR Signals by Polarization Transfer. *J. Magn. Reson.* **1982**, *48* (2), 323–327.

(19) Kintzinger, J. P.; Lehn, J. M. Nuclear Spin-Spin Interactions . Effect of Nitrogen Lone-Pair / = YoH. *Chem. Commun.* **1967**, No. 13, 660–661.

(20) Mayr, H.; Ofial, A. R.; Würthwein, E. U.; Aust, N. C. NMR Spectroscopic Evidence for the Structure of Iminium Ion Pairs. *J. Am. Chem. Soc.* **1997**, *119* (52), 12727–12733.

(21) Zeffiro, A.; Lazzaroni, S.; Merli, D.; Profumo, A.; Buttafava, A.; Serpone, N.; Dondi, D. Formation of Hexamethylenetetramine (HMT) from HCHO and NH_3 – Relevance to Prebiotic Chemistry and B3LYP Consideration. *Orig. Life Evol. Biosph.* **2016**, *46* (2–3), 223–231.

(22) Van Gompel, W. T. M.; Herckens, R.; Reekmans, G.; Ruttens, B.; D'Haen, J.; Adriaensens, P.; Lutsen, L.; Vanderzande, D. Degradation of the Formamidinium Cation and the Quantification of the Formamidinium-Methylammonium Ratio in Lead Iodide Hybrid Perovskites by Nuclear Magnetic Resonance Spectroscopy. *J. Phys. Chem. C* **2018**, *122* (8), 4117–4124.

(23) Filip, M. R.; Eperon, G. E.; Snaith, H. J.; Giustino, F. Steric Engineering of Metal-Halide Perovskites with Tunable Optical Band Gaps. *Nat. Commun.* **2014**, *5*.

(24) Kieslich, G.; Sun, S.; Cheetham, A. K. Solid-State Principles Applied to Organic–Inorganic Perovskites: New Tricks for an Old Dog. *Chem. Sci.* **2014**, *5* (12), 4712–4715.

(25) Gholipour, S.; Ali, A. M.; Correa-Baena, J. P.; Turren-Cruz, S. H.; Tajabadi, F.; Tress, W.; Taghavinia, N.; Grätzel, M.; Abate, A.; De Angelis, F.; Gaggioli, C. A.; Mosconi, E.; Hagfeldt, A.; Saliba, M. Globularity-Selected Large Molecules for a New Generation of Multication Perovskites. *Adv. Mater.* **2017**, *29* (38), 1–9.

(26) Chu, C. H.; Leung, C. W. Inhomogeneous Electron Gas. *Phys. Rev.* **1964**, *136* (3B), 864–871.

(27) Giannozzi, P.; Andreussi, O.; Brumme, T.; Bunau, O.; Nardelli, M. B.; Calandra, M.; Car, R.; Cavazzoni, C.; Ceresoli, D.; Cococcioni, M.; others. Advanced Capabilities for Materials Modelling with Quantum ESPRESSO. *J. Phys. Condens. Matter* **2017**, *29* (465901).

(28) Perdew, J. P.; Burke, K.; Ernzerhof, M. Generalized Gradient Approximation Made Simple. *Phys. Rev. Lett.* **1996**, *77* (18), 3865–3868.

(29) Hamann, D. R. Optimized Norm-Conserving Vanderbilt Pseudopotentials. *Phys. Rev. B - Condens. Matter Mater. Phys.* **2013**, *88* (8), 1–10.

(30) van Setten, M. J.; Giantomassi, M.; Bousquet, E.; Verstraete, M. J.; Hamann, D. R.; Gonze, X.; Rignanese, G. M. The PSEUDODOJO: Training and Grading a 85 Element Optimized Norm-Conserving Pseudopotential Table. *Comput. Phys. Commun.* **2018**, *226*, 39–54.

(31) Qiao, W. C.; Liang, J. Q.; Dong, W.; Ma, K.; Wang, X. L.; Yao, Y. F. Formamidinium Lead Triiodide Perovskites with Improved Structural Stabilities and Photovoltaic Properties Obtained by Ultratrace Dimethylamine Substitution. *NPG Asia Mater.* **2022**, *14* (1).

(32) Marshall, A. R.; Sansom, H. C.; McCarthy, M. M.; Warby, J. H.; Ashton, O. J.; Wenger, B.; Snaith, H. J. Dimethylammonium: An A-Site Cation for Modifying $CsPbI_3$. *Sol. RRL* **2021**, *5* (1).

(33) Eperon, G. E.; Stone, K. H.; Mundt, L. E.; Schloemer, T. H.; Habisreutinger, S. N.; Dunfield, S. P.; Schelhas, L. T.; Berry, J. J.; Moore, D. T.; Eperon, G. E.; Moore, D. T. The Role of Dimethylammonium in Bandgap Modulation for Stable Halide Perovskites. *ACS Energy Lett.* **2020**, *5* (6), 1856–1864.

(34) Kubicki, D. J.; Prochowicz, D.; Hofstetter, A.; Saski, M.; Yadav, P.; Bi, D.; Pellet, N.; Lewiński,

J.; Zakeeruddin, S. M.; Grätzel, M.; Emsley, L. Formation of Stable Mixed Guanidinium-Methylammonium Phases with Exceptionally Long Carrier Lifetimes for High-Efficiency Lead Iodide-Based Perovskite Photovoltaics. *J. Am. Chem. Soc.* **2018**, *140* (9), 3345–3351.

(35) Sutton, R. J.; Filip, M. R.; Haghighirad, A. A.; Sakai, N.; Wenger, B.; Giustino, F.; Snaith, H. J. Cubic or Orthorhombic? Revealing the Crystal Structure of Metastable Black-Phase $CsPbI_3$ by Theory and Experiment. *ACS Energy Lett.* **2018**, *3* (8), 1787–1794.

(36) Lee, M. M.; Teuscher, J.; Miyasaka, T.; Murakami, T. N.; Snaith, H. J. Efficient Hybrid Solar Cells Based on Meso-Superstructured Organometal Halide Perovskites. *Science.* **2012**, *338* (6107), 643–647.

(37) Eperon, G. E.; Stranks, S. D.; Menelaou, C.; Johnston, M. B.; Herz, L. M.; Snaith, H. J. Formamidinium Lead Trihalide: A Broadly Tunable Perovskite for Efficient Planar Heterojunction Solar Cells. *Energy Environ. Sci.* **2014**, *7* (3), 982–988.

(38) Kim, J.; Hwang, T.; Lee, B.; Lee, S.; Park, K.; Park, H. H.; Park, B. An Aromatic Diamine Molecule as the A-Site Solute for Highly Durable and Efficient Perovskite Solar Cells. *Small Methods* **2019**, *3* (1), 1–6.

(39) Goldschmidt, V. M. Die Gesetze Der Krystallochemie. *Naturwissenschaften* **1926**, *14* (21), 477–485.

(40) Bartel, C. J.; Sutton, C.; Goldsmith, B. R.; Ouyang, R.; Musgrave, C. B.; Ghiringhelli, L. M.; Scheffler, M. New Tolerance Factor to Predict the Stability of Perovskite Oxides and Halides. *Sci. Adv.* **2019**, *5* (2), 1–10.

(41) Li, C.; Soh, K. C. K.; Wu, P. Formability of ABO3 Perovskites. *J. Alloys Compd.* **2004**, *372* (1–2), 40–48.

(42) Li, C.; Lu, X.; Ding, W.; Feng, L.; Gao, Y.; Guo, Z. Formability of ABX_3 (X = F, Cl, Br, I) Halide Perovskites. *Acta Crystallogr. Sect. B Struct. Sci.* **2008**, *64* (6), 702–707.

(43) Urwin, S. J.; Levilain, G.; Marziano, I.; Merritt, J. M.; Houson, I.; Ter Horst, J. H. A Structured Approach to Cope with Impurities during Industrial Crystallization Development. *Org. Process Res. Dev.* **2020**, *24* (8), 1443–1456.

(44) Doherty, T. A. S.; Nagane, S.; Kubicki, D. J.; Jung, Y. K.; Johnstone, D. N.; Iqbal, A. N.; Guo, D.; Frohna, K.; Danaie, M.; Tennyson, E. M.; Macpherson, S.; Abfalterer, A.; Anaya, M.; Chiang, Y. H.; Crout, P.; Ruggeri, F. S.; Collins, S. M.; Grey, C. P.; Walsh, A.; Midgley, P. A.; Stranks, S. D. Stabilized Tilted-Octahedra Halide Perovskites Inhibit Local Formation of Performance-Limiting Phases. *Science.* **2021**, *374* (6575), 1598–1605.

(45) Pérez-Osorio, M. A.; Lin, Q.; Phillips, R. T.; Milot, R. L.; Herz, L. M.; Johnston, M. B.; Giustino, F. Raman Spectrum of the Organic-Inorganic Halide Perovskite $CH_3NH_3PbI_3$ from First Principles and High-Resolution Low-Temperature Raman Measurements. *J. Phys. Chem. C* **2018**, *122* (38), 21703–21717.

(46) Nielsen, C. H.; Sigurdsson, H. Quantitative Methods for Electron Microprobe Analysis of Sodium in Natural and Synthetic Glasses. *Am. Mineral.* **1981**, *66* (5–6), 547–552.
